# The marrow niche controls the cancer stem cell phenotype of disseminated prostate cancer

**DOI:** 10.18632/oncotarget.9251

**Published:** 2016-05-09

**Authors:** Yusuke Shiozawa, Janice E. Berry, Matthew R. Eber, Younghun Jung, Kenji Yumoto, Frank C. Cackowski, Hyeun Joong Yoon, Princy Parsana, Rohit Mehra, Jingcheng Wang, Samantha McGee, Eunsohl Lee, Sunitha Nagrath, Kenneth J. Pienta, Russell S. Taichman

**Affiliations:** ^1^ Department of Periodontics and Oral Medicine, University of Michigan School of Dentistry, Ann Arbor, MI 48109, USA; ^2^ Department of Cancer Biology and Comprehensive Cancer Center, Wake Forest University School of Medicine, Winston-Salem, NC 27157, USA; ^3^ Department of Chemical Engineering, University of Michigan, Ann Arbor, MI 48109, USA; ^4^ Department of Urology, The James Buchanan Brady Urological Institute, Johns Hopkins School of Medicine, Baltimore, MD 21287, USA; ^5^ Department of Computer Science, Johns Hopkins University, Baltimore, MD 21218, USA; ^6^ Department of Anatomic Pathology, University of Michigan, Ann Arbor, MI 48109, USA

**Keywords:** prostate cancer, disseminated tumor cells, bone marrow microenvironment, hematopoietic stem cell niche, cancer stem cells

## Abstract

Dissemination of cancer stem cells (CSCs) serves as the basis of metastasis. Recently, we demonstrated that circulating prostate cancer targets the hematopoietic stem cell (HSCs) ‘niche’ in marrow during dissemination. Once in the niche, disseminated tumor cells (DTCs) may remain dormant for extended periods. As the major function of the HSC niche is to maintain stem cell functions, we hypothesized that the niche regulates CSC activities of DTCs. Here we show that DTCs recovered from marrow were significantly enriched for a CSC phenotype. Critically, the conversion of DTCs to CSCs is regulated by niche-derived GAS6 through the Mer/mTOR; molecules previously shown to regulate dormancy. The data demonstrate that the niche plays a significant role in maintaining tumor-initiating prostate cancer in marrow and suggests a functional relationship between CSCs and dormancy. Understanding how the marrow niche regulates the conversion of DTCs to CSCs is critical for the development of therapeutics specifically targeting skeletal bone metastasis and dormancy.

## INTRODUCTION

Every year, patients who thought they were cured of prostate cancer by radiation or surgery present with incurable metastatic disease in their skeleton [1]. Previously, we showed that circulating prostate cancer targets the bone marrow ‘niche’ that houses hematopoietic stem cells (HSCs) and, critically, that disseminated tumor cells (DTCs) compete with HSCs for occupancy of that niche [2]. Once in the niche, DTCs may remain viable for extended periods [3–7], yet little is understood about how disseminated prostate cancer remains viable and later develop into skeletal metastases. Many believe cancer stem-like cells (CSCs) are culpable.

The hypothesis that tumors depend on a small fraction of cells, or CSCs, for long-term survival was proposed based upon data demonstrating that subsets of human leukemic cells transferred tumor-initiating activities to SCID mice [8]. More recent experimentation suggests that CSCs have the ability to self-renew and to generate multiple ‘mature’ tumor progeny [9]. Generally, freshly isolated CSCs exhibit low proliferative activity and, as a result, possess chemo- and radio-resistance [10]. From these observations, it has been assumed that CSCs are typically dormant, and their later regrowth is responsible for metastases. As the molecular machinery of the HSC niche is designed to regulate stem cell quiescence and self-renewal [11], we therefore hypothesized that DTCs may be converted to a CSC phenotype through engagement with the niche, thus establishing a future site of metastasis.

Using murine models of human metastasis, we show that DTCs recovered from marrow are significantly enriched for a CSC phenotype. The conversion to CSCs was observed in DTCs following the injection of only non-CSCs, and occurred primarily in the marrow. The CSCs in marrow were maintained over time, and was not due to effects on proliferation, homing, or cell survival in the circulation. Importantly, growth arrest specific 6 (GAS6), which influences prostate cancer dormancy [12–14], and is secreted by the osteoblastic niche [12], regulates part of the conversion of DTCs into CSCs through its receptor Mer, by activating the mTOR signaling pathway following cell-to-cell contact. These data demonstrate that the HSC niche plays a significant role in the production and maintenance of tumor-initiating CSCs in marrow.

## RESULTS

### Disseminated prostate cancer are converted to the CSC within the marrow microenvironment

**Figure 1:**
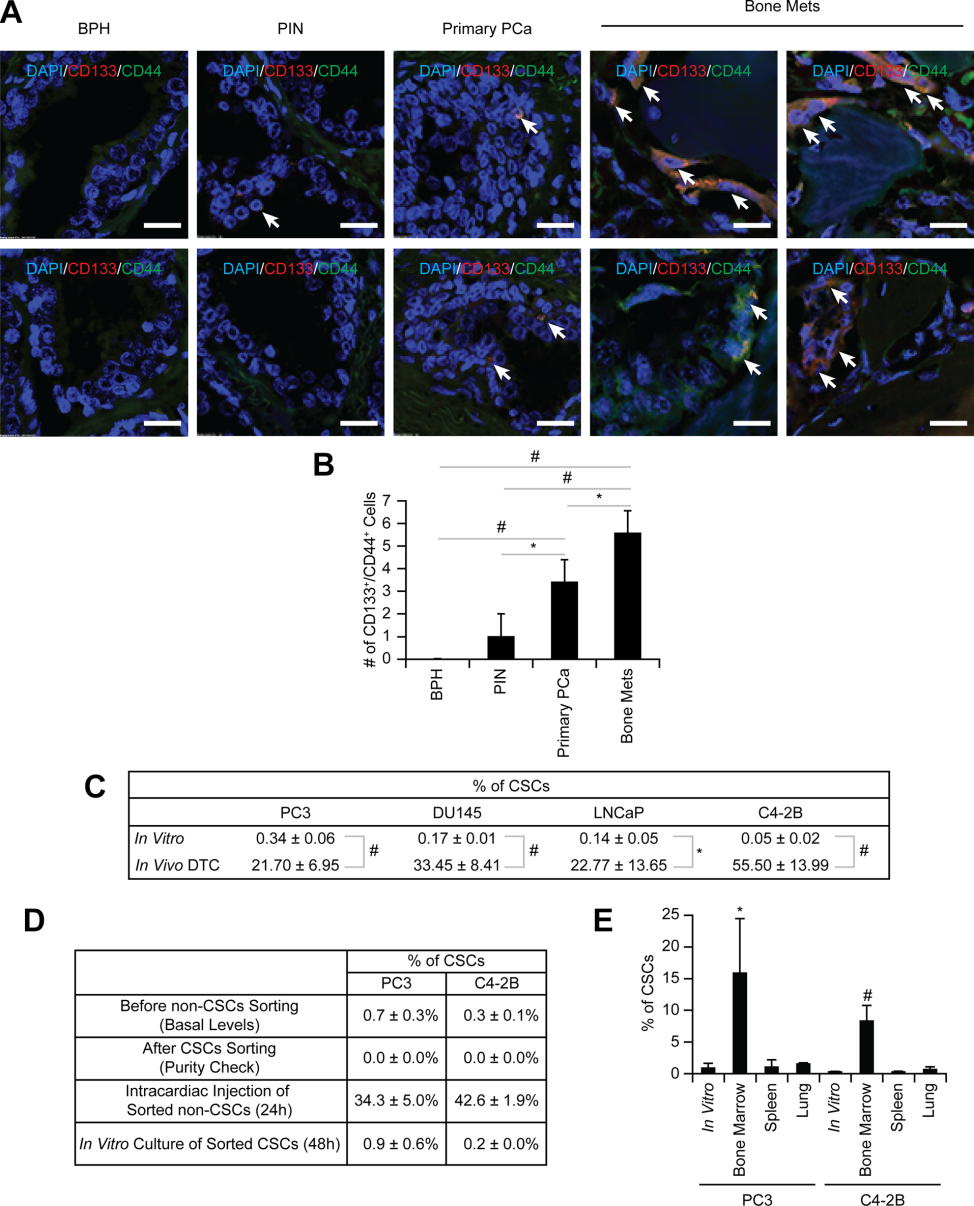
Enrichment of CSCs in disseminated prostate cancer (**A**) Representative elements of a prostate cancer tissue microarray co-stained with anti-CD133 and anti-CD44 antibodies. Nuclei were identified by DAPI. (60× Zoom2, Bar = 20 μm). (**B**) Quantitative analysis of CSC staining in Figure 1A. ^*^p < 0.05 and ^#^*p* < 0.01 (Student's *t*-test). BPH: benign prostatic hyperplasia; PIN: prostatic intraepithelial neoplasia; Primary prostate cancer: primary prostate cancer; and Bone mets: bone metastatic prostate cancer. (**C**) The % of CSC population in *in vitro* cultured prostate cancer and *in vivo* DTCs by flow cytometry. Significance vs. *in vitro* cultured prostate cancer (Student's *t* test). (**D**) Non-CSC prostate cancer cells were sorted and subsequent flow cytometry analyses confirmed no residual contamination of CSC cells. Pure non-CSCs were either inoculated into SCID mice through intracardiac injection (*n* = 5) or cultured *in vitro*, and the CSC population was analyzed. (**E**) CSC expression in prostate cancer recovered from the lung, spleen, or bone marrow following intracardiac injection (*n* = 5). *p < 0.05 and ^#^*p* < 0.01 vs. *in vitro* cultured prostate cancer (Student's *t* test).

Previously we showed that early in the metastatic process prostate cancer targets and commandeers the marrow microenvironment or “niche” which houses HSCs, using mechanisms similar to those involved in HSC homing [2]. Subsequently, these disseminated prostate cancer parasitizes this microenvironment to become dormant and survive within the marrow [12]. Since the major function of the HSC niche is to maintain stem cell functions, we hypothesized that engagement of DTCs within the niche regulates CSC activities. To test our hypothesis, the expression of CD133 and CD44 was first analyzed on tissue microarrays from prostate cancer patients since prostate cancer expressing both CD133 and CD44 represent a rare population of cells with stem cell-like properties [15]. Intriguingly, the number of CSCs (CD133^+^/CD44^+^) was enhanced with increasing tumor grade (Figure 1A–1B).

To further explore how the niche regulates CSC activities we developed a reproducible assay in which DTCs derived from human tumors grown in SCID mice can be recovered with high fidelity from murine marrow. We found that unlike EpCAM and cytokeratin, antibodies targeting HLA-ABC represent a stable and suitable approach for capturing DTCs. First, we used flow cytometry to evaluate the basal levels of cytokeratin and EpCAM on the surface of human prostate cancer cell lines (PC3, DU145, LNCaP, and C4- 2B), with leukemia cell lines (RCH-ACV, 697, Nalm6 and RS4;11) as controls. Prostate cancer cells expressed low or moderate levels of cytokeratin and high levels of EpCAM *in vitro*, while levels of both were low in the leukemia lines (Figure S1A). However, since the cytokeratin and EpCAM levels were variable, we explored using HLA expression as an alternative molecular probe for recovery of human DTCs from murine marrow. We found that the HLA-ABC antigen was highly expressed on all prostate cancer cells tested (Figure S1A). Therefore intracardiac injections of prostate cancer cells into SCID mice were performed to determine if human prostate cancer cells could be isolated from murine marrow using anti-HLA-ABC antibodies (Figure S1B). Depending on the cell line examined, 10.5 ± 2.3% to 16.7 ± 2.5% of the lineage depleted marrow cells expressed HLA-ABC 24 hours post injection (Figure S1C). As previously described [16], the expression of cytokeratin and EpCAM varied considerably *in vitro* (Figure S1A) and *in vivo* (Figure S1D), however almost all cytokeratin (Figure S1E) and EpCAM positive prostate cancer cells (Figure S1F) expressed HLA-ABC on their surface. This strategy was validated in two ways: First, prostate cancer cells injected intratibially were visualized after 24 hours *in situ* by immunofluorescent imaging for HLA-ABC (Figure S1G); second, specificity was confirmed by inoculating SCID mice with GFP (green)-labeled PCa cells and recovering the DTCs from the marrow using APC (red)-labeled anti-HLA-ABC antibodies. As predicted, the cells isolated with the anti-HLA-ABC antibodies expressed GFP (Figure S1H–S1I). These data confirmed that HLA-ABC is a suitable marker for capturing human disseminated prostate cancer cells from marrow in animal models.

Using this approach, we compared the relative numbers of CSCs *in vitro* with CSCs *in vivo*. As predicted, the frequency of CSCs *in vitro* was extremely low (Figures 1C and S2A). Interestingly, 24 h after intracardiac injection, the CSC population of DTCs isolated from mouse marrow was more than 20% of total DTCs (Figures 1C and S2B); a significant increase within a short time period. The expansion of the CSCs was observed even following intracardiac injection of a non- CSC (CD133^−^/CD44^−^) (Figures 1D and S3A–S3B). Similar population shifts of non-CSC to CSC were observed *in vitro*, albeit not to the extent observed *in vivo* (Figures 1D and S3A–S3B), suggesting that the enrichment of CSCs is due to the conversion of non-CSCs into CSCs. Significantly, the enrichment of CSCs occurred only when prostate cancer cells spread to the marrow, not the lung or spleen, suggesting organ specificity (Figure 1E).

### CSC enrichment in marrow is not due to selection and is not an acute response

**Figure 2:**
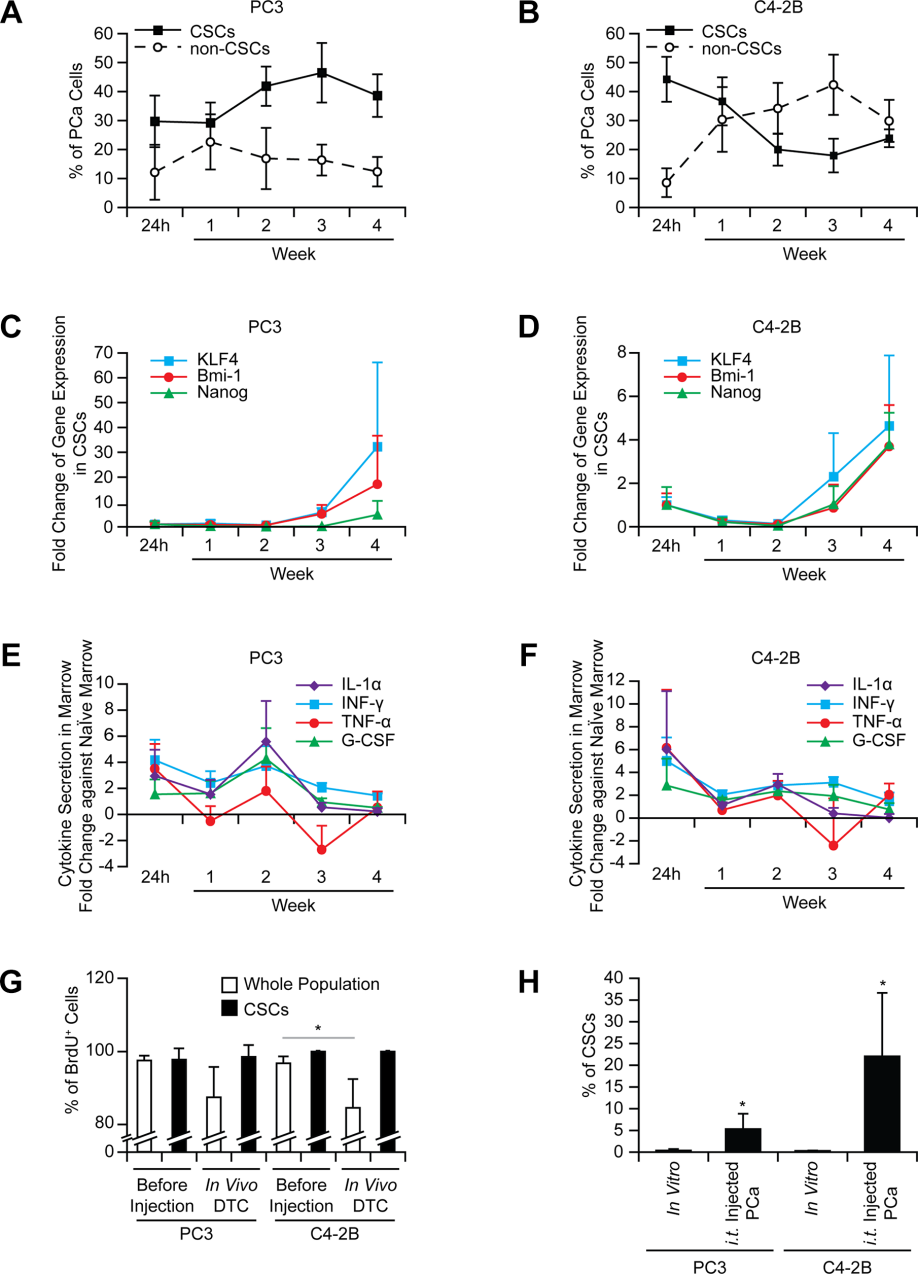
The increase in the CSCs in marrow is not an acute phase response and not due to selection The CSCs in prostate cancer recovered from bone marrow ((**A**) PC3 and (**B**) C4-2B), mRNA expression in prostate cancer cells ((**C**) PC3 and (**D**) C4- 2B), and secretion of inflammatory cytokines in marrow ((**E**) PC3 and (**F**) C4-2B) analyzed over time (*n* = 5 per week). (**G**) Intracardiac injection of BrdU stained prostate cancer was performed (*n* = 5). After 24 h injection, BrdU levels in DTCs were examined in the CSC vs. whole populations. *p < 0.05 (Student's *t*-test). (**H**) Prostate cancer was inoculated intratibially, and evaluated for CSCs after 24 h (*n* = 5). *p < 0.05 vs. *in vitro* cultured prostate cancer (Student's *t*-test).

To exclude the possibility that the increase in the CSCs is not merely an acute response to the marrow environment, cells were recovered from marrow over time. The conversion to the CSCs *in vivo* was maintained over 4 weeks (Figure 2A–2B), and the expression of stem cell-related genes, KLF4, Bmi-1, and Nanog, increased in the CSCs (Figure 2C–2D). Post intracardiac injection, the presence of DTCs in marrow initially alters cytokine production, but cytokine levels return to basal levels quickly, suggesting these proteins may not play a major role in the CSC conversion (Figure 2E–2F).

We next dissected the mechanisms involved in the conversion to the CSCs. Rapid proliferation was one potential mechanism to account for a larger CSC population. To assess this possibility, prostate cancer cells were stained with BrdU prior to injection. The CSCs recovered from marrow (24 h after injection) retained almost 100% of the BrdU detected before injection, while the population as a whole demonstrated the reductions in BrdU retention (Figure 2G), suggesting that non-CSC populations had undergone replication.

A second possibility worthy of consideration is that of selective CSC homing to marrow. To address this possibility, direct intraskeletal injections of prostate cancer were performed. The increase in CSCs was the same as that seen in intracardiac injections (Figure 2H), suggesting that the homing process does not account for the changes in population frequency. Next, to evaluate the possibility of a specific CSC survival advantage in circulation, a microfluidic device was fabricated (Figure S4A), which exposed cells to sheer stresses comparable to those present in the circulation. To take into account the additional effects of blood cells and serum on survival, prostate cancer cells were incubated with mouse blood. Based upon the results (Figure S4B), selective survival of the CSCs in circulation was an unlikely mechanism to account for the increase in the CSCs.

### The CSCs possess stem cell-like properties

**Figure 3:**
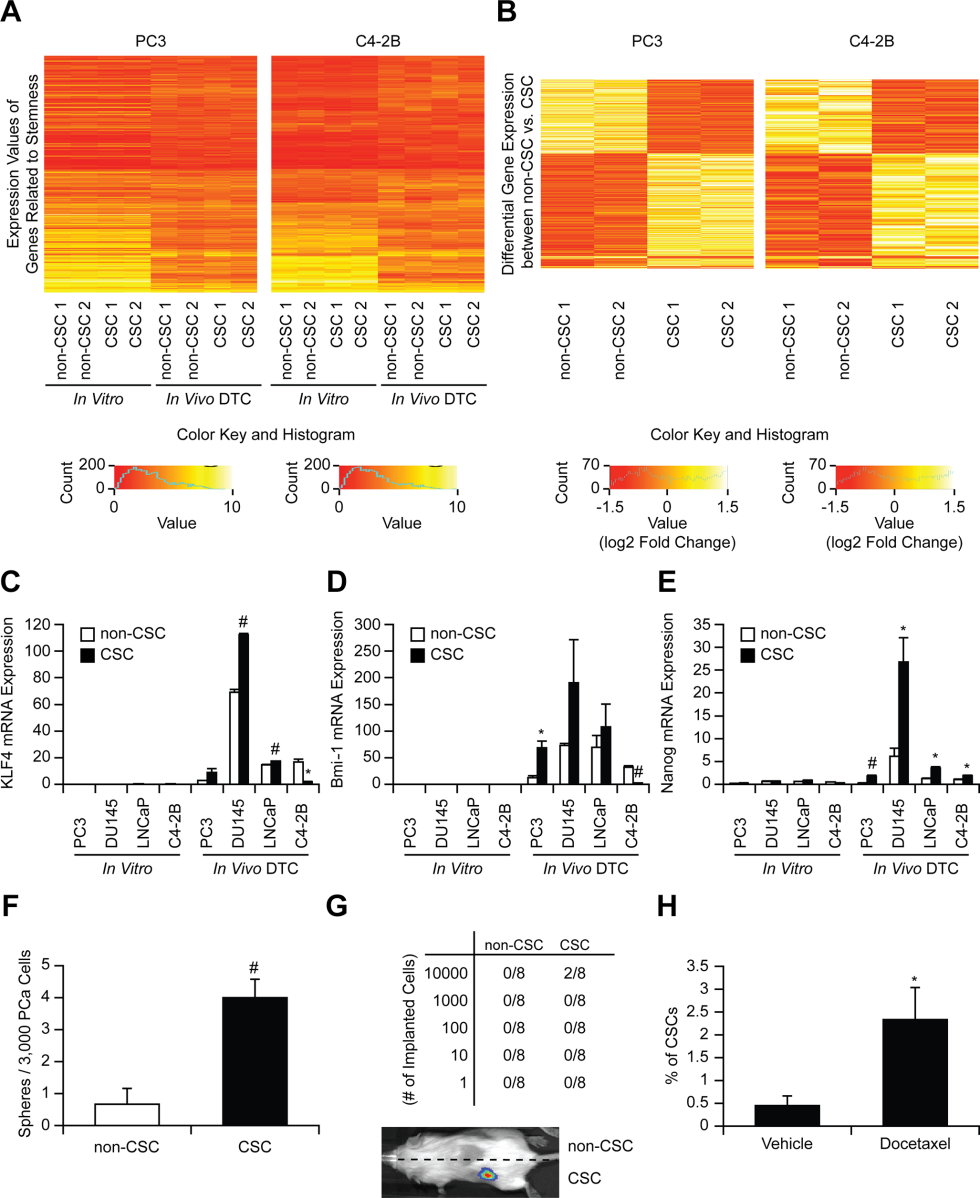
Stem-like properties of CSCs (**A**) Heatmap of stem cell related genes comparing CSC and non-CSC (*in vitro* and *in vivo*). (**B**) Heatmap of 619 differentially expressed genes in CSCs and non-CSCs (Wilcoxon Rank Sum *p* < 0.05, cells were obtained from 5 animals / experiments). mRNA expression of (**C**) KLF4, (**D**) Bmi-1, and (**E**) Nanog in CSC and non-CSC (*in vitro* and *in vivo*). *p < 0.05 and ^#^*p* < 0.01 vs. non-CSC (Kruskal-Wallis test). (**F**) *In vitro* sphere formation assays, (**G**) *In vivo* serial dilution stem cell assays (numbers correspond to tumors/implant), and (**H**) Chemo-resistance assays after docetaxel treatment (% of CSCs with/without docetaxel treatment). *p < 0.05 and ^#^*p* < 0.01 vs. vehicle treatment (Student's *t*-test).

We next validated that the CSCs examined in this study exhibit a stem cell-like phenotype. Microarray analyses revealed that *in vivo* DTCs displayed different stem like properties (genes were selected from the GO database using the GO term “stem cell”) than cells *in vitro* (expression values) (Figure 3A and Tables S1–S2). However, there were no differences in the gene expression of aldehyde dehydrogenase 1A2 (ALDH1A2) related to ALDH activity (one of the markers for CSCs including prostate cancer [17]) between CSCs and non-CSCs that are used in this study (Figure 3A and Tables S1– S2). Interestingly, 619 genes (including EpCAM) were differentially expressed (Wilcoxon rank sum *p*-value < 0.05) between CSC and non-CSC obtained from murine marrow (Figures 3B and S5A, and Tables S3), and in particular gene sets associated with stem cell activities were enriched in *in vivo* CSCs including Nuclear Receptors in Lipid Metabolism and Toxicity, Ca++/Calmodulin-dependent Protein Kinase Activation, and Cell Cycle: G2/M Checkpoints (Figure S5B). These global changes in gene expression suggest that the marrow niche plays a significant role in activating CSC programs. These differences were further defined by *Q*RT-PCR. The levels of mRNA expression for KLF 4, Bmi-1, and Nanog were dramatically increased overall in CSCs recovered from marrow verses *in vitro* (Figure 3C–3E). The microarray and PCR data further confirmed that CSCs obtained from murine marrow using HLA-ABC are human disseminated prostate cancer cells. In spite of their rarity, the CSCs isolated from marrow, when placed into culture, formed sphere-like structures (not shown). Additionally, the CSCs increased tumorigenic abilities both *in vitro* (Figure 3F) and *in vivo* (Figure 3G), and resistance to chemotherapy (Figure 3H), as expected for non-proliferating stem-like cells.

### Molecular mechanisms used by the osteoblastic niche to convert DTCs to CSCs

**Figure 4:**
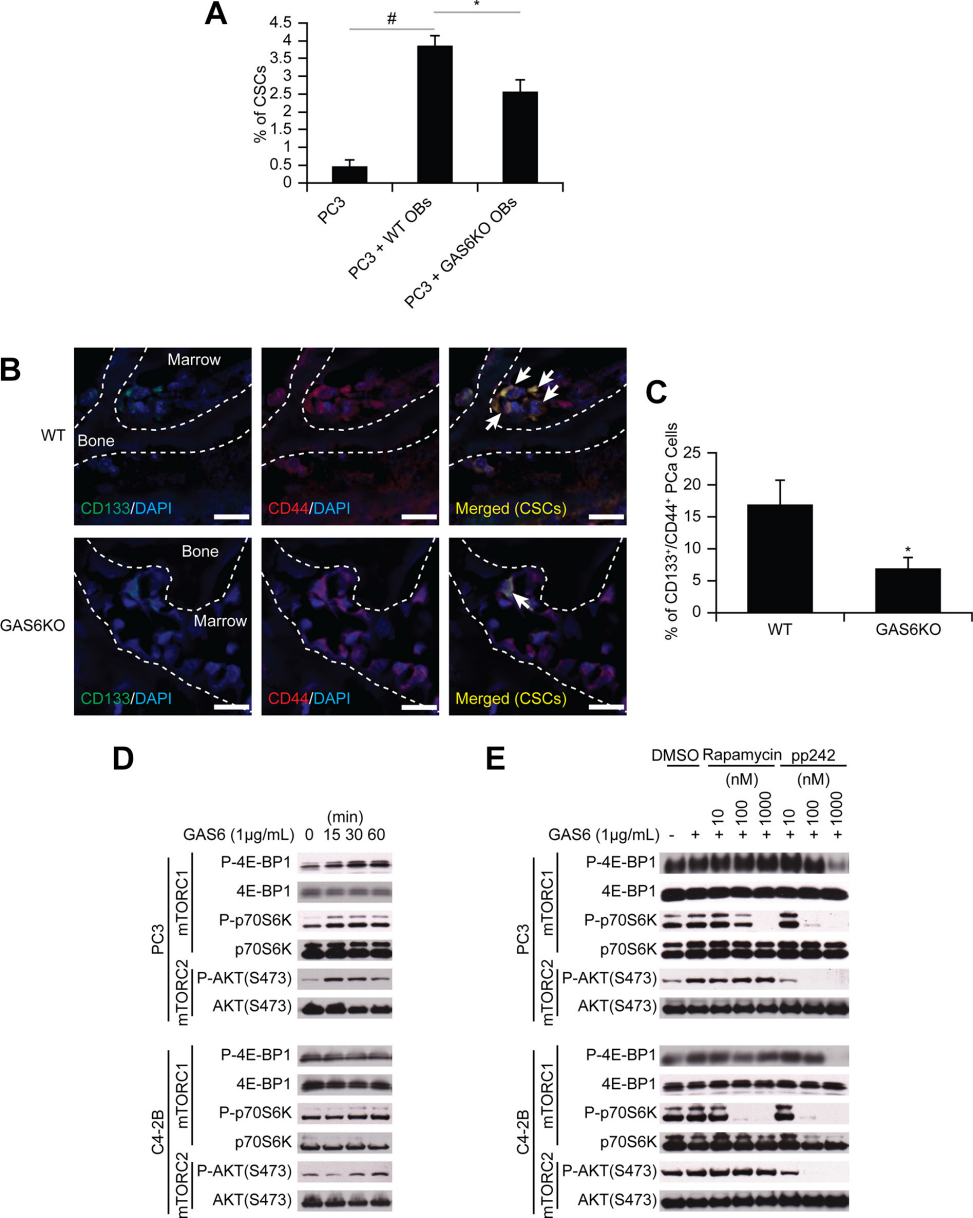
GAS6 expressed by the osteoblastic niche controls the conversion of DTCs to CSCs through mTOR signaling (**A**) The % of CSCs by flow cytometry in PC3 cells co-cultured with osteoblasts from *GAS6*^+/+^ (WT) and *GAS6*^−/−^ (GAS6KO) mice (Student's *t*-test). (**B**) PC3 cells (1 × 10^4^ cells per 10 μL) were placed directly into vertebral bodies (vossicles) derived from WT and GAS6KO mice and transplanted into immunodeficient mice (*n* = 6). At 1 month, the vossicles were dissected, and the expression of CSCs by prostate cancer was evaluated by immunohistochemistry. (60× Zoom 2.5, Bar = 20 μm). Arrows denote CSCs. (**C**) Quantitative analysis of CSC staining in Figure 4B. The CD133^+^CD44^+^ cells within six randomly selected representative images per group were counted, and then normalized with total numbers of cells. *p < 0.05 vs. CSCs in WT vossicle (Student's *t*-test). (**D**) Activation of mTOR signaling with GAS6 treatment in prostate cancer. (**E**) Activation of mTOR signaling with GAS6 treatment in prostate cancer in the presence/absence of mTOR inhibitors (Rapamycin, 13346, Cayman Chemical; and pp242, 13643, Cayman Chemical).

To identify the molecular mechanisms that regulate the shift of non-CSCs to CSCs in marrow, we studied co-cultures of prostate cancer and osteoblasts as DTCs compete for the occupancy of the osteoblastic niche [2], although certainly other components of the HSC niche are also involved. Under conditions of direct cell-to-cell contact, a significant shift of non- CSC to CSCs was observed (Figure 4A). Since GAS6 expressed by osteoblasts influences the proliferation of prostate cancer [12], and our microarray data suggests increased expression of Mer (one of the receptors for GAS6) in CSCs (Figure S5A), we asked whether GAS6 influences the conversion of non-CSCs to CSCs. When prostate cancer cells were co-cultured with osteoblasts isolated from GAS6-null mice, the conversion to CSCs was significantly, although not completely, diminished (Figure 4A). Moreover, when prostate cancer cells were injected into wild-type or GAS6-null skeletal tissue, significantly greater CSCs were identified near the endosteal surfaces of GAS6 expressing tissues compared to tissues lacking GAS6 (Figure 4B–4C). Next, we explored the downstream signaling activated by GAS6 that is involved in the expression of the CSC phenotype. Since the mTOR has been demonstrated to play a pivotal role in maintaining both a CSC phenotype and an HSC phenotype in the niche [18, 19], we explored the possibility that GAS6 activation of mTOR may represent a critical switch regulating the CSC phenotype. We found that GAS6 triggered mTOR signaling in prostate cancer, with increases seen in both mTORC1 and mTORC2 (Figures 4D and S6A–S6B), and these were diminished by the mTORC1 inhibitor rapamycin and the dual mTORC1/2 inhibitor pp242 (Figures 4E, S6C–S6D, and S7A–S7B).

**Figure 5:**
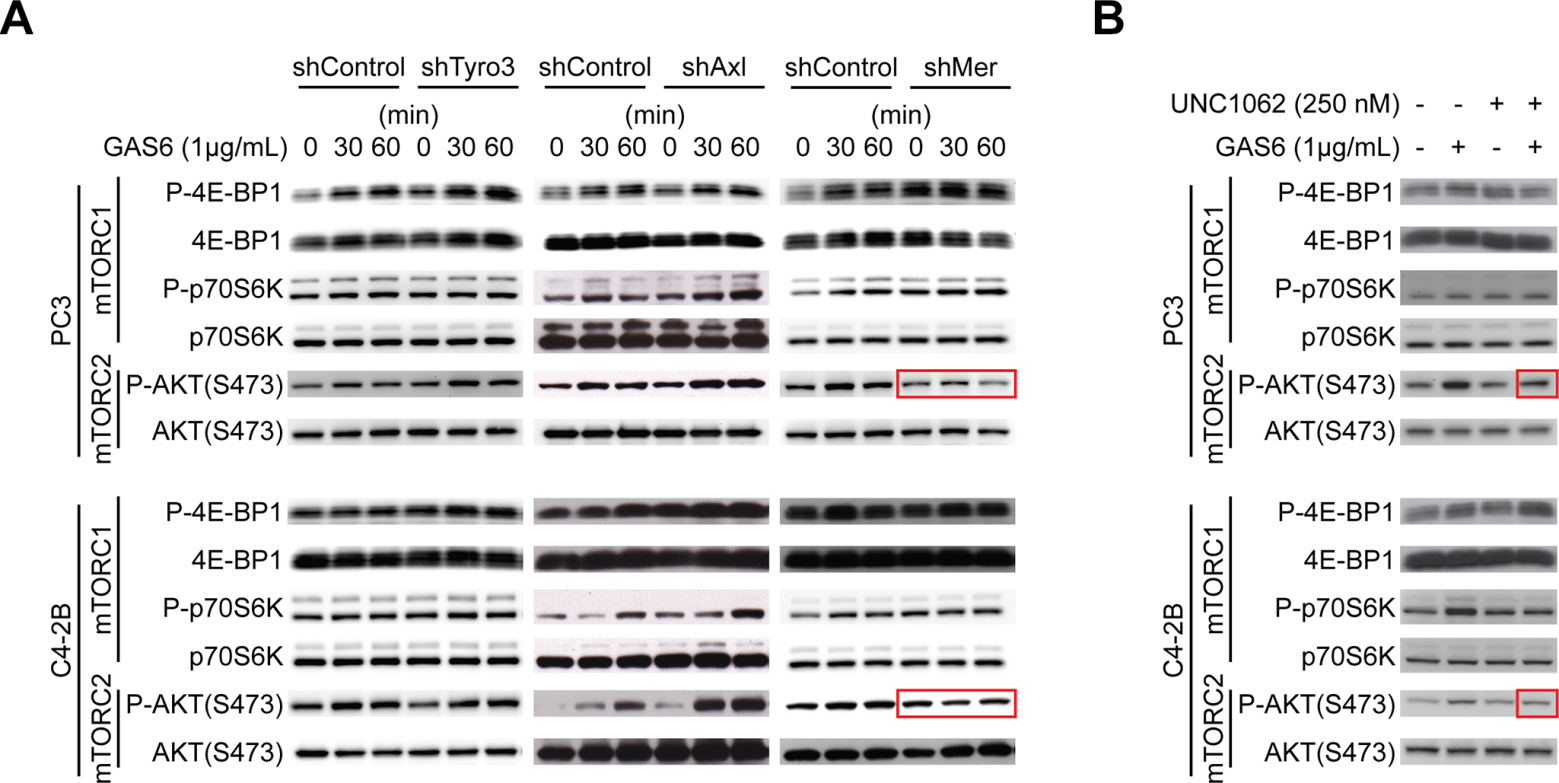
GAS6 activates mTOR signaling through Mer (**A**) Activation of mTOR signaling with GAS6 treatment in prostate cancer with TAM (Tyro3, Axl, Mer) receptors knocked down. (**B**) Activation of mTOR signaling with GAS6 treatment in prostate cancer in the presence/absence of Mer inhibitor (UNC1062).

**Figure 6:**
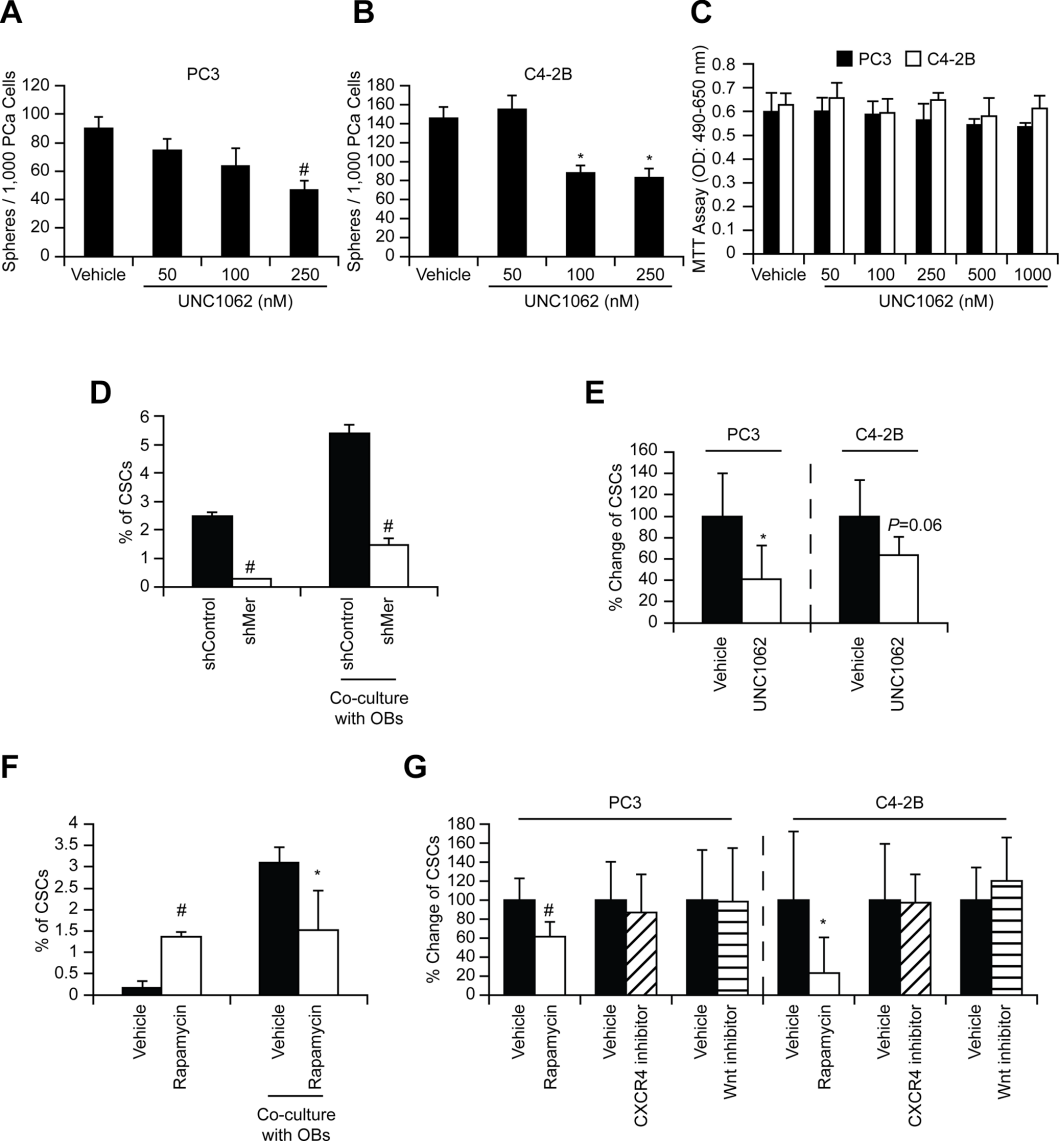
GAS6 activates mTOR signaling involved in the conversion of DTCs to CSCs through Mer *In vitro* sphere formation assays ((**A**) PC3 and (**B**) C4-2B)) and (**C**) MTT cell viability assays with/without Mer inhibitor (UNC1062). Significance vs. vehicle treatment (Student's *t*-test). (**D**) PC3 cells (shControl and shMer) were co-cultured with osteoblasts and 48 h later the CSCs in prostate cancer were measured by flow cytometry. Significance vs. shControl (Student's *t*-test). (**E**) Prostate cancer cells, pre-incubated (24 h) with UNC1062 (250 nM), were inoculated intracardially and 24 h later the CSCs in disseminated prostate cancer obtained from the bone marrow of mice inoculated with prostate cancer cells were measured by flow cytometry (*n* = 5). The % of CSCs population in the marrow was normalized to the vehicle control group that equals 100%, and presened as % change. Significance vs. vehicle treated prostate cancer (Student's *t*-test). (**F**) PC3 cells, pre-incubated (1 h) with Rapamycin (10 nM), were co-cultured with osteoblasts and 48 h later the CSCs in prostate cancer were measured by flow cytometry. (**G**) Prostate cancer cells, pre-incubated (1 h) with Rapamycin (13346, Cayman Chemical, 10 nM), CXCR4 inhibitor (AMD3100, A-5602, Sigma-Aldrich, 10 nM), or Wnt inhibitor (IWR-1-endo, 13659, Cayman Chemical, 10 μM), were inoculated intracardially and 24 h later the CSCs in disseminated prostate cancer were measured by flow cytometry (*n* = 5). Significance vs. vehicle treated prostate cancer (Student's *t*-test). *p < 0.05 and ^#^*p* < 0.01.

To identify which of the GAS6 receptors (Tyro3, Axl, and Mer) is responsible for mTOR activation, targeted deletion of each of the three receptors was performed (Figure S8A–S8B). When Mer was reduced in prostate cancer via shRNA (Figures 5A, S9, and S10), or a Mer inhibitor was employed (Figures 5B and S11), mTORC2 signaling activated by GAS6 was decreased, which was not observed with reduced Tyro3 and Axl expression. Activation of mTORC1 in shMer cells was also reduced compared to shTyro3 and shAxl cells (Figure 5A). These results suggest that the increase in the CSCs is mediated through the GAS6/Mer axis. To test the roles of PTEN in this system, DU145 was used, since DU145 expresses PTEN, while PC3 and C4-2B are PTEN deficient [15, 20]. As in PC3 and C4-2B, Mer inhibitor blocked mTORC2 activation in DU145 (Figures S12A and S13A). However, when shMer DU145 cells were treated with GAS6, mTORC2 signaling was unaffected (Figures S12B and S13B), which we attributed to the high residual levels of MER, when compared to PC3 and C4- 2B (Figure S14). These data suggest that PTEN status is likely involved in mTOR activation through GAS6/Mer, but PTEN is unable to prevent the conversion to CSCs in DU145 (Figure 1C). Moreover, the Mer inhibitor prevents sphere-forming ability of prostate cancer (Figures 6A–6B and S15), while it did not affect cell viability (Figure 6C). Additionally, the conversion to CSC by direct contact with osteoblasts was inhibited both *in vitro* (Figure 6D, shControl vs. shMer) and *in vivo* (Figure 6E, Vehicle vs. Mer inhibitor), when the Mer in prostate cancer was inhibited. Similarly, fewer CSCs were recovered from the marrow of animals inoculated with shControl vs. shMer, although the difference did not reach statistical significance (Figure S16). Importantly, the increase in CSCs was also inhibited both *in vitro* (Figure 6F) and *in vivo* (Figure 6G), when the mTOR signaling pathway in prostate cancer was blocked. However, the conversion to CSCs was not seen when Wnt or CXCR4 pathways were blocked, which are also believed to regulate stemness (Figure 6G).

## DISCUSSION

Local or distant tumor recurrence, in spite of early therapeutic interventions, suggests that dissemination of tumor cells occurs very early in cancer development. In prostate cancer, epithelial-like cells can be isolated from bone marrow in 72% of prostate cancer patients when diagnosed, and even in 8.8% of healthy controls [7]. However, whether those cells are normal epithelial cells or DTCs derived from an unknown primary prostate cancer remains unclear. In addition, DTCs are believed to become occult in bone marrow, as prostate-specific antigen (PSA), which correlates with tumor volume, persists at undetectable levels years after radical prostatectomy [21], and yet metastatic tumors can occur long after initial treatment. The development of new therapeutic regimens to augment existing chemotherapies, surgery and radiation are essential if we hope to establish long-term treatments for prostate cancer. However, our lack of understanding of the biology of DTCs remains a major stumbling block in the process.

In this study, we first explored that disseminated prostate cancer recovered from marrow were highly enriched for CSCs. We elected CD133^+^/CD44^+^ as a CSC surface marker combination, since this is widely used to isolate prostate CSCs [2, 15, 22–24]. However, there is no difference in the ALDH1A2 gene expression between CSCs and non-CSCs (Figure 3A and Tables S1–S2), suggesting that there may be other population of CSCs do not express the selected CSC surface marker combination. Further studies are clearly needed as no consensus has yet been reached regarding specific markers for prostate CSC.

**Figure 7:**
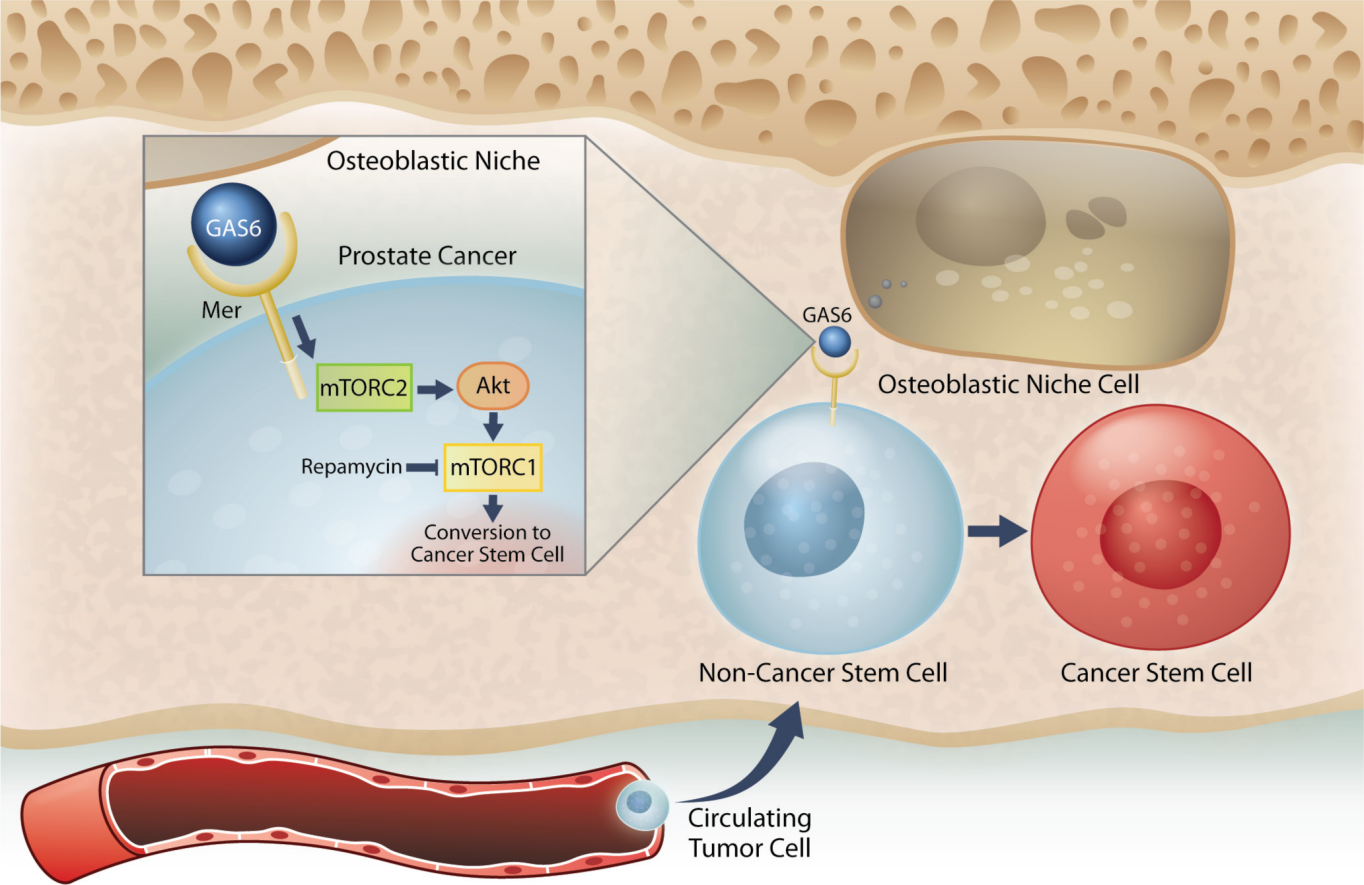
Model system for the induction of a CSC phenotype in marrow by the HSC niche Prostate cancer cells preferably spread to the bone and survive within the marrow microenvironment for a long period of time. However, the mechanisms underlying the survival of these disseminated tumor cells (DTCs) remain unclear. Our previous work revealed that prostate cancer DTCs target the osteoblastic hematopoietic stem cell (HSC) niche, and that these DTCs parasitize the niche to survive there. The major function of the niche is maintaining the stem cell phenotype. This study demonstrated that the conversion of cancer cells to stem-like cancer cells (CSC) occurs when DTCs directly contact the osteoblastic niche. GAS6 expressed by the osteoblastic niche activates mTOR signaling in the prostate cancer DTCs through the Mer receptor, contributing to the conversion to CSCs. Furthermore, our data suggests that these activations uniquely progress first through mTORC2 and then mTORC1, which can be blocked by rapamycin. Therefore, targeting mTOR signaling in DTCs could be a promising therapy for bone metastatic disease.

Next, when uniformly non-CSCs were inoculated into the mice, a conversion to CSCs was observed in prostate cancer cells recovered from marrow. Importantly, this conversion was seen only in the prostate cancer cells localized to the marrow, but not lung or spleen. Further, this enrichment of CSC population was not due to the effects of cell proliferation, survival within the circulation, or homing. Additionally, the CSCs exhibit expression of the self-renewal genes KLF4, Bmi-1, and Nanog, and have the ability to form sphere-like structures *in vitro*, and tumor *in vivo* although not robust. Under conditions of direct cell-to-cell contact between prostate cancer cells and osteoblasts, a significant shift of non-CSCs to CSCs was observed. With further analysis, we found that within the marrow the osteoblastic niche controls conversion of disseminated prostate cancer cells to CSCs through the GAS6/Mer/mTOR pathway (Figure 7). Collectively, our findings suggest that the bone marrow niche plays an important role in the accumulation of self-renewing prostate cancer cells in the marrow, which further indicates that DTCs may be capable of serving as metastatic seeds for bone tumors, and that this model and strategy may be useful in further exploration of the nature and phenotype of DTCs. Understanding how the niche regulates the conversion of DTCs to CSCs will be instrumental for the development of therapeutics specifically targeting early dissemination of prostate cancer to the bone, and for understanding how metastatic growth is regulated.

We previously identified that the osteoblastic niche regulates the proliferation of prostate cancer via the GAS6 pathway [12, 14, 25]. The growth of prostate cancer in the marrow depends on the levels of GAS6 that bones express [25]. That is, human prostate cancer xenografts grow rapidly in the osseous environment expressing less GAS6, compared to high GAS6-expressing bones [25]. Prostate cancer growth also depends on the expression levels of GAS6 receptors on prostate cancer cells [14]. Dormant prostate cancer expresses higher Axl (Axl > Tyro3), but cells expressing Tyro3 (Tyro3 > Axl) grow rapidly [14]. Consistently, when prostate cancer reaches the bone, Axl expression in prostate cancer and GAS6 expression in osteoblasts both increase simultaneously [12]. These findings suggest that GAS6 is important for the proliferation of disseminated prostate cancer. However, in the present study we discovered a new, important role for GAS6 in the progression of prostate cancer in the marrow: GAS6 expressed by osteoblasts converts disseminated prostate cancer to a stem-like phenotype through its receptor, Mer. This discrepancy is very similar to the effects of TGF-ß and BMP signaling on bone metastasis. Whether these factors influence CSC phenotype and tumor dormancy in bone metastatic diseases is highly dependent on cell type and/or extracellular microenvironment [26–31]. Likewise, we speculated that the effects of GAS6 on the progression of DTCs are dependent on the expression levels of its receptor. Since the interactions between ligands and receptors are complex, further study is needed to determine the exact role of each GAS6 receptor in the fate of disseminated prostate cancer.

Metastasis remains a life-threatening complication of solid tumors. Once the tumor cells spread to distant organs such as bone, survival rates of cancer patients decline drastically. Despite the controversies over the CSC hypothesis [32, 33], consensus has been reached that the most effective therapies will also need to target chemo-resistant CSCs. Our data suggests that the niche plays a central role in activating in the conversion of disseminated prostate cancer to CSCs. Thus, it appears that targeting only CSCs could be of limited therapeutic value, since stem cell programs can become activated in DTCs. Similar to our findings, other recent reports have demonstrated that conversions between somatic cells and stem cells [15, 34], somatic cells and CSCs [35], and non-CSCs and CSCs [36] are possible.

Considering that CSCs likely represent a heterogeneous population with a wide spectrum of epithelial and mesenchymal characteristics (e.g. EMT CSCs and MET CSCs) [37], these findings further suggest an addendum to the “seed and soil” hypothesis first proposed by Stephan Paget in 1889 [38]. In this theory, the “seed” (tumor cells) favorably metastasize to the “soil” (their specific microenvironment). However, our data infers that the “soil” (the niche) is also a major driver of the creation/maintenance of the “seed” (CSCs). While further studies in different cancer types are clearly needed, the identification of alternative mechanisms whereby CSCs are generated expands our knowledge and understanding as to how tumors are propagated, and sets the stage for future therapeutic developments to target prostate cancer bone metastases.

## MATERIALS AND METHODS

### Tissue microarray and immunostaining

Human prostate adenocarcinoma tissue microarray (TMA) was obtained from The Tissue Core of the University of Michigan Comprehensive Cancer Center. Tumors were examined to identify areas of benign prostatic hyperplasia, prostatic intraepithelial neoplasia, primary prostate cancer, and bone metastatic prostate cancer. TMA slides were de-waxed with xylenes and re-hydrated with 100%, 90%, 70%, and 50% ethanol. The slides were then permeabilized with PBST (1/500 Triton X-100 in PBS), blocked with Image-iT FX signal enhancer (136933, Life Technology, Carlsbad, CA) for 30 min, and incubated for 2 h at room temperature with anti CD44 (ab51037, Abcam, Cambridge, MA, pre-stained with Zenon Alexa Fluor 488, Z-25302, Invitrogen, San Diego, CA) and anti CD133 (130-090-422, Miltenyi Biotec, San Diego, CA, pre-stained with Zenon Alexa Fluor 555, Z-25005) antibodies. After washing with PBS, these slides were mounted with ProLong Gold antifade reagent with DAPI (P-36931, Life Technology). Images were taken with Olympus FV-500 confocal microscope (Olympus, Center Valley, PA). The CD133^+^CD44^+^ area was measured in randomly selected 5–16 different fields of the each group (BPH, PIN, Primary prostate cancer, or Bone Mets).

### Cell culture

Human prostate cancer cell lines (PC3, CRL-1435; DU145, HTB-81; LNCaP, CRL-1740) were obtained from the American Type Culture Collection (Rockville, MD). The metastatic subclone of LNCaP, C4-2B, was a subline derived from bone metastasis of LNCaP bearing-mouse. Luciferase-expressing prostate cancer cells were established by lentiviral transduction. Murine osteoblast cells were established as previously reported [2]. All prostate cancer cell lines were routinely grown in RPMI 1640 (11875–093, Life Technologies), and murine osteoblast cells were grown in α-MEM (12571–063, Life Technologies). Cultures were supplemented with 10% (v/v) fetal bovine serum (900–108, GEMINI Bio-Products, Sacramento, CA), 1% (v/v) penicillin-streptomycin (15140–122, Life Technologies) and maintained at 37°C, 5% CO_2_, and 100% humidity. These cells were certified by DDC Medical.

### Flow cytometry

Cells were stained with FITC- or APC-anti-HLA-ABC antibody (clone W6/32: 311404 (FITC), 311410 (APC), BioLegend, San Diego, CA), PE-cytokeratin antibody (347204, Becton Dickinson, Franklin Lakes, NJ), PE-EpCAM antibody (130-091-253, Miltenyi Biotec), PE-anti-CD133 antibody (130-080-801, Miltenyi Biotec), APC-anti-CD44 antibody (559942, BD Biosciences, San Diego, CA), or isotype-matched IgG control for 20 min at 4°C. Flow cytometric analyses were performed in a FACSAria II High-Speed Cell Sorter (Becton Dickinson).

### *In vivo* isolation of disseminated prostate cancer

Prostate cancer cells (1 × 10^6^ cells) were injected into male CB.17. SCID mice (4–6 weeks of age: Charles River, Wilmington, MA) by intracardiac or intratibial injection. Bone marrow cells were flushed from femurs and tibias 24 h later. Single cell preparations were incubated first with a Lineage Cell Depletion Kit magnetic labeling system with biotinylated anti-Lineage (CD5, CD45R (B220), CD11b, Gr-1 (Ly-6G/C), and Ter-119) antibody cocktail (130-092-613, Miltenyi Biotec) and anti-Biotin MicroBeads (130-090-485, Miltenyi Biotec), and then enriched for murine Lineage negative population using an AutoMACS machine (Miltenyi Biotec). The enriched cells were incubated with a FITC- anti-HLA-ABC antibody, PE-anti-CD133 antibody, and APC-anti-CD44 antibody for another 20 min at 4°C. Thereafter, the CD133^+^/CD44^+^ and CD133^−^/CD44^−^ fractions were sorted with a FACSAria II Cell Sorter by gating on HLA-ABC positive cells. All experimental procedures were approved by the University of Michigan Committee for the Use and Care of Animals.

### Cytokine arrays

Bone marrow extracellular fluid was obtained by flushing femur and tibia of tumor-bearing mice with 500 μL ice-cold phosphate-buffered saline containing protease inhibitor cocktail (P8340, Sigma-Aldrich, St. Louis, MO), and the supernatant was harvested by centrifugation at 400 *g* for 5 min. Cytokine levels in the marrow fluids were analyzed by antibody sandwich enzyme-linked immunosorbent assay (Human Inflammatory Cytokines Multi-Analyte ELISArray Kit, MEH004A, QIAGEN, Valencia, CA), according to the manufacturer's protocol. The marrow fluids obtained from non-tumor bearing mice were used as controls. Cytokine levels were normalized to total protein.

### *In vivo* cell proliferation assays

Prostate cancer cells were stained with BrdU Labeling Reagent (00-0103, Life Technologies) according to the manufacturer's protocol. These BrdU-stained prostate cancer cells were then injected into male SCID mice by intracardiac injection. Bone marrow cells were flushed from the femurs and tibias 24 h later, and resulting cells were incubated with a FITC-anti-HLA-ABC antibody and anti-BrdU antibody (ab8039, Abcam), followed by PE-Cy7-secondary antibody (406614, BioLegend). Retention of BrdU in prostate cancer cells was analyzed by gating on HLA-ABC positive cells with a FACSAria II Cell Sorter.

### Microarray analyses

The CD133^+^/CD44^+^ fraction and CD133^−^/CD44^−^ fraction were sorted with a FACSAria II Cell Sorter from *in vitro* cultured prostate cancer cells and *in vivo* disseminated prostate cancer cells obtained from bone marrow of SCID mice innoculated with prostate cancer cells (24 h) by intracadiac injection. The extractions and purifications of RNA from the resulting cells were performed using RNeasy Plus Micro RNA kit, which allowed RNA isolation and DNAse treatment from small cell numbers. RNA samples were submitted to the University of Michigan Sequencing core for quality evaluation followed by microarray analysis using an Affymetrix platform (Affymetrix, Santa Clara, CA). Data normalization and analysis was done using R and Bioconductor. The affymetrix CEL files were pre-processed and quantile normalized using Robust Multiarray Average (RMA). The probesets were annotated and mapped to gene symbols using ‘hugene21sttranscriptcluster.db’ on Bioconductor. In the case of multiple probes mapping to one gene symbol, the probe with the maximum mean was selected (WCGNA, R package). The Wilcoxon Rank Sum test was used to determine the differentially expressed genes between CSCs and non-CSCs. Hierarchical clustering of the samples was done using Euclidean distance and average linkage. To identify gene sets enriched in CSCs, GSEA at Broad (Broad Institute, MIT; http://www.broad.mit.edu/gsea/index.jsp) and Biocarta genesets were used. The signal to noise ratio was used as the gene list ordering mode.

### RNA extraction and real-time RT-PCR

Total RNA was isolated using RNeasy Mini Kit (74106, QIAGEN). First-strand cDNA was synthesized in a 20 μL reaction volume using 0.4 μg of total RNA. RT products were analyzed by real-time RT-PCR in TaqMan^®^ Gene Expression Assays (KLF4, Hs00358836_m1; Bmi1, Hs00995536_m1; Nanog, Hs04399610_g1; Tyro3, Hs00170723_m1; Axl, Hs01064444_m1; Mer, Hs01031973_m1; β-actin, Hs01060665_g1, Applied Biosystems, Foster City, CA). The 2nd step PCR reactions were run for 40 cycles (95°C for 15 sec and 60°C 1 min) after an initial single cycle of 50°C for 2 min and 95°C for 10 min. The PCR product was detected as an increase in fluorescence using an ABI PRISM 7700 instrument (Applied Biosystems). RNA quantity (C_R_) was normalized to the housekeeping gene β-Actin control, using the formula C_R_ = 2^(40-Ct of sample)-(40-Ct of control)^. The threshold cycle (Ct) is the cycle at which a significant increase in fluorescence occurs.

### *In vitro* prostatosphere formation assays

Prostatosphere formation assays were performed using a slight modification of previously described techniques [39, 40]. Cells were plated in DMEM F-12 (11320-033, Life Technologies) containing 10 ng/mL bFGF (233-FB/CF, R&D Systems, Minneapolis, MN), 20 ng/mL EGF (236-EG, R&D Systems), 5 mg/mL insulin (3435, R&D Systems), and 0.4% (v/v) bovine serum albumin (BSA) (5217, R&D Systems) supplemented with 1% (v/v) knockout serum replacement (10828-028, Life Technologies) at 500-3,000 cells per well in 6-well ultra low attachment plates. In some case, the cultures were treated with the Mer inhibitor UNC1062 (AOB4488, AOBIOUS, Gloucester, MA). Prostatosphere formation (cell clusters of 10 cells or greater) was observed at 7–10 days under a light microscopy.

### *In vivo* serial dilution tumor-propagating assays

The CD133^+^/CD44^+^ fraction and CD133^−^/CD44^−^fraction were sorted from *in vitro* cultured luciferase expressing prostate cancer cells with a FACSAria II High-Speed Cell Sorter. The resulting cells were suspended in serum-free RPMI/cytokine reduced collagen gel mixture (1:1 volume) and then implanted subcutaneously into SCID mice (1 to 1 ×10^4^ viable cells). Tumor growth was tracked by bioluminescence imaging, performed as previously described [37], through the University of Michigan Small Animal Imaging Resource facility.

### *In vitro* chemoresistance assays

Prostate cancer cells were treated with/without docetaxel (1 μg/mL, 01885, Sigma-Aldrich) and the cultures were incubated at 37°C for 48 h. Thereafter, the CD133^+^/CD44^+^ fraction in resulting cells was analyzed with a FACSAria II Cell Sorter.

### *In vitro* co-culture

Prostate cancer cells were cultured on murine calvarial osteoblasts obtained from wild type or *GAS6*-deficient (GAS6^−/−^) mice for 48 h, and then the CD133^+^/CD44^+^ fraction was analyzed using a FACSAria II Cell Sorter by gating on HLA-ABC positive cells. The laboratory of Dr. Peter Carmeliet (University of Leuven, Leuven, Belgium) generated the *GAS6*^−/−^animals and graciously provided our laboratory with a pair of the homozygous *GAS6*^−/−^ mice for breeding.

### Vossicle transplant

Lumbar vertebrae were isolated from 4- to 7-day-old *GAS6*^+/+^ or *GAS6*^−/−^ mice. The vertebrae were sectioned into single vertebral bodies (a.k.a. vossicles). SCID mice were used as transplant recipients. Two vossicles per mouse were implanted into subcutaneous space as previously described [2]. Before implantation, PC3 cells were introduced into both vossicles (10,000 cells/10 μL of PBS). Mice were sacrificed at 3 weeks, and the vossicles prepared for immunohistochemistry.

### Immunohistochemistry

To detect human cells grown in mice, anti-human HLA-ABC antibody (311402, BioLegend) was conjugated using the Zenon Alexa Fluor 555 mouse IgG_2a_ labeling kit (Z-25105). To detect human CD133 antigen, purified mouse IgG1 antibody (Miltenyi Biotec) was conjugated using the Zenon Alexa Fluor 488 mouse IgG1 labeling kit (Z-25002). For detection of human CD44 antigen, the antibody (Abcam) was conjugated using the Zenon Alexa Fluor 555 Rabbit IgG labeling kit (Z-25305). 7 μm thick paraffin sections were generated, and antigen retrieval performed with a pepsin solution at 37°C for 15 min, followed by washing with PBT (PBS plus 0.2% Triton X-100) for 5 min at room temperature. Each section was blocked with Image-iT FX signal enhancer (Invitrogen) for 30 min before fluorescence-labeled primary antibodies were applied for 2 h at room temperature in the dark. Subsequently, the sections were washed twice by submersion in PBS for 10 min, subjected to post-stain fixation with 10% formalin (Sigma-Aldrich), and mounted with ProLong Gold anti-fade reagent with DAPI. Images were taken with Olympus FV-500 confocal microscope.

### Western blots

Prostate cancer cells were prepared in lysis buffer (CelLytic MT Mammalian Tissue Lysis Reagent, C3228, Sigma-Aldrich), and protein concentration was quantified using a DC Protein Assay Kit II (5000112, Bio-RAD, Hercules, CA). Cell extracts (30 μg of protein per lane) were loaded and separated on SDS-PAGE (4–20% Bis-Glycine gradient gels, EC6025BOX, Invitrogen) and transferred to a PVDF membrane. Membranes were incubated with 5% milk for 1 h and incubated with anti-Tyro3 (585S) -Axl (4977), -Mer (4319), -GAPDH (2118), -P-4E-BP1 (2855), -4E-PB1 (9644), -P-p70S6K (9234), -p70S6K (2708), -P-AKT(S473) (9271), or -AKT(S473) (9272) antibody (all primary antibodies were obtained from Cell Signaling Technology, Danvers, MA) overnight at 4°C. Primary antibody was used with 5% dry milk. Blots were incubated with peroxidase-coupled secondary antibodies (W4011, Promega, Madison, WI) for 1 h at a ratio of 1:3000. Protein expression was detected with SuperSignal West Pico Chemiluminescent Substrate (34080, Thermo Scientific, Rockford, IL). The densitometric analysis of the Western blot were performed with ImageJ software (version 1.50i; National Institutes of Health, Bethesda, MD).

### TAM receptors knockdown

Stable knockdowns of TAM receptors (Tyro3, Axl, Mer) in prostate cancer cells were generated by lentiviral infection. Lentiviruses were constructed at the Vector Core in University of Michigan (Ann Arbor, MI) using GIPZ Lentiviral shRNAmir vectors containing either TAM receptors (Tyro3, Axl, Mer) shRNA or nonsilencing (scrambled) shRNA (Open Biosystems, Lafayette, CO).

### Statistical analyses

Numerical data are expressed as mean ± standard deviation. Statistical analysis was performed by unpaired two-tailed Student's *t* test using the GraphPad Instat statistical program (GraphPad Software, San Diego, CA) with significance at *P* < 0.05. For the real-time RT-PCR assays, a Kruskal-Wallis test and Dunn's multiple comparisons tests were utilized with the level of significance set at *P* < 0.05.

## SUPPLEMENTARY MATERIALS FIGURES AND TABLES









## Abbreviations

CSCs: Cancer stem cells
HSCs: hematopoietic stem cell
DTCs: disseminated tumor cells
PSA: prostate-specific antigen
TMA: tissue microarray
BSA: bovine serum albumin
