# Correction: The marrow niche controls the cancer stem cell phenotype of disseminated prostate cancer

**DOI:** 10.18632/oncotarget.18369

**Published:** 2017-06-05

**Authors:** Yusuke Shiozawa, Janice E. Berry, Matthew R. Eber, Younghun Jung, Kenji Yumoto, Frank C. Cackowski, Hyeun Joong Yoon, Princy Parsana, Rohit Mehra, Jingcheng Wang, Samantha McGee, Eunsohl Lee, Sunitha Nagrath, Kenneth J. Pienta, Russell S. Taichman

**Present:** The original figure 7 misspelled the word 'rapamycin.

**Correct:** The proper spelling appears in the figure below. The authors sincerely apologize for this error.

Original article: Oncotarget. 2016; 7:41217-41232. doi: 10.18632/oncotarget.9251

**Figure 7:**
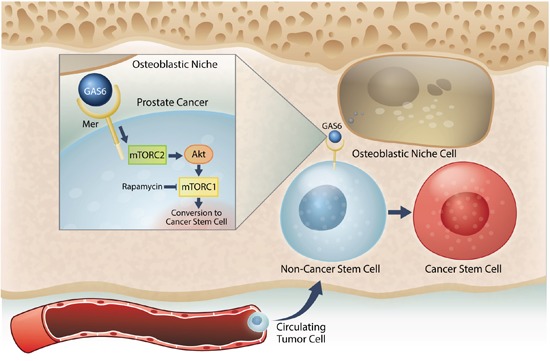
Model system for the induction of a CSC phenotype in marrow by the HSC niche Prostate cancer cells preferably spread to the bone and survive within the marrow microenvironment for a long period of time. However, the mechanisms underlying the survival of these disseminated tumor cells (DTCs) remain unclear. Our previous work revealed that prostate cancer DTCs target the osteoblastic hematopoietic stem cell (HSC) niche, and that these DTCs parasitize the niche to survive there. The major function of the niche is maintaining the stem cell phenotype. This study demonstrated that the conversion of cancer cells to stem-like cancer cells (CSC) occurs when DTCs directly contact the osteoblastic niche. GAS6 expressed by the osteoblastic niche activates mTOR signaling in the prostate cancer DTCs through the Mer receptor, contributing to the conversion to CSCs. Furthermore, our data suggests that these activations uniquely progress first through mTORC2 and then mTORC1, which can be blocked by rapamycin. Therefore, targeting mTOR signaling in DTCs could be a promising therapy for bone metastatic disease.

