# Supplementary material for: The marrow niche controls the cancer stem cell phenotype of disseminated prostate cancer
Source: Oncotarget. 2016 May 9;7(27):41217–32. doi: 10.18632/oncotarget.9251 (PMC5173053; doi:10.18632/oncotarget.9251)
Supplement: Supplementary file 1 [file oncotarget-07-41217-s001.pdf]

The marrow niche controls the cancer stem cell phenotype of disseminated prostate cancer

Supplementary Materials

A

% Positive of Cytokeratin, EpCAM, and HLA-ABC In PCa and Leukemia Cells (In Vitro)	PC3	DU145	LNCaP	C4-2B	RCH-ACV	697	Nalm6	RS4;11
Cytokeratin	4.07	5.47	8.90	18.23	0.59	0.07	0.28	0.07
EpCAM	94.47	61.63	99.67	99.50	0.63	0.06	0.14	0.05
HLA-ABC	99.91	99.98	99.42	98.69	88.79	98.44	48.57	0.22

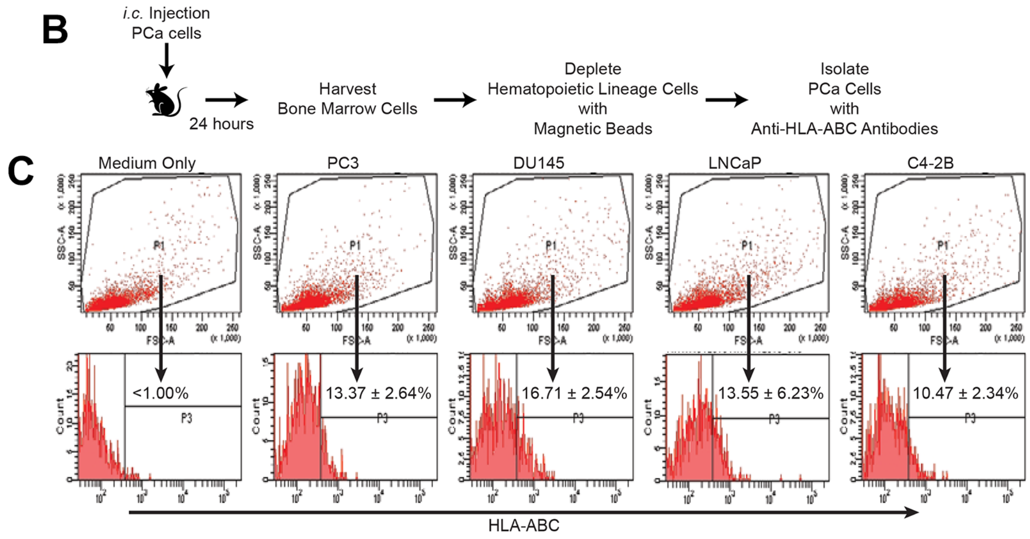

D

% Positive of Cytokeratin and EpCAM in HLA-ABC Positive PCa Cells (In Vivo)	PC3	DU145	LNCaP	C4-2B
Cytokeratin	23.07	23.73	24.60	37.90
EpCAM	45.77	38.60	31.03	40.13

E

% Positive of HLA-ABC in Cytokeratin Positive PCa Cells (In Vivo)	PC3	DU145	LNCaP	C4-2B
HLA-ABC	100.00	98.32	95.85	100.00

F

% Positive of HLA-ABC in EpCAM Positive PCa Cells (In Vivo)	PC3	DU145	LNCaP	C4-2B
HLA-ABC	88.08	83.48	85.70	85.51

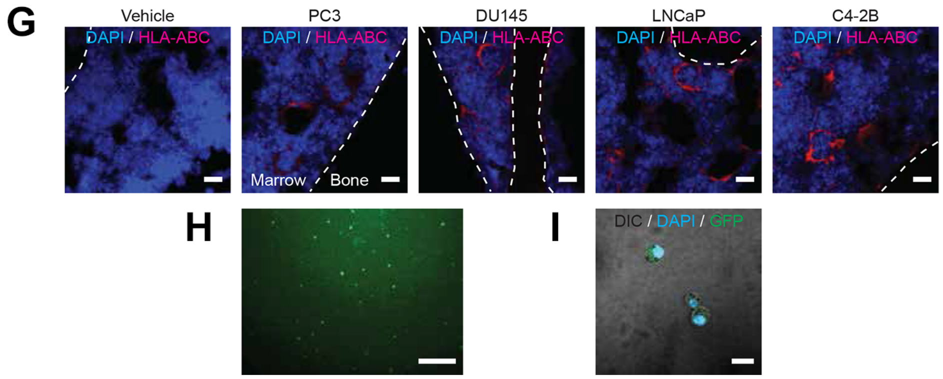

Supplementary Figure S1: Detection of DTCs in murine marrow with HLA-ABC. (**A**) Flow cytometric analyses for cytokeratin, EpCAM and HLA-ABC expression on prostate cancer and leukemia cell lines *in vitro*. (**B**) Experimental DTC model. (**C**) Flow cytometric measurement of human DTCs recovered from murine marrow using HLA-ABC antibodies. (**D**) Flow cytometric analyses of cytokeratin and EpCAM expression in HLA-ABC positive DTCs recovered from mice marrow. Flow cytometric analyses of HLA-ABC expression in (**E**) cytokeratin and (**F**) EpCAM positive DTCs recovered from mice marrow. (**G**) Representative HLA-ABC expression in bone marrow by immunofluorescent histology following establishment of DTCs. (60× Zoom2, Bar = 20 µm). GFP-expressing DTCs captured by flow cytometry with HLA-ABC (APC conjugated) from the murine marrow under (**H**) fluorescent microscope (20×, Bar = 100 µm) and (**I**) confocal microscope (60× Zoom2, Bar = 10 µm). DIC: Differential interference contrast.

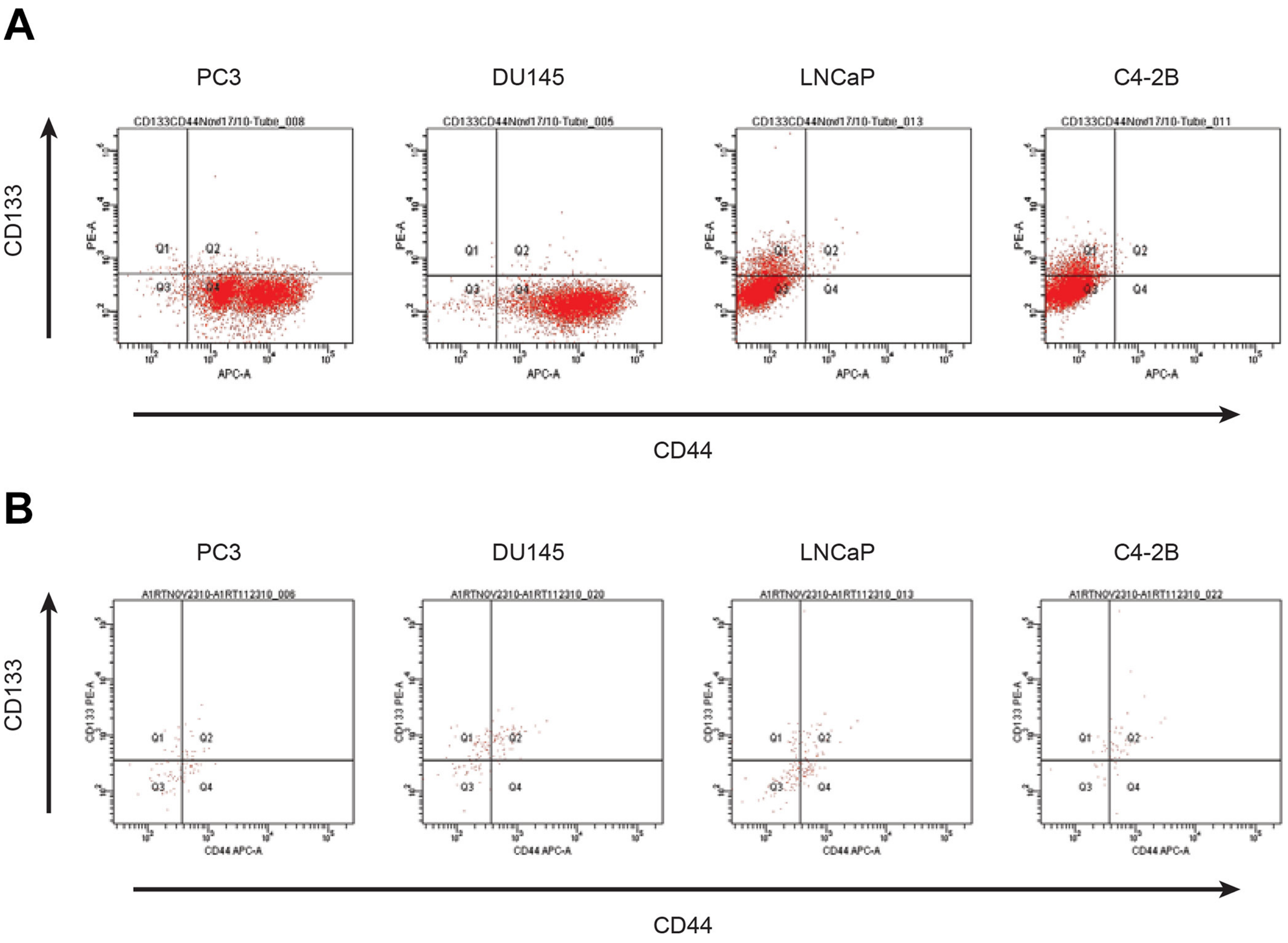

Supplementary Figure S2: Enrichment of CSC population in disseminated prostate cancer cells. Representative scatter dot-plot images of flow cytometry for CD133+/CD44+ (CSC) (**A**) *in vitro* (Figure 1C, 1D, 1E, 2G, 3G, 4A, 6D, 6F, and S4B) and (**B**) *in vivo* (Figure 1D, 1E, 2A, 2B, 2H, 6E, and 6G).

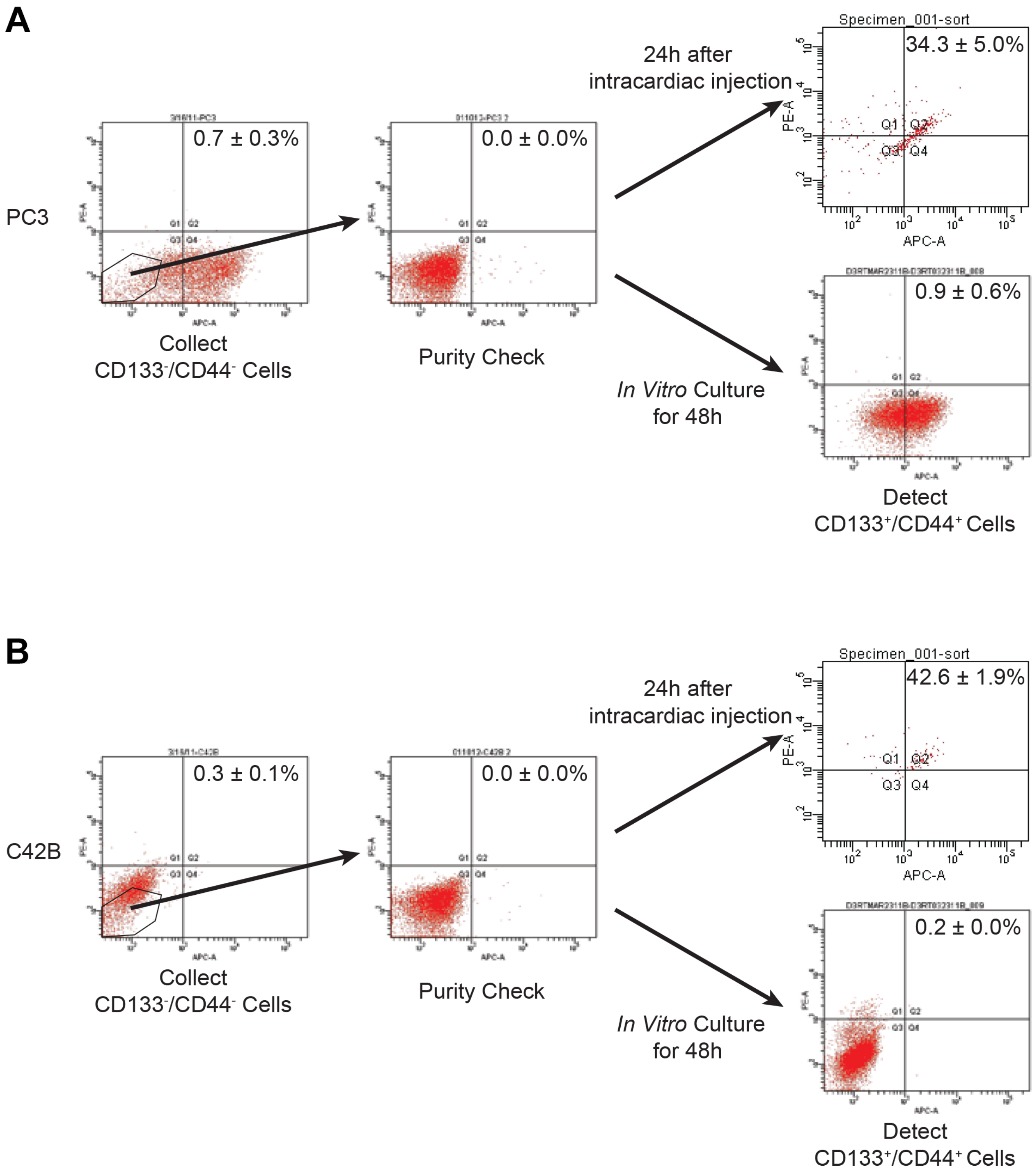

Supplementary Figure S3: The conversion of non-CSCs to CSCs. Representative scatter dot-plot images of gating for $CD133^{-}$/ $CD44^{-}$(non-CSC) sorting and purity check in Figure 1D.

A

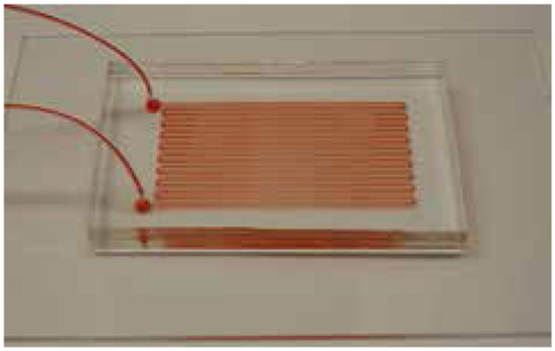

B

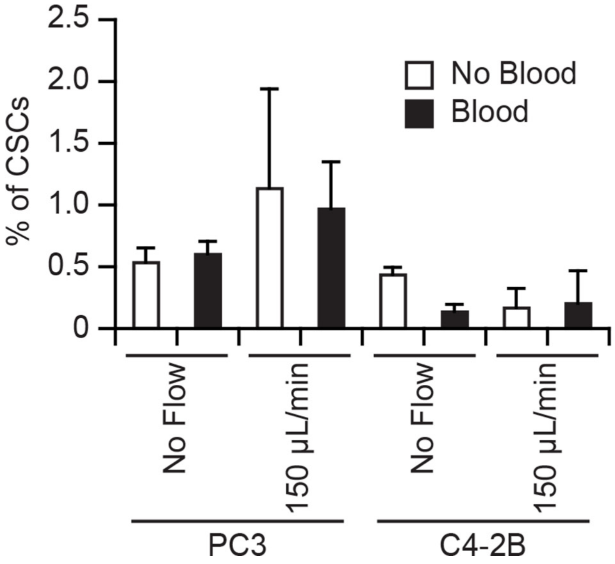

Supplementary Figure S4: *In vitro* survival assays with a microfluidic device. (**A**) Microfluidic devices were fabricated by using soft lithographic techniques. Microfluidic devices were fabricated by using soft lithographic techniques [1–3]. An SU-8 50 photoresist (MicroChem, Newton, MA) was spun at 500 rpm for 10 sec then, at 1850 rpm for 60 sec resulting in a 50 µm-thick layer on a silicon wafer. The wafer was soft-baked at 65°C for 6 min, then at 95°C for 20 min on a hot plate. The wafer was then exposed to UV light, baked at 65°C for 2min, and then at 95°C for 5 min. Finally, the wafer was developed for 3 min and rinsed with IPA and dried with nitrogen gas. Poly(dimethylsiloxane) (PDMS) (Sylgard 184, Dow Corning, Midland, MI) was poured onto the SU-8 mold and cured at 65°C for 4 h. The PDMS layer was peeled from the mold and inlet and outlet holes were punched using a Harris Uni-Core punch. The prepared PDMS layer was bonded to a clean glass substrate by corona discharge treatment [4]. The overall chip size is 42 mm × 27 mm. The total length of the microchannel is 600 mm. The microchannel varies in width between 20 µm and 320 µm to apply incremental shear stress to cells. The device was washed with EDTA solution before flowing cells. 1,000,000 prostate cancer cells/mL with blood or without blood was injected into the device. The flow rate was varied from 50–150 µL/min. (**B**) Comparison of CSC levels when prostate cancer cells were inoculated into a shear stress chamber in the presence/absence of whole blood.

1. Whitesides GM. The origins and the future of microfluidics. Nature. 2006; 442:368–73.
2. Duffy DC, McDonald JC, Schueller OJ, Whitesides GM. Rapid Prototyping of Microfluidic Systems in Poly(dimethylsiloxane). Analytical chemistry. 1998; 70:4974–84.
3. Ng JM, Gitlin I, Stroock AD, Whitesides GM. Components for integrated poly(dimethylsiloxane) microfluidic systems. Electrophoresis. 2002; 23:3461–73.
4. Haubert K, Drier T, Beebe D. PDMS bonding by means of a portable, low-cost corona system. Lab on a chip. 2006; 6:1548–9.

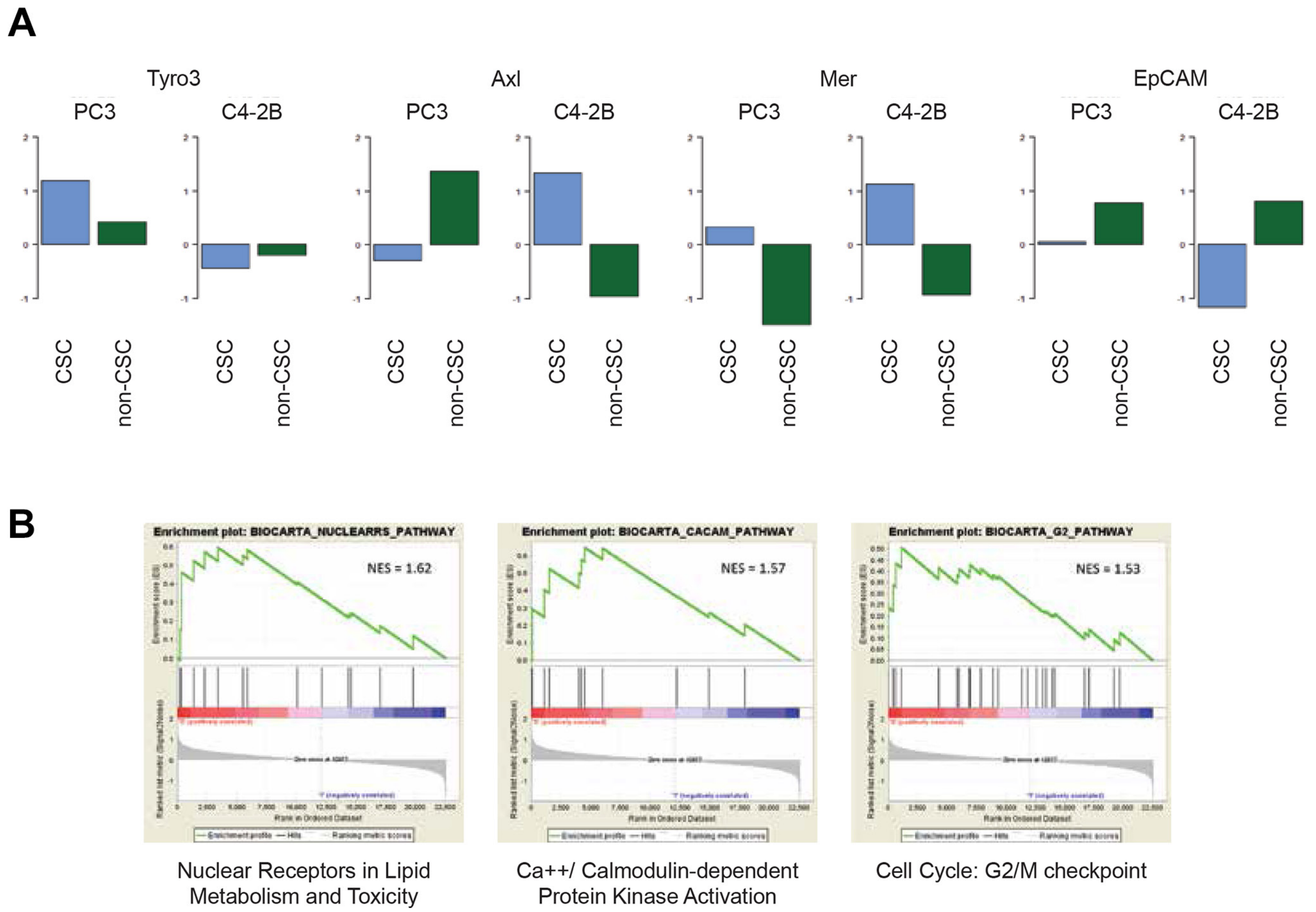

Supplementary Figure S5: Gene expression differences between *in vivo* CSCs and non-CSCs. (**A**) Individual comparison of representative genes (Tyro3, Axl, Mer, and EpCAM) from Figure 3B. (**B**) Enrichment plots of gene sets (Biocarta) enriched in the CSC population from Figure 3B. The Biocarta pathways are: Nuclear Receptors in Lipid Metabolism and Toxicity; Ca++/Calmodulin¬dependent Protein Kinase Activation; and Cell Cycle: G2/M Checkpoint.

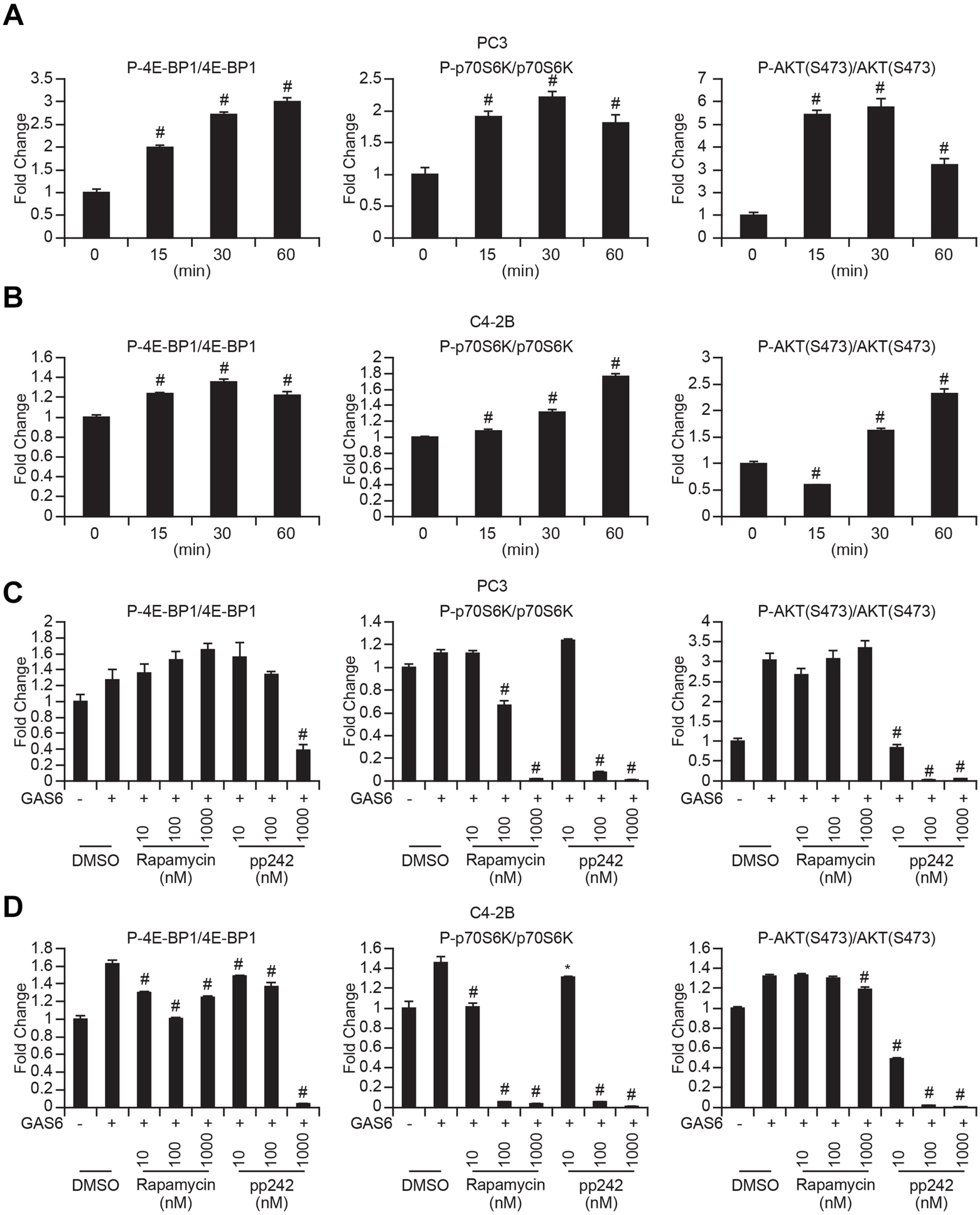

Supplementary Figure S6: GAS6 activates mTOR signaling in prostate cancer. Densitometric analysis of Western blot image ((**A**) PC3 and (**B**) C4-2B) in Figure 4D. Significance vs. 0min. Densitometric analysis of Western blot image ((**C**) PC3 and (**D**) C4-2B) in Figure 4E. Significance vs. GAS6 treated prostate cancer. $^*p < 0.05$ and $^\#p < 0.01$ (Student's *t*-test).

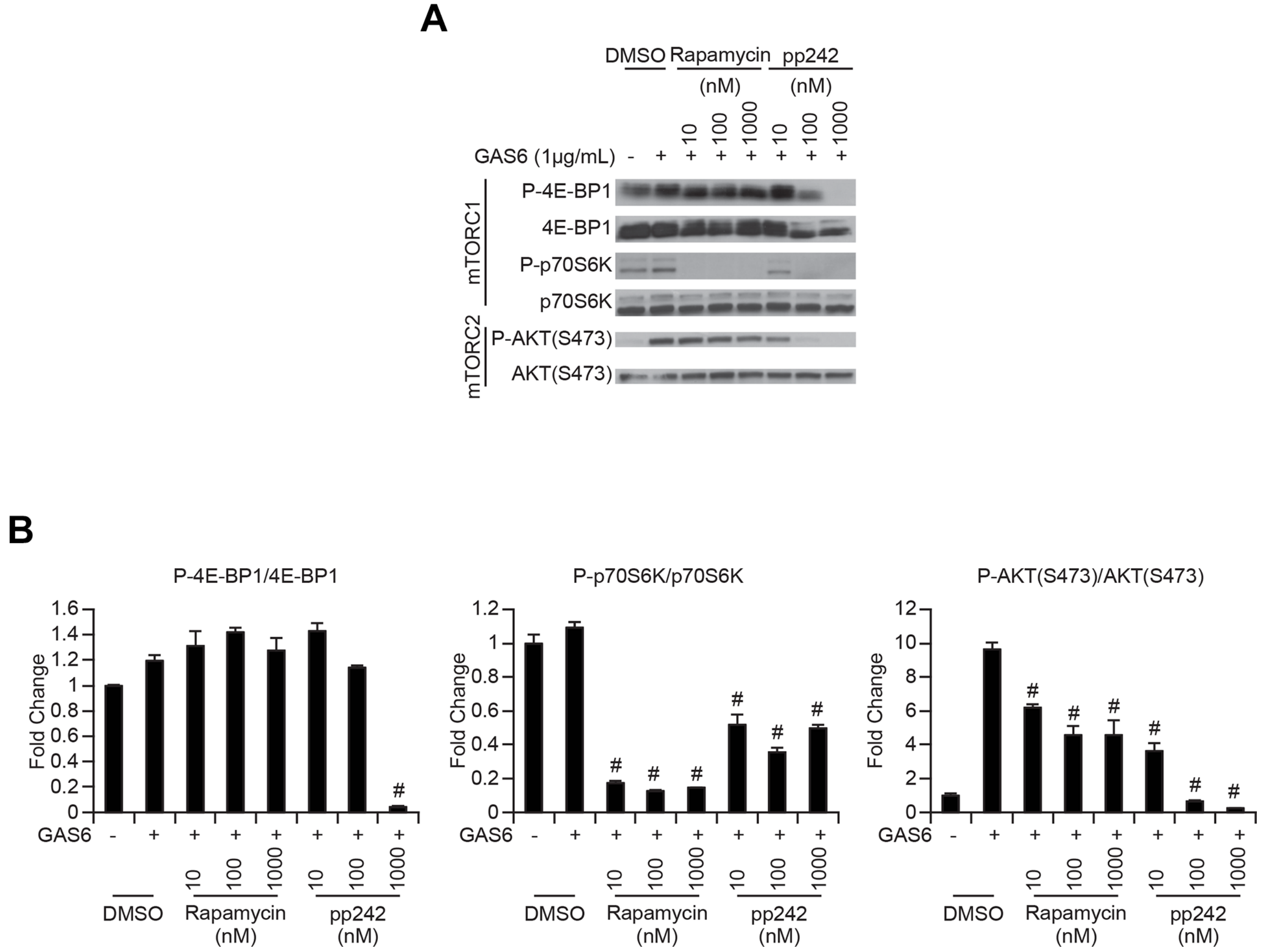

Supplementary Figure S7: GAS6 activates mTOR signaling in DU145. (**A**) Activation of mTOR signaling with GAS6 treatment in DU145 in the presence/absence of mTOR inhibitors. (**B**) Densitometric analysis of Western blot image in Figure S7A. Significance vs. GAS6 treated DU145.

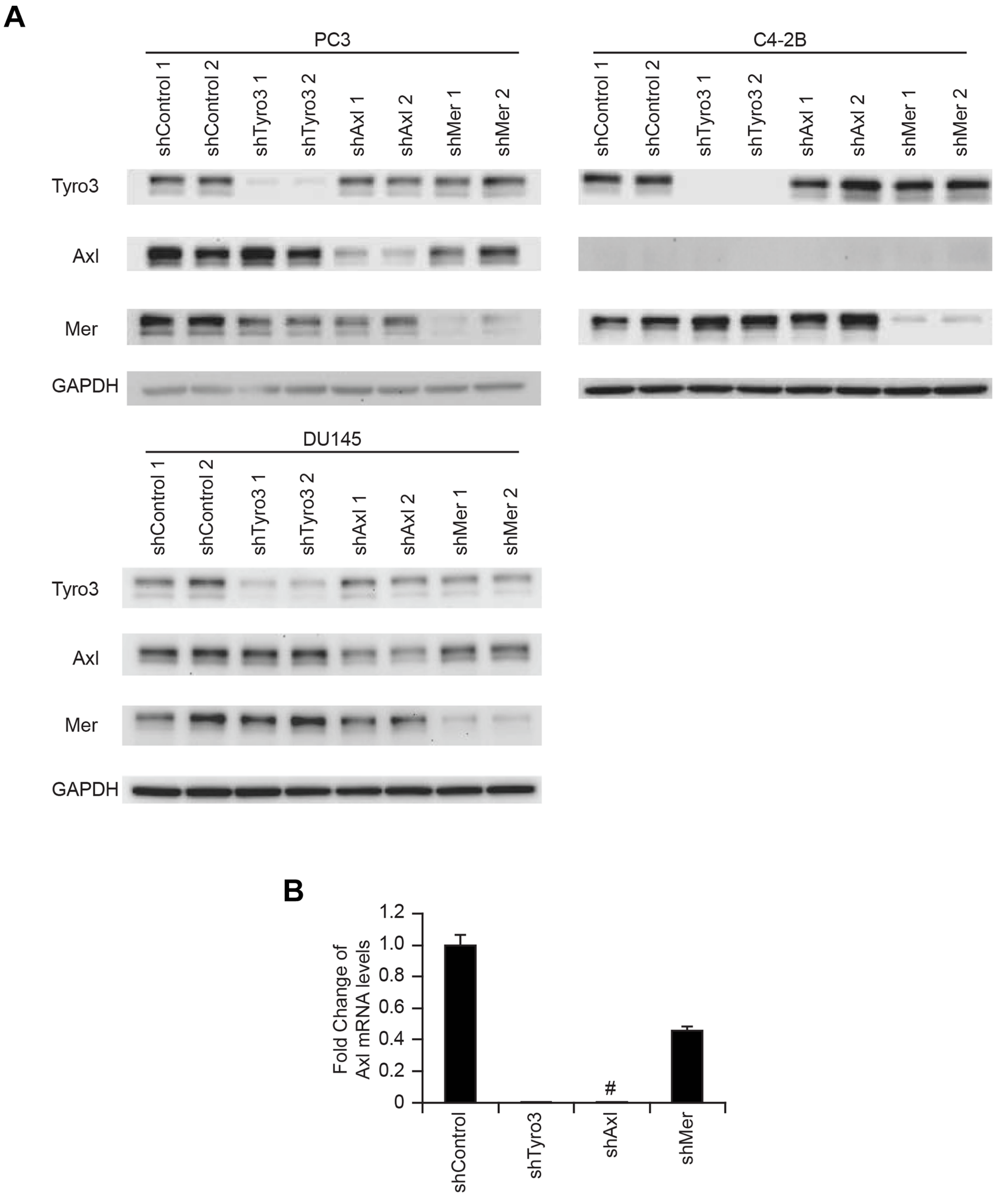

Supplementary Figure S8: Knock-down of receptors for GAS6 in prostate cancer. (**A**) Protein expression of Tyro3, Axl, and Mer in PC3, C4-2B, and DU145 transduced with lenti-shControl, -shTyro3, -shAxl, and -shMer. (**B**) mRNA expression of Axl in C4-2B transduced with lenti-shControl, -shTyro3, -shAxl, and -shMer.

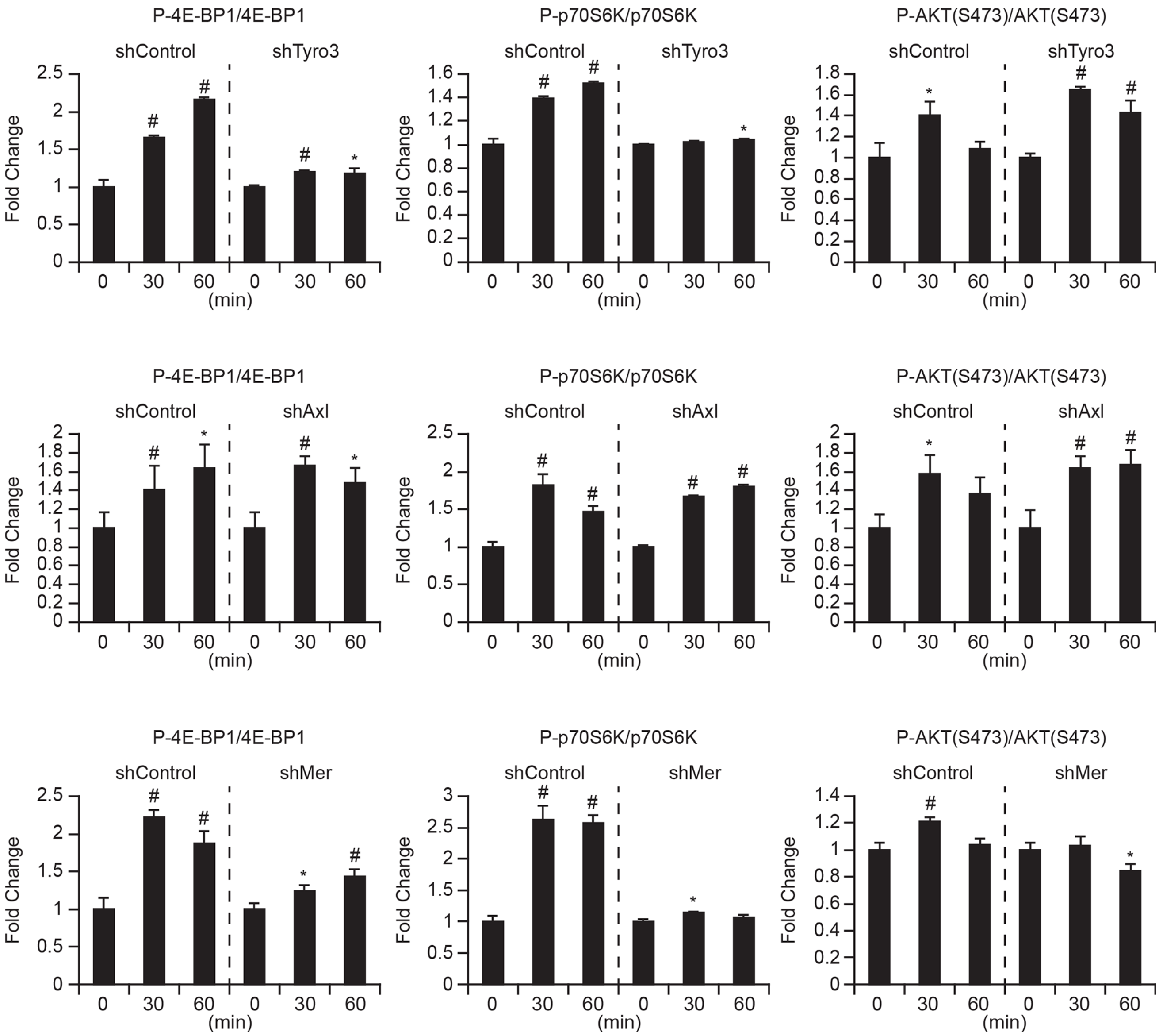

Supplementary Figure S9: GAS6 activates mTOR signaling through Mer in PC3. Densitometric analysis of Western blot image in Figure 5A. Significance vs. 0 min. *$p < 0.05$ and $^{\#}p < 0.01$ (Student's *t*-test).

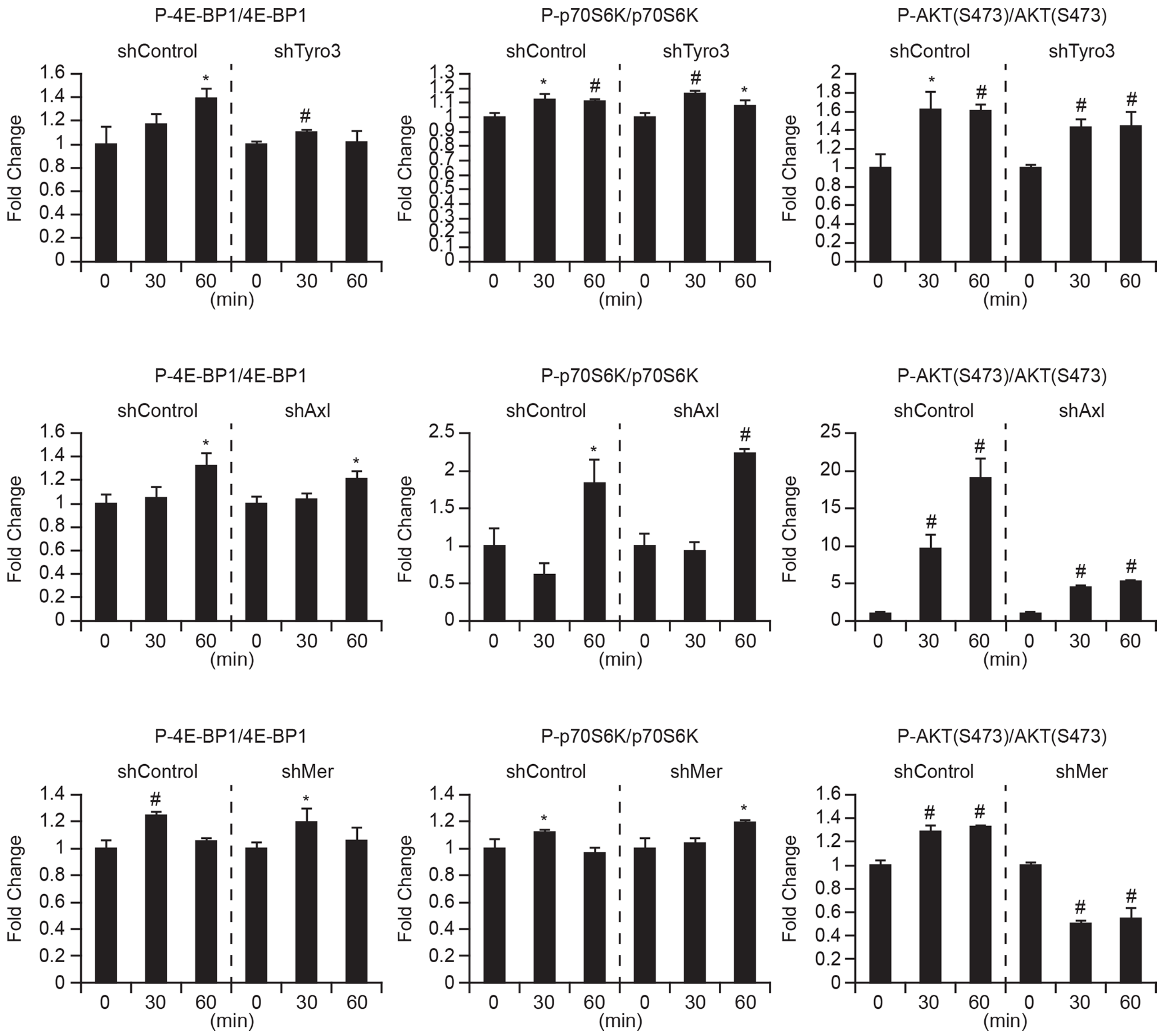

Supplementary Figure S10: GAS6 activates mTOR signaling through Mer in C4-2B. Densitometric analysis of Western blot image in Figure 5A. Significance vs. 0 min. *$p < 0.05$ and #$p < 0.01$ (Student's *t*-test).

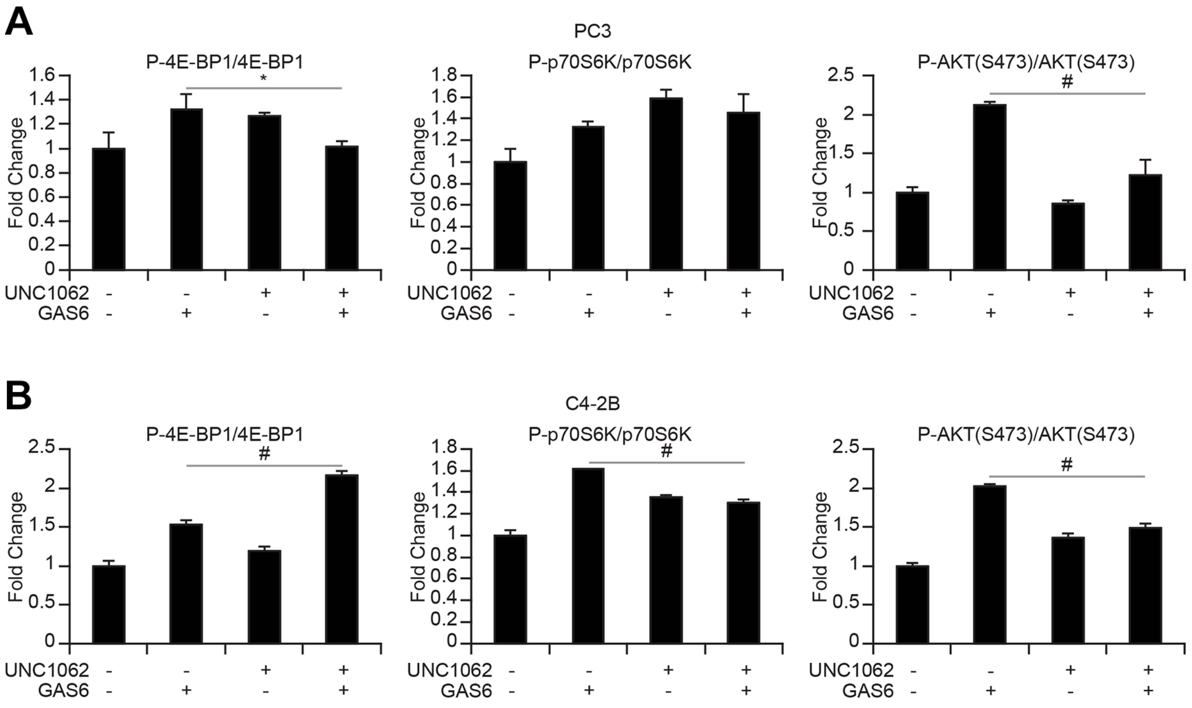

Supplementary Figure S11: Mer inhibitor influences mTOR signaling activated by GAS6 in prostate cancer. Densitometric analysis of Western blot image in Figure 5B. *$p < 0.05$ and $^{\#}p < 0.01$ (Student's t-test).

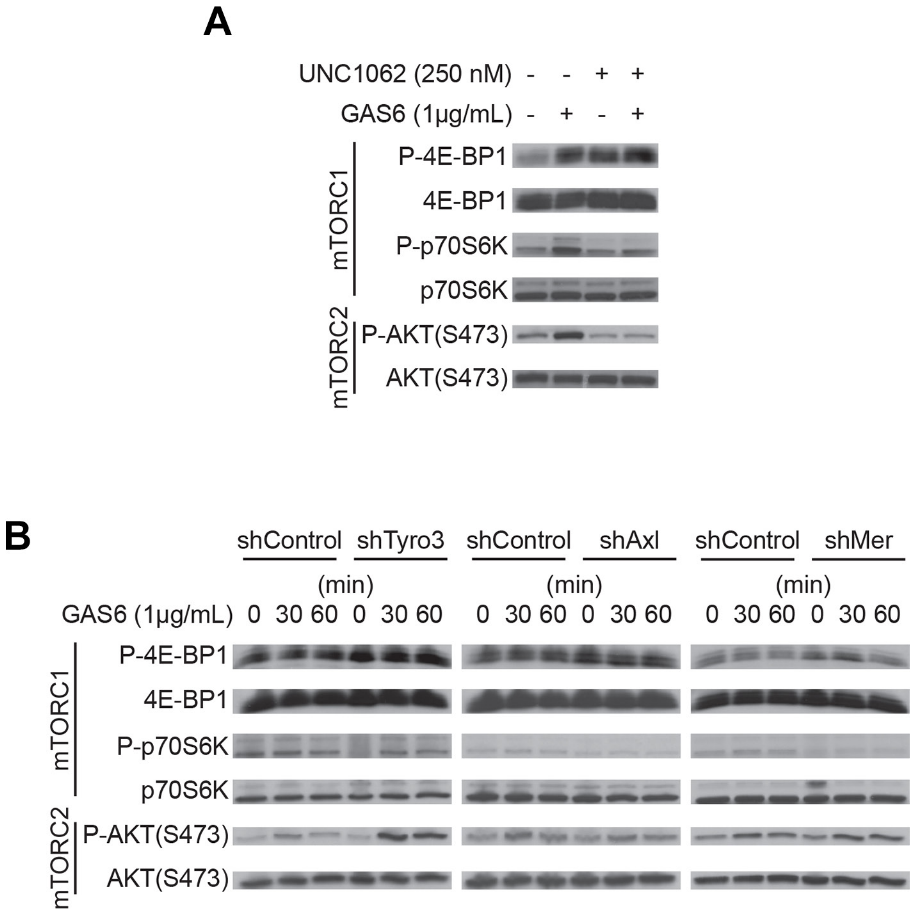

Supplementary Figure S12: GAS6 activates mTOR signaling through Mer in DU145. (**A**) Activation of mTOR signaling with GAS6 treatment in DU145 in the presence/absence of Mer inhibitor (UNC1062). (**B**) Activation of mTOR signaling with GAS6 treatment in DU145 with TAM (Tyro3, Axl, Mer) receptors knocked down.

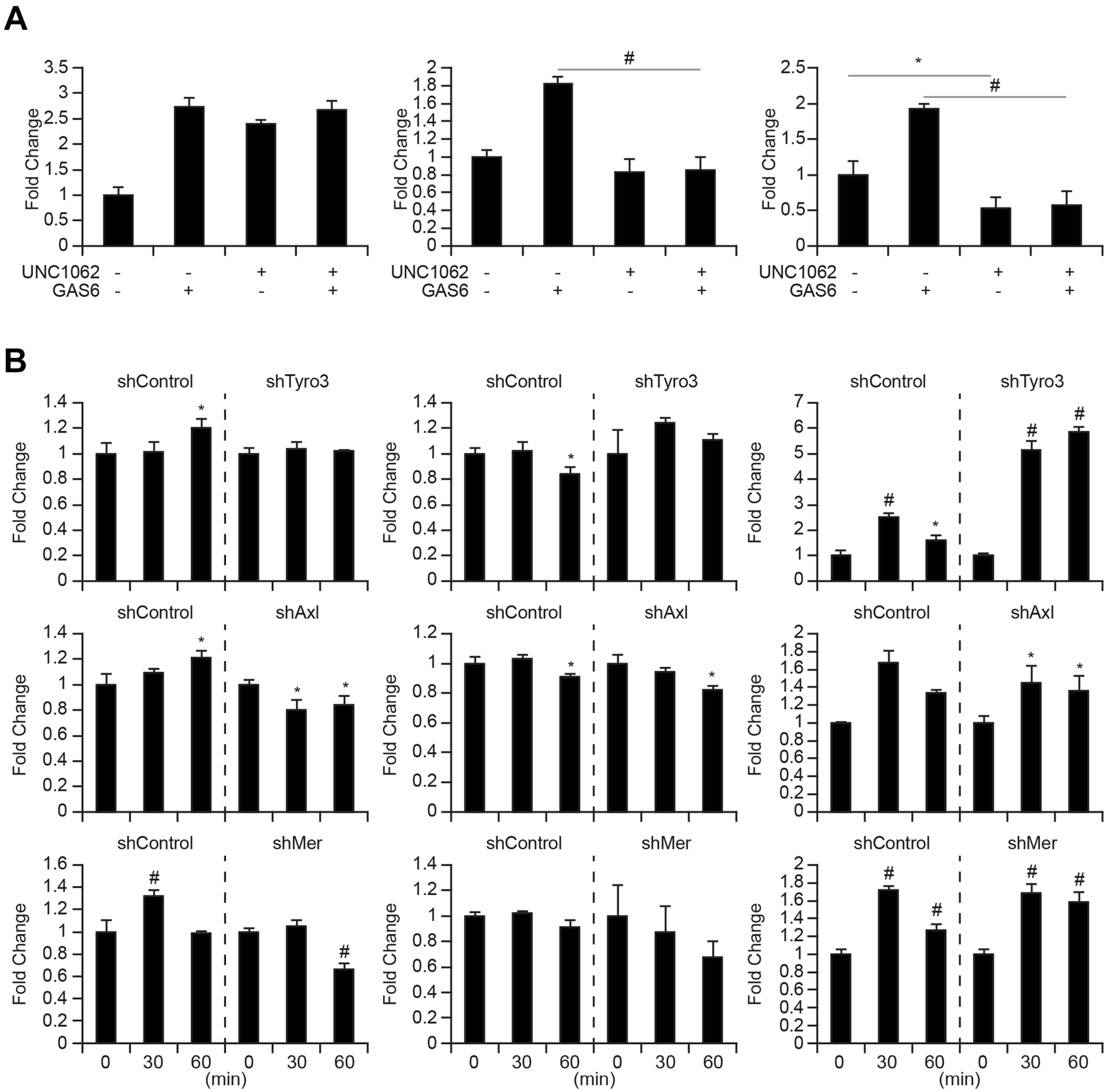

Supplementary Figure S13: GAS6 activates mTOR signaling through Mer in DU145. (**A**) Densitometric analysis of Western blot image in Figure S12A. (**B**) Densitometric analysis of Western blot image in Figure S12B. Significance vs. 0 min. *$p < 0.05$ and #$p < 0.01$ (Student's *t*-test).

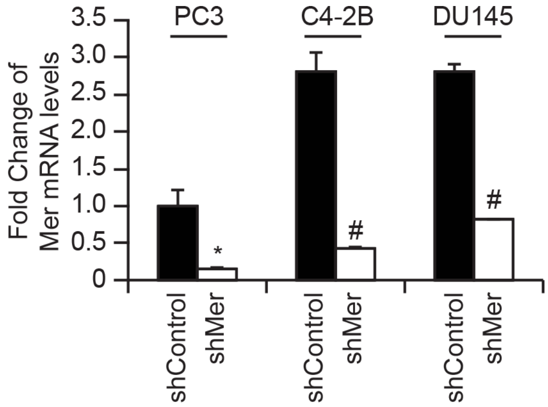

Supplementary Figure S14: Knock-down of Mer in prostate cancer. mRNA expression of Mer in PC3, C4-2B, and DU145 transduced with lenti-shControl and ¬shMer. Significance vs. lenti-shControl prostate cancer. **p* < 0.05 and $^{\#}p < 0.01$ (Kruskal-Wallis test).

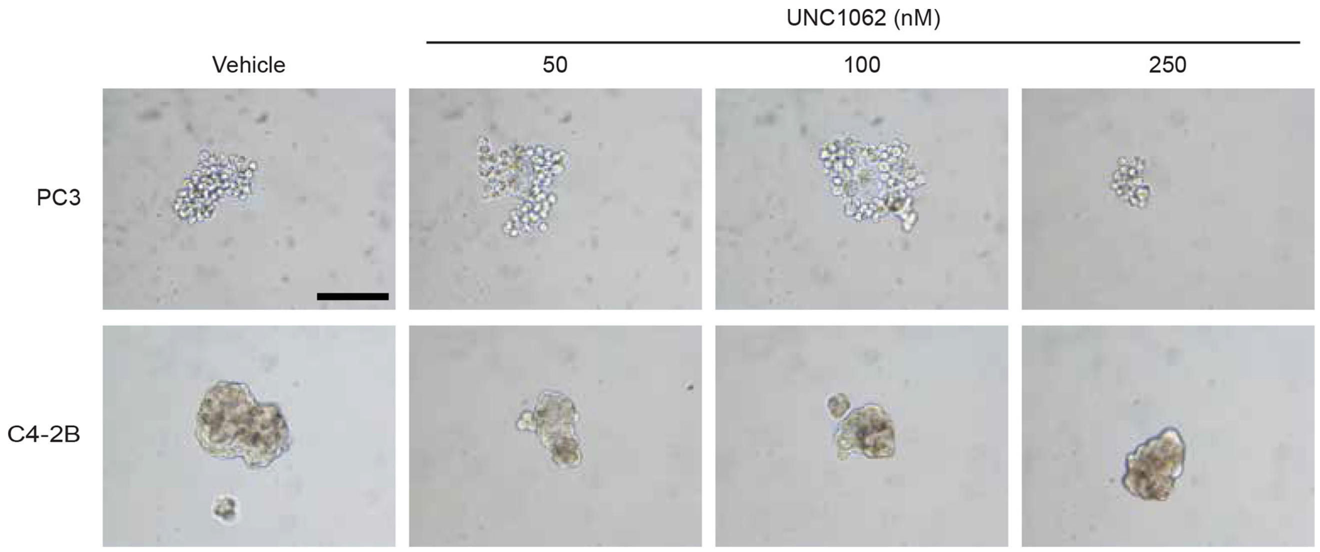

Supplementary Figure S15: Mer may control the sphere-forming ability of prostate cancer. Representative images of *in vitro* sphere formation assays in Figure 6A and 6B. The images were taken of live cells with a Nikon Eclipse TS100 inverted microscope under phase contrast using a 20×/0.4 objective. (Bar = 100 μm).

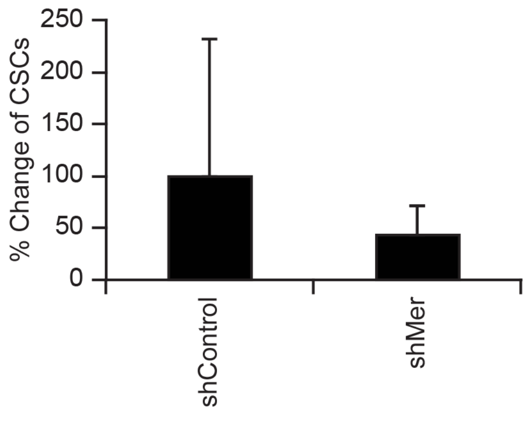

Supplementary Figure S16: Mer may control the conversion of DTCs into CSC. PC3 cells (shControl and shMer) were inoculated intracardially and 24 h later the CSC population in disseminated prostate cancer was measured by flow cytometry (*n* = 5).

Supplementary Table S1: Expression value of genes related to stemness in PC3. See Supplementary_Table_S1

Supplementary Table S2: Expression value of genes related to stemness in Ca–2B. See Supplementary_Table_S2

Supplementary Table S3: Genes differentially expressed between CSC and non-CSC obtained from *in vivo* DTCs. See Supplementary_Table_S3
