# Supplementary material for: The marrow niche controls the cancer stem cell phenotype of disseminated prostate cancer
Source: Oncotarget. 2016 May 9;7(27):41217–32. doi: 10.18632/oncotarget.9251 (PMC5173053; doi:10.18632/oncotarget.9251)
Supplement: Supplementary file 2 [file oncotarget-07-41217-s002.doc]

Supplemental Table1. Expression value of genes related to stemness in PC3

ProbeID          Symbol           Name	EntrezGene   UniGene	In Vitro non-­‐CSC1   In Vitro non-­‐CSC2        In Vitro CSC1            In Vitro CSC2        In Vivo non-­‐CSC1     In Vivo non-­‐CSC2         In Vivo CSC1	In Vivo CSC2
16948461 SOX2	SRY (sex determining region Y)-­‐box 2	6657  Hs.518438//Hs.732963	1.497297236	1.69580453	1.946711923	1.709735223	2.230813905	2.048939447	2.308920113	2.469579088	
17025989 DLL1	delta-­‐like 1 (Drosophila)	28514 Hs.379912	2.392578239	1.825764821	2.266186544	2.40084453	1.721913378	3.006340044	3.885676747	2.037705449	
16707493 HHEX	hematopoietically  expressed homeobox	3087 Hs.118651	2.794990456	2.349813793	2.816175384	2.472032315	2.382724655	3.411100232	2.164760892	1.781909065	
16741399 FGF4	fibroblast growth factor 4	2249 Hs.1755	1.725373445	1.930225785	1.723155023	1.62625053	3.49026821	1.815906637	2.680620758	2.146287189	
17076415 FGFR1	fibroblast growth factor receptor 1	2260 Hs.264887	2.130980894	1.265680873	3.111799781	1.727589854	2.140795752	2.130909663	2.088128883	1.920883866	
16980762 SFRP2	secreted frizzled-­‐related protein 2	6423 Hs.481022	1.904536612	1.860874336	1.610449349	1.426679574	1.899862604	2.811336476	2.019030793	1.904842287	
16907863 ERBB4	v-­‐erb-­‐b2 avian erythroblastic leukemia viral oncogene homolog 4	2066 Hs.390729	1.431888427	1.394388635	1.384383806	1.304240914	2.436511317	2.949114422	1.792242827	2.037384147	
16677391 PROX1	prospero homeobox 1	5629   Hs.741808//Hs.744931	1.385389116	1.400298767	1.690062392	1.651698275	2.571018032	2.872568013	2.591591048	1.715615012	
17080648 HAS2	hyaluronan synthase 2	3037 Hs.159226	7.436185618	8.067857299	7.326214616	7.818358184	1.770902817	3.728652042	1.635609621	3.276261167	
16920370 SALL4	spalt-­‐like transcription factor 4	57167 Hs.517113	2.26800062	2.111619072	1.937896681	1.878984856	3.242919225	2.354706417	2.440666799	2.485707496	
16770664 TBX5	T-­‐box 5	6910 Hs.381715	1.197063047	1.409768069	1.252227613	1.29664819	1.629696831	3.352587758	1.629932814	2.148448661	
16984612 ISL1	ISL LIM homeobox 1	3670 Hs.505	1.598618861	2.299505636	2.03361044	3.325596994	1.570515243	1.658056634	2.059402138	1.613245678	
16707541 CYP26A1	cytochrome P450, family 26, subfamily A, polypeptide 1	1592 Hs.150595	1.389322503	1.270917657	1.287531652	1.032958489	1.825313962	2.041777054	1.8870047	1.622164144	
16882162 TCF7L1	transcription factor 7-­‐like 1 (T-­‐cell specific, HMG-­‐box)	83439 Hs.516297	2.814010146	3.347055098	2.943571212	2.767872121	2.744176912	1.945185485	1.782099065	2.277317855	
16779958 EDNRB	endothelin receptor type B	1910 Hs.82002//Hs.743195	1.130176979	0.906997291	1.222584643	1.385793434	2.682052433	1.379894498	2.550834175	2.437989449	
16660059 PADI4	peptidyl arginine deiminase, type IV	23569 Hs.522969	1.849253296	1.805640819	1.673799326	1.774007825	2.308254799	1.764532302	1.845109395	2.160774103	
16894824 OSR1	odd-­‐skipped related transciption factor 1	130497 Hs.123933	4.64939636	3.778717668	4.153244966	3.842418397	2.094014608	3.209091213	2.326283147	3.194052837	
16686784 TAL1	T-­‐cell acute lymphocytic leukemia 1	6886   Hs.705618//Hs.737706	1.061804469	1.732341967	0.979707676	1.431862416	3.108479557	1.861193364	2.478002506	2.100487132	
16763295 PRICKLE1	prickle homolog 1 (Drosophila)	144165   Hs.524348//Hs.720221	3.92334961	5.156269092	3.140053728	4.179143097	2.32442851	2.04560785	2.760005808	2.14167394	
16792135  NKX2-­‐1	NK2 homeobox 1	7080 Hs.94367	1.534581766	1.204693946	1.709894789	1.320168624	2.054898273	2.840266156	1.817267981	1.832436943	
16793129 BMP4	bone morphogenetic protein 4	652 Hs.68879	2.861817421	3.633791324	2.475442024	3.398946167	1.71288666	2.174845825	2.335077242	2.021974104	
16691327 NGF	nerve growth factor (beta polypeptide)	4803 Hs.2561	3.954438498	3.078786274	4.274484039	2.467479585	1.870690579	3.496478355	2.759813074	1.969297531	
16668785 WNT2B	wingless-­‐type MMTV integration site family, member 2B	7482 Hs.258575	1.963692981	1.659602333	1.1684633	1.568479161	1.647721633	1.900325358	2.78513248	1.495564164	
16876731 SOX11	SRY (sex determining region Y)-­‐box 11	6664 Hs.432638	0.960176219	1.083926473	0.891206713	1.258435444	1.83794428	1.71156283	2.515007426	1.384590248	
16719025 FGFR2	fibroblast growth factor receptor 2	2263 Hs.533683	2.040888355	1.27477639	1.383675872	1.408276674	2.363159514	2.085984248	1.706962214	2.032974711	
16736861 BDNF	brain-­‐derived neurotrophic factor	627 Hs.502182	1.841298272	2.235530804	1.935437585	1.979542964	2.817917726	2.990360885	2.214635554	2.670761597	
16979024 PITX2	paired-­‐like homeodomain 2	5308   Hs.643588//Hs.738484	1.506112987	2.200271249	1.722075144	1.266564897	1.908487702	2.290178206	2.820176754	2.988465076	
16902015 PAX8	paired box 8	7849 Hs.469728	2.601448055	3.103291298	2.022119021	2.007461883	2.476186909	1.702744963	2.167645345	2.970510757	
16951797 EOMES	eomesodermin	8320   Hs.591663//Hs.734211	2.298911824	1.73361031	2.015882216	1.604660855	2.002829388	2.898740796	2.8278331	2.97585614	
16936044 WNT7B	wingless-­‐type MMTV integration site family, member 7B	7477 Hs.512714	2.05638634	2.861632763	2.65097348	1.88475398	2.433211791	2.771108606	2.708686628	2.994610127	
16873491 NANOS2	nanos homolog 2 (Drosophila)	339345 Hs.434218	1.398268042	1.83464714	1.18502436	1.433748467	4.577498045	2.939557332	1.860226258	1.659763169	
16777675 CDX2	caudal type homeobox 2	1045   Hs.174249//Hs.740844	1.982193243	1.987822442	0.986284726	1.753043939	3.286031624	3.332952782	3.046696403	2.173553831	
16927110 TBX1	T-­‐box 1	6899 Hs.173984	2.822858973	2.641810261	2.20407869	2.966177754	2.420648426	2.43792427	2.070979219	2.273756799	
16950989 WNT7A	wingless-­‐type MMTV integration site family, member 7A	7476 Hs.72290	4.007866989	2.340941184	2.675744575	2.30839984	2.999761649	2.488237115	2.801579501	3.381935189	
17064810 SHH	sonic hedgehog	6469 Hs.164537	1.577151361	1.832738806	1.903330402	2.08129833	2.029874873	1.954536687	1.769008988	1.821386138	
16949011 CHRD	chordin	8646 Hs.166186	1.823270416	1.468100972	1.43233091	1.585938085	3.096700733	1.664231395	2.628897575	2.976849397	
16911261 BMP2	bone morphogenetic protein 2	650 Hs.73853	1.725890652	1.648975201	1.95645802	1.578819338	2.184302953	2.045234704	4.249834152	2.196116968	
16845410 ETV4	ets variant 4	2118 Hs.434059	5.174432802	5.386219023	4.779648067	5.269708969	2.553966474	3.450825348	2.011274796	2.83916914	
16921428 SOX18	SRY (sex determining region Y)-­‐box 18	54345 Hs.8619	2.742577627	2.699195525	2.364141068	1.615571601	4.000791449	1.963536919	5.152947605	3.902229472	
16846587 COL1A1	collagen, type I, alpha 1	1277   Hs.172928//Hs.681002	2.585968081	2.156971962	2.172184736	2.009300887	3.77567427	2.484434693	2.178902468	2.326763698	
16718747 VAX1	ventral anterior homeobox 1	11023 Hs.441536	1.634132726	1.200831941	1.463311408	1.650701917	3.8763204	2.880799237	3.182031301	2.099150677	
16658135 PRDM16	PR domain containing 16	63976 Hs.99500	1.199231371	1.385478365	1.363987925	1.465750836	1.411674282	2.419021029	1.627045294	1.581556923	
17094601 FOXD4L5	forkhead box D4-­‐like 5	653427 Hs.714683	1.387019772	0.973629782	1.61191856	1.433297177	1.638330052	2.966268516	2.068506798	1.931196032	
16909482 HTR2B	5-­‐hydroxytryptamine (serotonin) receptor 2B, G protein-­‐coupled	3357 Hs.421649	2.481753531	2.656617519	2.887010714	2.683622502	3.127383266	2.512453935	2.428993638	2.679802254	
16978834 LEF1	lymphoid enhancer-­‐binding factor 1	51176 Hs.743478	2.144835816	1.945536728	2.237988682	1.675092177	2.395663006	2.529849259	2.288148848	2.242393881	
16988728 MEGF10	multiple EGF-­‐like-­‐domains 10	84466 Hs.438709	1.834686577	1.844111165	2.078161799	1.767372123	1.93506106	2.486101976	3.041604608	2.812728889	
16663730 ARTN	artemin	9048 Hs.632404	2.031560553	2.681071972	2.285902346	2.134273357	4.512558078	2.486936518	3.438888195	2.872364399	
17009289 RUNX2	runt-­‐related transcription factor 2	860 Hs.535845	3.790537528	3.086512824	3.067614407	2.68018587	3.330941464	3.777597266	3.52543963	2.384609745	
16938562 TGFBR2	transforming growth factor, beta receptor II (70/80kDa)	7048 Hs.82028//Hs.604277	5.690938612	5.626802862	5.539020408	5.703874445	3.387306511	3.12505444	3.363122352	2.305627458	
16798951 GREM1	gremlin 1, DAN family BMP antagonist	26585 Hs.40098	2.749294044	2.629399052	2.530518936	2.260167712	2.593165963	2.997802539	2.705617959	1.553620186	
16698801 CD34	CD34 molecule	947 Hs.374990	1.885727896	1.497815768	1.557538399	1.152200242	2.323484906	1.569697916	3.414561327	2.123669459	
16765315 SP7	Sp7 transcription factor	121340 Hs.209402	1.990453294	1.898220212	2.07546969	1.746768536	2.664199994	3.389392419	4.146958309	2.513261314	
16934881 SOX10	SRY (sex determining region Y)-­‐box 10	6663 Hs.376984	2.101687908	1.48659799	1.811586559	1.695411272	3.742689851	2.028976279	3.414561327	3.58354941	
16821654 FOXF1	forkhead box F1	2294 Hs.155591	2.134174675	2.24099062	2.409318557	2.018956336	3.485971219	3.59803519	3.615116195	3.532734059	
17088886 CRB2	crumbs family member 2	286204   Hs.568340//Hs.710092	1.983402139	2.154666655	1.772626025	2.101036667	4.15343058	3.226411464	3.108928151	4.238009821	
16708293 PAX2	paired box 2	5076 Hs.155644	1.522393648	1.607073379	1.606915829	1.99193686	3.510963857	4.468483446	3.884351452	3.230740714	
16755958 ASCL1	achaete-­‐scute family bHLH transcription factor 1	429 Hs.703025	2.139286394	1.706303511	1.831425896	1.975101571	2.983668365	3.631117135	3.701807292	3.126319782	
16742340 WNT11	wingless-­‐type MMTV integration site family, member 11	7481 Hs.108219	2.164611412	2.764761125	2.341304132	2.810333643	3.682090731	3.540233974	4.331589089	3.288705375	
16889879 NRP2	neuropilin 2	8828 Hs.471200	4.364861808	4.258019347	3.467653645	3.670106735	3.973693462	3.480374294	3.335192057	3.237876596	
16786616 BATF	basic leucine zipper transcription factor, ATF-­‐like	10538 Hs.509964	2.344393076	2.003940354	2.019748297	1.80796388	3.515883154	3.602765312	4.429550849	4.102475516	
16975471 PHOX2B	paired-­‐like homeobox 2b	8929 Hs.87202	1.744600092	1.9577463	1.64710935	2.176937484	3.97704646	3.428434953	2.280532139	3.888351564	
16846246 HOXB4	homeobox B4	3214 Hs.664706	2.88257327	2.120839388	2.385267625	2.303724043	3.032476122	3.635628599	3.207160464	3.666421547	
16821660 FOXC2	forkhead box C2 (MFH-­‐1, mesenchyme forkhead 1)	2303 Hs.436448	2.65911445	2.443920434	2.702343221	2.538097562	2.603272163	2.847755839	2.416382569	3.296262415	
16927309 MED15	mediator complex subunit 15	51586 Hs.517421	3.222409355	3.658010845	2.751017871	2.71030155	2.844394354	2.711498195	2.827897994	2.307482345	
17004208 FOXC1	forkhead box C1	2296 Hs.348883	5.553568622	5.648454045	5.692178322	5.778260702	3.810460723	2.466720652	3.486128087	2.481559351	
16877413 MYCN	v-­‐myc avian myelocytomatosis viral oncogene neuroblastoma derived homolog	4613 Hs.25960	2.910840314	2.974304984	2.653238201	2.566615428	4.120615125	2.911557267	3.195396907	3.920397223	
16909753 GBX2	gastrulation brain homeobox 2	2637 Hs.184945	4.109059578	2.671074229	3.882618013	2.710235488	2.622500608	3.595982595	3.319405111	3.046752962	
17004198 FOXF2	forkhead box F2	2295 Hs.484423	1.433748467	1.760783504	1.900161146	1.448535459	3.345583583	1.938601639	2.439143142	2.760517936	
16780917 IRS2	insulin receptor substrate 2	8660 Hs.442344	4.017472886	3.505802815	3.028835273	3.192630057	3.392494124	1.594843539	2.550270523	2.984839007	
16703715 ZEB1	zinc finger E-­‐box binding homeobox 1	6935 Hs.124503	2.447290233	1.821154358	3.838985047	2.639856026	2.432249733	2.766400971	3.248278003	2.974884058	
16786783 ESRRB	estrogen-­‐related receptor beta	2103 Hs.435845	1.458555909	1.404828661	1.349840231	1.533261531	2.622120331	2.081675336	3.106170262	1.899070738	
16661155 LIN28A	lin-­‐28 homolog A (C. elegans)	79727 Hs.86154	1.053533178	1.020404301	1.352780779	1.149384916	2.224011779	1.62728166	2.833937147	1.781474888	
16974626 LDB2	LIM domain binding 2	9079 Hs.714330	1.60602724	1.680457564	1.633908056	1.727957239	3.040928472	2.080781315	2.86482482	3.237964932	
17050834 WNT16	wingless-­‐type MMTV integration site family, member 16	51384 Hs.272375	1.676175478	1.393485236	1.581163289	1.641938509	2.709267108	2.147231507	2.832273681	1.503046912	
16836896 ACE	angiotensin I converting enzyme	1636 Hs.298469	1.661196419	1.691120265	1.913619743	1.787032781	2.000864791	2.079229904	2.651795643	2.375948231	
17076609 SFRP1	secreted frizzled-­‐related protein 1	6422 Hs.213424	2.040129532	2.15898097	1.569329001	1.498167894	2.466726223	2.675549908	2.326769398	3.051853129	
16836735 TBX2	T-­‐box 2	6909 Hs.531085	2.056099696	2.662120911	2.546214045	1.911366024	3.451332995	2.404979642	2.650685079	2.740337817	
17012304 HEY2	hes-­‐related family bHLH transcription factor with YRPW motif 2	23493 Hs.144287	1.410726562	1.266618513	0.970410435	1.303999761	2.408724632	2.632622264	2.541147776	3.409875114	
16753641 HMGA2	high mobility group AT-­‐hook 2	8091 Hs.505924	6.06614552	6.062682114	6.567182423	6.517938598	2.228110738	1.543056213	2.936172192	1.398605382	
16685704 HEYL	hes-­‐related family bHLH transcription factor with YRPW motif-­‐like	26508 Hs.472566	1.585981998	1.645307601	1.320861015	1.787113859	1.5405675	1.719557	1.693755585	2.623017441	
17067696 NRG1	neuregulin 1	3084   Hs.453951//Hs.668810	1.636033488	1.814239475	2.179265952	1.564308517	3.871388686	2.44216852	2.84065893	2.490389557	
16957254 DPPA2	developmental pluripotency associated 2	151871 Hs.351113	1.156372956	1.082830603	1.004163215	1.03743445	2.061888307	1.428149607	2.381895307	3.37603629	
16683221 WNT4	wingless-­‐type MMTV integration site family, member 4	54361 Hs.25766	2.036604872	2.006318867	2.006318867	2.284003075	3.674411289	1.821894681	3.439103131	2.472773608	
16997953 MEF2C	myocyte enhancer factor 2C	4208 Hs.649965	2.634196325	2.871227674	3.161793938	1.862212461	3.402589852	2.22098816	2.172405869	1.617526411	
16981730 VEGFC	vascular endothelial growth factor C	7424 Hs.435215	7.343511436	7.407652984	7.304073997	6.941252385	2.092944159	2.078852431	3.043306583	2.586189482	
16940172 CCR2	chemokine (C-­‐C motif) receptor 2	729230   Hs.511794//Hs.705362	1.297297615	1.352417186	1.186494758	1.290201846	2.36066289	3.379920439	2.096019649	3.683131547	
17020258 BMP5	bone morphogenetic protein 5	653 Hs.296648	2.12087496	2.538120272	1.742468228	2.440870205	3.671321732	2.582395431	3.421081574	2.987098294	
17002879  NKX2-­‐5	NK2 homeobox 5	1482 Hs.54473	1.445819814	1.346116791	1.314600074	1.128129311	2.146083963	2.06153964	1.865092174	3.247022533	
17059165 HGF	hepatocyte growth factor (hepapoietin A; scatter factor)	3082 Hs.396530	1.363553676	1.152785991	1.810261061	1.108522736	4.037228892	3.022989117	1.990009304	1.868950783	
16908552 IHH	indian hedgehog	3549 Hs.654504	1.279372543	1.609359039	0.768319288	1.178052853	0.90527899	2.917973501	1.234272453	1.908082763	
16903969 ACVR1	activin A receptor, type I	90 Hs.470316	3.508966681	3.838501393	4.002011679	3.584428762	2.52373121	2.688457845	2.974339417	2.615774232	
16693449 S100A4	S100 calcium binding protein A4	6275 Hs.654444	3.772423136	3.128067899	4.169093558	4.017587057	2.285967671	2.169177973	1.863152035	2.066351257	
16705381 TET1	tet methylcytosine dioxygenase 1	80312   Hs.567594//Hs.708977	2.976898152	3.746864845	4.280717687	3.949667731	2.792481544	2.434773827	2.458253875	2.260932892	
16847933 AXIN2	axin 2	8313 Hs.156527	2.015832038	1.876778497	2.058063544	1.9142143	2.289460968	2.81831028	2.347976364	2.522378453	
16948837 ECE2	endothelin converting enzyme 2	9718 Hs.146161	3.221660362	2.657443314	2.255390668	2.330993419	1.764736085	2.204920826	1.994553837	2.47559741	
17004903 EDN1	endothelin 1	1906   Hs.511899//Hs.713645	5.523526395	5.135504207	4.953255285	5.740122324	2.352948099	2.301438515	2.17991838	2.875661126	
16898403 SPRED2	sprouty-­‐related, EVH1 domain containing 2	200734 Hs.59332	3.76453201	4.267987875	4.151366365	4.249165346	2.021620991	3.195947745	2.494666508	3.777446979	
16764500 RACGAP1	Rac GTPase activating protein 1	29127 Hs.505469	2.477228727	2.926003639	2.423906827	2.897529358	1.39247471	1.429318317	1.831726961	1.711998062	
16717733 FBXW4	F-­‐box and WD repeat domain containing 4	6468 Hs.500822	2.196675107	2.481268047	1.460802877	1.923804697	2.250791051	1.538653334	1.179712086	2.360372167	
16889563 FZD7	frizzled class receptor 7	8324 Hs.173859	2.550395862	2.687524696	2.401782232	3.385645716	1.247770551	2.475646372	3.080181262	3.015229692	
16855127 SMAD7	SMAD family member 7	4092 Hs.465087	1.583019184	1.456970218	1.499875394	1.351622664	3.095138579	1.828968414	2.642626924	2.907386913	
17056152 HOXA7	homeobox A7	3204   Hs.610216//Hs.660918	1.523133074	2.030371685	2.441106985	1.959441754	3.187499714	4.0140756	3.177719439	2.833334659	
16744461 DRD2	dopamine receptor D2	1813 Hs.73893	2.07242768	2.364518622	2.204672345	1.952102186	2.347311622	2.81736365	2.384724663	3.460200155	
16933760 LIF	leukemia inhibitory factor	3976 Hs.2250	5.117400644	5.29397888	5.068853237	5.102048827	1.949750886	2.41521686	2.561868244	2.070763696	
17090103 PRRX2	paired related homeobox 2	51450 Hs.660115	2.01613722	1.941369265	2.083922263	1.7241116	2.737924451	2.418018846	3.039177124	3.106314737	
17051392 SMO	smoothened, frizzled class receptor	6608 Hs.437846	2.340467231	1.959286198	2.59392198	1.964812697	2.347580164	2.341549087	2.296355754	1.474103426	
17015540 TFAP2A	transcription factor AP-­‐2 alpha (activating enhancer binding protein 2 alpha)	7020 Hs.519880	4.690209695	4.728617388	5.189935561	5.207174252	1.849776635	2.471173559	1.897587682	1.675642176	
16857389 NRTN	neurturin	4902 Hs.234775	2.642840853	2.209893011	1.725657758	2.709633909	2.326329561	2.080988742	3.556079002	3.936665865	
16964027 FGFR3	fibroblast growth factor receptor 3	2261 Hs.1420	1.380327787	1.278284336	1.209586553	1.160910004	2.900915418	2.411181582	2.104118896	3.093474745	
16847095 RNF43	ring finger protein 43	54894 Hs.584916	1.747900918	2.118291773	1.680759063	1.430729268	1.952009823	1.210520947	1.508405649	1.636498841	
16745870 CDON	cell adhesion associated, oncogene regulated	50937 Hs.38034	2.985079202	3.670734795	3.21371144	3.787884499	2.332374174	2.36375815	2.175029148	1.993164688	
17114288 GPC3	glypican 3	2719 Hs.644108	1.168882643	1.304597002	1.283644851	1.099151192	2.08913913	2.081751686	2.968582068	2.279259764	
16914791 SNAI1	snail family zinc finger 1	6615 Hs.48029	2.799142429	2.973637411	1.823039963	2.013852605	2.12752703	1.961316558	3.107073127	2.265732048	
16926527 POFUT2	protein O-­‐fucosyltransferase 2	23275 Hs.592164	2.996775816	3.482952567	3.609228243	3.892812592	2.885456483	2.4949566	2.882579768	2.316972362	
16657993 SKI	v-­‐ski avian sarcoma viral oncogene homolog	6497 Hs.656507	3.381936488	3.514853791	2.661346895	2.977430369	3.274297257	2.822367531	2.639417983	2.211573615	
16920585 BMP7	bone morphogenetic protein 7	655 Hs.473163	1.710702999	1.855380435	1.709749721	1.386711789	2.648464095	2.839238097	2.251221639	1.962181375	
16917183 JAG1	jagged 1	182 Hs.224012//Hs.626544	6.866568987	6.808555572	6.904936865	6.618903422	1.870465905	2.188074829	2.39245879	3.067830829	
16734040 SCT	secretin	6343 Hs.632324	1.885315947	2.177091757	2.365912919	2.138729075	3.84973833	2.985353872	3.971670957	2.410034009	
16664460 FOXD2	forkhead box D2	2306 Hs.166188	2.89835339	2.521466883	1.644374604	2.888125196	1.998046241	1.571744279	2.318519117	2.574353544	
16794980 TGFB3	transforming growth factor, beta 3	7043 Hs.592317	3.253201513	3.103950359	3.500213465	2.60154126	2.862741655	2.81956946	3.308462174	3.133743964	
16782975 FOXG1	forkhead box G1	2290   Hs.632336//Hs.740590//Hs.741222	1.398136161	1.561833969	1.652855771	1.283581711	1.922665738	1.215435012	2.15248349	1.807731048	
16884732 FOXD4L1	forkhead box D4-­‐like 1	200350 Hs.591554	2.113153066	2.487579644	2.546993599	2.683276319	2.495229917	3.757712327	3.245591251	2.423569272	
17056992 GLI3	GLI family zinc finger 3	2737 Hs.21509	4.527339106	3.796101088	4.538016098	3.658033654	1.773424843	2.218609076	1.915454691	2.121393438	
17101457 AMELX	amelogenin, X-­‐linked	265 Hs.654436	1.426182416	0.976373127	1.66473528	1.303481443	3.6555209	2.22098816	2.507744419	1.375342248	
16715164 NODAL	nodal growth differentiation factor	4838 Hs.370414	1.426813774	2.173725045	1.523724406	1.767124796	2.394722757	2.163170627	2.286128247	3.476313195	
16665507 FOXD3	forkhead box D3	27022 Hs.546573	1.756685312	1.895044137	1.738909775	1.175139569	1.478308766	2.282436234	2.856019438	2.005214204	
17045325 POU6F2	POU class 6 homeobox 2	11281 Hs.137106	1.351264117	1.431341245	1.25497325	1.296192886	2.642684604	1.891682854	2.016373095	1.279081159	
16843853 HNF1B	HNF1 homeobox B	6928 Hs.191144	1.711393835	2.406423856	1.443487793	2.930824425	2.952371159	3.010689889	3.238267504	1.292884	
17016125 GPLD1	glycosylphosphatidylinositol  specific  phospholipase D1	2822 Hs.533291	2.379783459	2.169436783	2.432712351	1.734382383	2.305532482	2.032064257	1.768932408	2.641808111	
16673636 PRRX1	paired related homeobox 1	5396 Hs.283416	1.128498677	1.185564057	1.665943999	0.954379878	3.722832451	2.618017733	2.624775286	1.50739833	
16736120 SOX6	SRY (sex determining region Y)-­‐box 6	55553 Hs.368226	1.180826348	1.0407975	1.138958892	1.291740137	2.258142554	3.103858412	2.518006616	2.435876769	
16762288 SOX5	SRY (sex determining region Y)-­‐box 5	6660 Hs.657542	1.028472499	0.567479126	0.976771244	0.825812298	2.271497685	1.952958878	1.866306329	2.123487895	
16971272 EDNRA	endothelin receptor type A	1909 Hs.183713	1.307461167	0.965687235	1.586999174	1.158575326	1.164230054	2.228560392	2.188262548	1.223199837	
16976029 KDR	kinase insert domain receptor (a type III receptor tyrosine kinase)	3791 Hs.479756	1.386368946	1.49751383	1.537577925	1.270783829	1.40942284	2.481672737	1.927678684	1.856967616	
16981664 GPM6A	glycoprotein M6A	2823 Hs.75819	0.81069826	0.832838917	0.906205217	1.258466351	1.947203541	1.480329797	1.596074526	2.87514634	
16796451 TCL1A	T-­‐cell leukemia/lymphoma 1A	8115 Hs.2484	1.088252851	1.120438407	1.133120485	1.110520239	1.982806457	1.465757411	1.453864187	4.184034035	
16949537 TP63	tumor protein p63	8626 Hs.137569	1.157831345	1.136853744	0.99163711	1.32911818	1.904748504	1.451354551	1.686271915	2.752997978	
17021323 TBX18	T-­‐box 18	9096 Hs.251830	0.964487936	0.900350378	0.930574714	1.175612564	2.036768812	1.377596349	1.762201695	1.376622947	
16717764 FGF8	fibroblast growth factor 8 (androgen-­‐induced)	2253 Hs.57710	1.571856888	1.233689041	1.414195629	1.562738161	1.510881303	2.061644399	2.952985695	2.096405231	
16704154 RET	ret proto-­‐oncogene	5979 Hs.350321	1.021634763	0.869379273	1.278132483	1.294700475	2.425849819	1.910371446	1.925590395	2.544800289	


17095499 GAS1	
growth arrest-­‐specific 1	
2619 Hs.65029	
1.239369753	
1.423412089	
1.097636107	
1.528078468	
1.801514377	
2.846466242	
2.318492198	
2.096259207	
16912401 MYLK2	myosin light chain kinase 2	85366 Hs.86092	1.573260027	1.512545043	1.652862353	1.30872837	3.075489135	1.939093266	2.069210459	1.816689459	
16741393 FGF19	fibroblast growth factor 19	9965 Hs.249200	1.236067968	1.408491828	1.14201558	1.377828391	1.537367563	3.251074238	1.578707545	1.794124256	
16955197 WNT5A	wingless-­‐type MMTV integration site family, member 5A	7474 Hs.643085	4.07474566	3.192251005	3.911260573	3.34761716	1.302207869	1.719376137	2.148993473	1.424140703	
17004721 GCNT2	glucosaminyl (N-­‐acetyl) transferase 2, I-­‐branching enzyme (I blood group)	2651 Hs.519884	3.69485315	1.959835079	3.351821447	3.723417824	2.099890885	1.528091574	1.252737096	1.655354029	
16938701 TRIM71	tripartite motif containing 71, E3 ubiquitin protein ligase	131405 Hs.567678	1.244903132	1.614675875	1.552855222	1.501621832	2.323009347	2.0395229	2.366846956	2.028005913	
17092881 CDKN2A	cyclin-­‐dependent kinase inhibitor 2A	1029 Hs.512599	2.46451829	2.049791462	3.013321298	1.87406459	1.79625789	3.255444822	1.692445677	1.702856939	
17078452 HEY1	hes-­‐related family bHLH transcription factor with YRPW motif 1	23462 Hs.234434	4.279286317	2.745135409	4.554332249	3.967949126	1.981518524	1.783123178	1.832035295	1.748574839	
16961331 MECOM	MDS1 and EVI1 complex locus	2122 Hs.744090	3.734042696	4.256188504	3.370203028	4.44420593	1.875706812	1.955554283	1.898898974	1.603165761	
16966855 KIT	v-­‐kit Hardy-­‐Zuckerman 4 feline sarcoma viral oncogene homolog	3815 Hs.479754	1.34896961	1.101958409	1.034090027	0.766688713	1.740886807	1.72916071	3.624394219	2.101692841	
17011397 NR2E1	nuclear receptor subfamily 2, group E, member 1	7101 Hs.157688	1.332179103	1.017088251	1.3865607	1.304173019	2.455972488	3.256804283	1.714283628	1.987483744	
17050455 FOXP2	forkhead box P2	93986 Hs.282787	1.10502114	0.84395658	0.860316622	0.807545425	1.238441848	1.541123566	2.383202921	2.682190234	
16831224 MYOCD	myocardin	93649   Hs.462257//Hs.567641	1.052425873	1.039935894	1.027652139	1.032274717	1.945833934	2.147383361	1.987056527	1.606284811	
17055614 TWIST1	twist family bHLH transcription factor 1	7291 Hs.66744//Hs.644998	3.832110928	3.36090705	3.732930996	2.92581043	1.478693433	1.684984472	2.272991034	2.37474799	
16906175 FRZB	frizzled-­‐related protein	2487 Hs.128453	1.065177609	1.141574806	1.700479839	0.762836896	1.522911715	1.584169992	1.137761768	1.69384009	
17012761 TCF21	transcription factor 21	6943 Hs.78061	0.942851329	1.019300951	0.942290587	1.106957463	2.415586819	1.378224579	1.305958009	2.301227484	
17065780 GATA4	GATA binding protein 4	2626 Hs.243987	1.095134358	1.093632433	1.10369934	1.113769717	1.76331154	1.905658848	2.317985823	3.082335566	
16995989 FGF10	fibroblast growth factor 10	2255 Hs.664499	1.102074606	1.350142603	1.536023385	0.966357195	2.140849012	1.999983993	2.174421986	1.54782064	
16761012 A2M	alpha-­‐2-­‐macroglobulin	2 Hs.212838	1.029650407	1.30032599	1.615680979	1.090313835	2.323149379	1.620017793	2.294529942	1.672952605	
16675158 PRG4	proteoglycan 4	10216 Hs.647723	1.360812736	1.404680411	1.723774935	1.287514372	1.728837168	1.453161949	1.482987266	2.67239481	
16908928 PAX3	paired box 3	5077 Hs.42146	1.187614452	1.385350699	1.42070182	1.216342735	1.97749303	1.989829933	1.323749604	1.71701033	
16675578 PTPRC	protein tyrosine phosphatase, receptor type, C	5788 Hs.654514	1.158057844	1.018841602	1.558206975	1.237220131	1.676755365	1.500300121	2.888226021	2.099886562	
16707534 CYP26C1	cytochrome P450, family 26, subfamily C, polypeptide 1	340665 Hs.369993	0.929480551	1.315811208	1.337065585	1.276846674	2.27960795	1.421802476	3.397093608	1.336586091	
16737056 PAX6	paired box 6	5080   Hs.270303//Hs.611376	3.76514485	2.770595165	2.54138562	2.802950323	2.145113305	1.576199929	1.657743775	2.159455805	
16995645 DAB2	Dab, mitogen-­‐responsive phosphoprotein, homolog 2 (Drosophila)	1601 Hs.696631	3.613227629	3.808923439	3.672167462	3.972446768	1.549126107	1.424488514	0.930884939	1.770358719	
17059491 ABCB1	ATP-­‐binding cassette, sub-­‐family B (MDR/TAP), member 1	5243   Hs.489033//Hs.737655	1.248361078	1.021768701	0.978384638	1.134643539	1.304282345	1.42516244	1.363859228	1.701758275	
16752147 NEUROD4	neuronal differentiation 4	58158 Hs.591024	0.906735702	1.409775865	1.057503726	1.284321981	3.048808471	2.590040667	1.83928605	1.550727517	
16774303 RGCC	regulator of cell cycle	28984 Hs.507866	1.307033141	2.331017337	2.300717883	1.31148523	1.820243946	1.453161949	1.547515178	2.952045312	
16995461 GDNF	glial cell derived neurotrophic factor	2668 Hs.248114	1.117635001	1.734491302	1.321329237	2.053904226	2.535025262	2.500943864	2.246879818	2.195538461	
17062127 WNT2	wingless-­‐type MMTV integration site family member 2	7472 Hs.567356	1.272947725	0.848732547	1.123255599	0.890342309	3.29810423	2.56321953	1.950866545	2.258269397	
16749939 LRRK2	leucine-­‐rich repeat kinase 2	120892 Hs.187636	1.178197061	0.824862224	0.841476219	1.128616326	2.063330408	1.892851286	2.002020556	1.609634139	
17066573 PIWIL2	piwi-­‐like RNA-­‐mediated gene silencing 2	55124 Hs.614809	0.942576242	0.880465399	0.875519726	0.987572929	1.268618616	1.831365261	2.19932089	1.181622107	
16780208 DCT	dopachrome  tautomerase	1638 Hs.301865	1.027764729	0.759147952	1.038831697	1.232126804	1.630980987	1.760508789	1.234729773	1.847440618	
16773086 FGF9	fibroblast growth factor 9	2254 Hs.111	0.652980325	0.828408017	0.934723499	1.078009297	1.383778494	1.955299028	2.184124782	1.591113966	
16989619 WNT8A	wingless-­‐type MMTV integration site family, member 8A	7478 Hs.591274	1.151772194	1.072338363	0.832295906	0.83122345	2.215584269	1.307773151	2.439802397	1.803468625	
16809827 ALDH1A2	aldehyde dehydrogenase 1 family, member A2	8854 Hs.643455	0.8271318	0.783849274	0.924145118	1.161534045	1.819467718	1.279875375	1.679734252	1.26998756	
16680690 HES5	hes family bHLH transcription factor 5	388585 Hs.57971	1.332427049	0.867713736	0.928981724	1.050840417	1.603449856	3.028774731	2.263866514	1.669428152	
16754880 ALX1	ALX homeobox 1	8092 Hs.41683	1.158819625	1.002438076	1.052444731	0.875523583	1.401168691	1.180705963	1.128252386	1.365354698	
16800921 FGF7	fibroblast growth factor 7	2252 Hs.567268	0.866224167	0.696637469	0.414072031	0.483177726	1.28473958	1.687635654	0.718822735	0.835026397	
16961172 BCHE	butyrylcholinesterase	590 Hs.420483	1.491788265	1.521735275	1.12984052	1.164035937	1.44774524	1.833904332	1.553786817	1.629910185	
16793613 SIX1	SIX homeobox 1	6495 Hs.633506	1.249732463	2.270171905	1.208016386	1.26123917	0.872805887	0.918736887	1.142425149	1.005519111	
16971102 HHIP	hedgehog interacting protein	64399 Hs.507991	1.783710101	0.739552603	1.669825392	1.471027742	1.065106257	1.961596578	1.076630199	1.406460505	
17099248 MED27	mediator complex subunit 27	9442 Hs.374262	1.508926959	1.166916223	0.733301671	1.363466298	0.539783563	0.853601941	0.721275075	0.567966428	
16747852 NANOG	Nanog homeobox	79923 Hs.635882	1.428857386	0.985433979	0.878842251	0.646235463	1.346556725	2.003285916	2.459594522	3.077648191	
16767247 IFNG	interferon, gamma	3458 Hs.856	0.695755313	0.75462759	0.693100341	0.685379433	0.90527899	0.853680359	0.998026458	1.235224399	
16814572 SOX8	SRY (sex determining region Y)-­‐box 8	30812 Hs.243678	1.755923112	1.393951896	1.611567682	2.76611077	2.234891216	1.994213547	2.706594359	1.528354213	
16988021 APC	adenomatous polyposis coli	324 Hs.158932	3.071116428	3.429303552	3.464841129	3.392005569	2.722127727	2.147701671	1.535716758	2.438798096	
16740553 CFL1	cofilin 1 (non-­‐muscle)	1072 Hs.170622	2.682217632	2.443395107	2.111537486	2.490484023	2.614725974	1.966112611	2.41737717	2.099313631	
17064482 SMARCD3	SWI/SNF related, matrix associated, actin dependent regulator of chromatin, subfamily d, member 3	6604 Hs.647067	3.089057442	2.988457242	2.619217392	3.137328497	3.322801285	3.428434953	2.477299831	2.899060807	
16802251 SMAD3	SMAD family member 3	4088   Hs.727986//Hs.742270	4.476051479	3.656579304	4.211788694	3.97939666	3.668902873	3.687206626	3.57904695	3.95074081	
16687847 TACSTD2	tumor-­‐associated calcium signal transducer 2	4070 Hs.23582	5.875227812	6.49350774	4.857742335	6.428883823	2.815937311	3.427754261	2.781669355	3.087963423	
16768270 KITLG	KIT ligand	4254 Hs.1048	5.497622757	4.910517467	5.618950236	4.3097355	3.42710251	2.595430564	2.729698028	2.225606317	
16714944 PBLD	phenazine biosynthesis-­‐like protein domain containing	64081 Hs.198158	2.614724881	3.128812416	3.133966609	2.493899421	2.527521104	1.661885584	3.251804212	2.682923141	
16905108 DLX2	distal-­‐less homeobox 2	1746 Hs.419	3.028998335	3.407709995	2.632133032	2.961404119	3.678768711	2.804902025	3.307976641	3.256612342	
17067890 ZNF703	zinc finger protein 703	80139 Hs.744591	3.610643248	3.688734632	2.428236977	2.436891247	2.96588428	2.077061629	2.003948051	3.764372918	
16928699 ZNRF3	zinc and ring finger 3	84133   Hs.604200//Hs.655242//Hs.732114	2.141556349	3.002100804	2.347300736	2.166438174	1.530120317	1.774209633	2.375091796	1.669991867	
16909165 IRS1	insulin receptor substrate 1	3667 Hs.471508	5.509849043	5.185493746	5.93121979	5.568674189	3.785512916	2.823864407	3.877087731	2.822945729	
17100036 NOTCH1	notch 1	4851 Hs.495473	3.028579249	3.588607635	2.801803797	3.084436085	3.585399076	2.57638453	4.150003075	3.35690014	
16725670 DAGLA	diacylglycerol lipase, alpha	747 Hs.241564	4.25802811	4.45650807	3.862733723	4.167614078	3.183319119	3.461229752	2.475400578	3.635357521	
16694689 NES	nestin	10763 Hs.527971	5.219118756	4.429829606	4.536748284	4.370495597	3.996576013	4.030976923	3.71813406	4.491051482	
16899357 LOXL3	lysyl oxidase-­‐like 3	84695 Hs.469045	3.394729758	2.938839295	2.665819704	3.687966277	3.366428008	3.859148836	3.407204457	2.718406791	
17088701 DAB2IP	DAB2 interacting protein	153090 Hs.522378	3.371604973	3.659327051	3.532960462	3.368505125	3.398256706	3.167162747	3.671504822	3.232719028	
16780264 SOX21	SRY (sex determining region Y)-­‐box 21	11166 Hs.187577	3.219881909	3.236833406	3.630540886	3.398498502	4.145214214	3.116455965	3.442104405	3.978674856	
17005223 ID4	inhibitor of DNA binding 4, dominant negative helix-­‐loop-­‐helix protein	3400   Hs.519601//Hs.663469	2.86114781	3.385052852	2.786148472	3.10150883	3.411878267	3.465078826	3.485297359	3.718748249	
16917822 FOXA2	forkhead box A2	3170 Hs.155651	4.497134634	3.703798169	3.904167661	4.177731553	3.336184028	2.879557094	2.982950471	3.658207967	
16855673 BCL2	B-­‐cell CLL/lymphoma 2	596 Hs.150749	3.66248503	4.204099748	3.635835003	4.679791521	3.932981522	3.925892908	4.490117983	2.96887671	
16920939 LAMA5	laminin, alpha 5	3911 Hs.473256	4.501501902	4.23654524	4.007149848	4.463146318	4.209229419	3.138762648	3.443266302	2.858786731	
16781285 GAS6	growth arrest-­‐specific 6	2621 Hs.646346	4.124295492	3.538532195	3.097378969	3.255229923	2.702692847	3.717645532	3.581550242	3.055090771	
16853277 NFATC1	nuclear factor of activated T-­‐cells, cytoplasmic, calcineurin-­‐dependent 1	4772   Hs.534074//Hs.701518	2.727195138	2.834356075	2.646118339	3.087369376	2.848070611	3.572592403	3.71242149	3.326459496	
16964584 MSX1	msh homeobox 1	4487 Hs.424414	3.284524423	3.072610815	3.765943112	2.865900393	3.117740463	3.092084392	3.283892965	4.35811967	
16915412 EDN3	endothelin 3	1908 Hs.1408	2.864973402	2.606385079	2.738474848	2.551119299	5.714031853	4.516098233	4.570708853	2.722093609	
16872551 TGFB1	transforming growth factor, beta 1	7040 Hs.645227	5.816747785	5.499650118	5.659968234	5.025379802	4.041966209	3.065933222	3.299778248	4.275743561	
16738007 SPI1	spleen focus forming virus (SFFV) proviral integration oncogene	6688 Hs.502511	2.330835968	2.204927975	2.322076483	2.330376872	4.54934285	5.470655551	4.39744175	3.928005664	
17017102 POU5F1	POU class 5 homeobox 1	5460 Hs.249184	3.806564658	4.226801286	4.237216175	4.693031386	3.482183304	4.638900123	3.059015327	5.097797718	
16750690 WNT1	wingless-­‐type MMTV integration site family, member 1	7471 Hs.248164	3.163767387	2.839888055	2.814065561	2.497508425	4.314829683	3.928052003	3.805009004	3.862112422	
17025576 T	T, brachyury homolog (mouse)	6862   Hs.389457//Hs.742027	2.173764247	2.516660277	2.176409355	2.371001791	4.220524184	2.982907879	3.791322883	3.724575927	
16773547 PDX1	pancreatic and duodenal homeobox 1	3651 Hs.32938	2.299782346	2.529621701	2.426706399	2.314590413	3.579007429	4.602332152	4.129130378	2.897731186	
16874214 NTF4	neurotrophin 4	4909   Hs.266902//Hs.743915	1.613506302	2.535247413	2.307959704	1.567552929	2.677699321	2.911557267	3.610450541	3.483786556	
16822408 AXIN1	axin 1	8312 Hs.592082	2.678151281	2.331068516	2.522372784	2.039678679	2.149540598	2.778946036	4.08682348	4.168888812	
16891107 WNT10A	wingless-­‐type MMTV integration site family, member 10A	80326 Hs.121540	1.667540764	2.162192291	2.003127762	2.07390164	3.172507599	2.025801776	2.333845765	2.325078793	
17104363 EFNB1	ephrin-­‐B1	1947 Hs.144700	5.589517815	5.190445122	4.737587388	4.991088038	3.468655154	3.061988344	4.195563433	3.1046425	
16981542 HAND2	heart and neural crest derivatives expressed 2	9464 Hs.388245	2.368281966	2.441090683	2.40886515	2.058257967	3.231226014	2.768308632	3.329392789	3.009967781	
16897026 ZFP36L2	ZFP36 ring finger protein-­‐like 2	678 Hs.503093	4.961540161	5.808462546	5.531731605	6.078408443	4.111074874	2.728834586	3.014139456	2.347100334	
16660182 PAX7	paired box 7	5081 Hs.113253	2.100178335	1.752989272	1.926038847	1.774974447	2.735075085	3.489741557	3.540185299	3.350114729	
16678295 WNT3A	wingless-­‐type MMTV integration site family, member 3A	89780 Hs.336930	2.26750357	1.836495559	1.662448495	2.006370703	3.099163175	2.828816059	2.476632625	3.047581729	
16997434 OTP	orthopedia  homeobox	23440 Hs.202247	2.295334055	2.45769118	2.590830047	2.424994866	2.913196084	3.789442111	3.463293404	2.422243531	
17104578 FOXO4	forkhead box O4	4303 Hs.584654	2.409623453	3.332166958	2.475080756	2.853726285	3.818576086	3.061449583	2.88529971	3.449821286	
16960888 SHOX2	short stature homeobox 2	6474 Hs.55967	1.508742037	2.290354196	2.562234821	2.488316502	3.14742742	2.929228932	2.899820335	2.622262096	
17114585 FGF13	fibroblast growth factor 13	2258 Hs.6540	2.520406523	2.299376775	1.938449727	2.559045221	2.83362454	3.488946221	3.078000854	4.523779041	
16836311 NOG	noggin	9241 Hs.248201	4.214754167	5.102337611	3.145397016	4.229695592	2.339448563	2.711066077	3.334201367	3.619044035	
17068972 SOX17	SRY (sex determining region Y)-­‐box 17	64321 Hs.98367	2.077912853	2.197147527	1.877216617	2.086821885	2.517852164	3.045465454	2.758648631	2.65228574	
16970404 FGF2	fibroblast growth factor 2 (basic)	2247 Hs.284244	6.391499282	6.511390628	6.810008755	6.657788907	1.822958614	2.633582337	2.927040245	2.26047681	
16672635 VANGL2	VANGL planar cell polarity protein 2	57216 Hs.99477	1.659971894	1.965576981	1.549841466	2.448900999	2.541526277	2.253620948	3.507992261	1.903889424	
16960371 WWTR1	WW domain containing transcription regulator 1	25937 Hs.477921	4.159440423	3.794637482	3.054491779	3.740168164	2.694220926	2.595908075	2.791214719	3.251841636	
16818359 TGFB1I1	transforming growth factor beta 1 induced transcript 1	7041 Hs.513530	4.258249354	3.199423415	3.202168612	2.887017206	3.316305905	2.27018327	2.763085561	3.681530948	
17075478 LOXL2	lysyl oxidase-­‐like 2	4017 Hs.626637	4.056247357	3.74473358	4.562143386	3.851707029	2.976535038	2.23263358	3.648233236	1.860909056	
16917531 OVOL2	ovo-­‐like zinc finger 2	58495   Hs.661013//Hs.710157	3.140388288	2.164550742	2.533786288	2.362944418	3.609072893	2.772894668	4.110836357	2.391376862	
16962380 ETV5	ets variant 5	2119 Hs.43697	5.001157806	4.851159601	5.03120014	4.795613688	3.208315495	1.911388207	3.473547508	2.00831846	
17094530 FOXD4L6	forkhead box D4-­‐like 6	653404 Hs.652377	3.414147158	3.869844571	4.096054423	3.752682771	2.661358876	4.30498118	4.002125426	2.534631631	
16903356 ZEB2	zinc finger E-­‐box binding homeobox 2	9839 Hs.34871	1.329473699	0.841852036	1.561843698	1.143827688	4.095151806	3.596131133	4.542151131	3.269034634	
16956149 FOXP1	forkhead box P1	27086 Hs.59368	4.381273721	4.092881929	4.636985101	4.390530052	3.466653355	4.002139028	3.71903653	3.562993352	
16839220 FAM101B	family with sequence similarity 101, member B	359845   Hs.345588//Hs.723693//Hs.741149	3.087488137	3.526101376	3.263290536	3.035096233	3.801744161	3.445367787	4.175057776	2.462227326	
17087480 TGFBR1	transforming growth factor, beta receptor 1	7046 Hs.494622	5.393429803	5.686118254	5.635158646	5.187416102	4.274372986	3.846580504	4.760685094	3.613274528	
16697686 ZNF281	zinc finger protein 281	23528   Hs.59757//Hs.703449//Hs.735801	4.995047915	5.258792734	4.931025265	4.946127871	3.947482122	2.960402484	3.415213865	3.202812589	
17005042 JARID2	jumonji, AT rich interactive domain 2	3720 Hs.269059	5.180062982	5.624688409	4.938554667	5.271256414	4.485263402	4.26014185	4.309267077	4.020427574	
16958844 GATA2	GATA binding protein 2	2624 Hs.367725	4.053333063	3.475465536	3.733777387	3.187649609	4.833930766	3.182817214	5.952783685	4.592140373	
16745113 BCL9L	B-­‐cell CLL/lymphoma 9-­‐like	283149 Hs.414740	5.15223938	4.603396345	4.30461649	4.364861808	4.612895027	5.372267855	4.417850361	4.701374708	
17097914 PHF19	PHD finger protein 19	26147 Hs.460124	5.71054616	5.906055797	5.496429844	6.017929008	4.322869765	3.800687166	4.337393079	3.665212587	
17002155 MED7	mediator complex subunit 7	9443   Hs.279902//Hs.744244	4.189975011	4.198475052	3.563058664	4.789741713	4.324027993	3.574697647	3.86241345	3.654772311	
16754060 CNOT2	CCR4-­‐NOT transcription complex, subunit 2	4848   Hs.133350//Hs.730666	3.900221779	4.089504251	4.297770437	4.29312907	3.587939815	3.497552543	4.288947739	3.350114729	
16860865 HPN	hepsin	3249 Hs.182385	1.96721642	2.242271498	2.0984343	1.770403541	2.47382582	2.321275583	3.034038388	2.929725246	
16965642 RBPJ	recombination signal binding protein for immunoglobulin kappa J region	3516 Hs.479396	4.53071624	4.485194088	4.127125452	4.650055702	3.678518792	3.617866547	3.671696147	2.593454944	
16887675 DLX1	distal-­‐less homeobox 1	1745 Hs.407015	4.405636172	4.239695397	4.141813549	4.159628769	5.699618076	4.635965156	4.470747356	4.121917797	
17054243 PDGFA	platelet-­‐derived growth factor alpha polypeptide	5154 Hs.535898	3.741969195	4.315455916	3.86063937	3.992474512	3.604337377	4.022890037	3.744155751	4.494546933	
16740412 LTBP3	latent transforming growth factor beta binding protein 3	4054 Hs.289019	3.486983309	3.971406425	3.41117467	3.701799334	3.82911281	3.814527192	3.246538011	3.684037304	
16851383 GATA6	GATA binding protein 6	2627   Hs.514746//Hs.741506	4.943089423	4.939536229	4.794276774	4.889272043	4.273462438	2.702657268	3.144434179	2.904910019	
16764114 WNT10B	wingless-­‐type MMTV integration site family, member 10B	7480 Hs.91985	2.821812424	2.965991443	2.736244062	3.099492798	3.284781981	2.647531751	3.119187905	3.242676845	
17096827 KLF4	Kruppel-­‐like factor 4 (gut)	9314 Hs.376206	4.002827979	3.41447036	2.761671467	3.183040908	3.394816168	2.7376326	3.374326574	3.02316729	
16897637 RTN4	reticulon 4	57142 Hs.637850	3.94802991	3.699140866	3.756736419	4.238734202	3.278106621	3.643378624	3.620936773	4.013844143	
16677556 TGFB2	transforming growth factor, beta 2	7042 Hs.133379	9.02803553	8.958395862	8.977954859	8.938308343	2.400614653	2.583717093	3.176556304	2.183474225	
16830398 KCTD11	potassium channel tetramerization domain containing 11	147040 Hs.592112	3.564379327	3.711307385	3.448887764	3.447954084	3.444747121	3.34186109	2.694568065	4.702960794	
16919728 ZNF335	zinc finger protein 335	63925 Hs.174193	4.94802189	5.118270124	4.110314269	4.756306649	3.596011944	4.212021981	3.992182858	4.385423616	
17115505 FLNA	filamin A, alpha	2316 Hs.195464	7.092601485	6.688060343	6.447576164	6.493201207	4.197966066	3.402109218	4.543826143	3.409295003	
16728066 LRP5	low density lipoprotein receptor-­‐related protein 5	4041 Hs.6347	4.312275921	4.296653906	4.354416338	4.749664478	2.384813531	2.543442197	3.063924654	4.508337941	
17070013 RDH10	retinol dehydrogenase 10 (all-­‐trans)	157506 Hs.244940	5.252801055	5.075175327	5.097993498	4.330911718	3.056518266	2.713232994	2.689688553	3.118687657	
16837418 SOX9	SRY (sex determining region Y)-­‐box 9	6662 Hs.647409	6.278817138	7.422217224	6.265127551	6.912698802	4.680724159	3.65675942	4.269694139	3.730232193	
16734420 ASCL2	achaete-­‐scute family bHLH transcription factor 2	430 Hs.152475	3.347942871	2.879452712	3.142192079	3.414194119	4.503824653	4.533948991	3.721639185	3.317244085	
16794476 NUMB	numb homolog (Drosophila)	8650   Hs.525443//Hs.654609//Hs.714879	5.517371735	5.18645154	5.052160193	5.00473375	4.096112489	3.606051094	3.512648035	2.731470974	
16681501 CTNNBIP1	catenin, beta interacting protein 1	56998 Hs.463759	4.581417305	4.295080919	3.774686147	4.516032553	2.501735307	2.784124046	2.934332265	2.575334921	
16940924 SEMA3F	sema domain, immunoglobulin domain (Ig), short basic domain, secreted, (semaphorin) 3F	6405 Hs.32981	4.674800583	4.87655501	3.898477094	4.914868691	3.476069733	2.023833624	2.989281932	3.10826939	
16671579 ADAM15	ADAM metallopeptidase domain 15	8751 Hs.312098	5.419471846	5.556508079	5.181384225	5.31269755	4.846303007	1.638024723	1.981343677	2.959369049	
17072295 ZHX2	zinc fingers and homeoboxes 2	22882   Hs.377090//Hs.658443	4.112010156	4.920811969	4.11155033	3.907648731	3.580146136	3.388762751	3.426304784	2.789390382	
16896442 EIF2AK2	eukaryotic translation initiation factor 2-­‐alpha kinase 2	5610 Hs.131431	6.765403527	7.131352103	7.231956363	7.256546607	2.600043923	2.770553638	2.722054803	3.602908032	
16942958 EPHA3	EPH receptor A3	2042 Hs.123642	1.19008291	1.535053871	1.737054676	1.354545587	1.824581886	1.867672064	1.722453183	1.579761726	
17049573 SRRT	serrate RNA effector molecule homolog (Arabidopsis)	51593 Hs.111801	5.721488941	5.151145329	5.288007718	5.25583931	3.401134669	3.763578636	3.990947258	4.14335131	
16972764 ING2	inhibitor of growth family, member 2	3622 Hs.107153	5.671699243	5.804505003	6.167711444	5.64655515	3.064553753	2.727660526	3.478818044	2.857952871	
16697544 ASPM	asp (abnormal spindle) homolog, microcephaly associated (Drosophila)	259266 Hs.121028	6.286061178	6.513755528	6.433879006	6.853984441	2.50180776	2.76507592	2.173277343	2.59347222	
16840919 CTC1	CTS telomere maintenance complex component 1	80169 Hs.156055	4.285601256	4.356671427	4.17880718	4.483486328	3.111228738	2.052104218	3.431997716	2.539018359	
17048228 FZD1	frizzled class receptor 1	8321 Hs.94234	2.650064492	2.591796811	3.508978823	1.960864144	2.062865816	2.362160357	1.839224389	1.802928654	
16706875 BMPR1A	bone morphogenetic protein receptor, type IA	657 Hs.524477	5.113768604	5.084447371	5.127463118	5.333091731	2.939835393	2.066663865	1.90107588	2.532056786	
17111641 AMER1	APC membrane recruitment protein 1	139285 Hs.314225	3.413672381	3.669029331	3.271762299	3.654032748	3.435042762	2.246451673	2.944083045	2.397165854	
16689546 TGFBR3	transforming growth factor, beta receptor III	7049   Hs.482390//Hs.735919	2.649365646	1.861004298	2.421433441	2.042034957	2.239168123	2.211594023	1.777665393	2.485645084	


17046935 FZD9	
frizzled class receptor 9	
8326 Hs.647029	
1.443656383	
1.986206174	
2.157528688	
2.438955125	
4.758715887	
2.768574914	
2.824038404	
4.010164682	
16681749 MAD2L2	MAD2 mitotic arrest deficient-­‐like 2 (yeast)	10459 Hs.19400	5.700613189	5.625038739	5.20016053	5.103122267	4.58071988	3.280293144	5.138140586	4.039460922	
16915091 TFAP2C	transcription factor AP-­‐2 gamma (activating enhancer binding protein 2 gamma)	7022 Hs.473152	5.939494898	6.117335046	5.932997179	6.06350896	2.871470737	2.79429872	2.129182191	3.953606703	
17084866 GLIPR2	GLI pathogenesis-­‐related 2	152007 Hs.493819	5.310288584	5.290610332	5.227096528	4.927364185	4.258355916	4.417098173	4.635593046	3.903732978	
16936452 PLXNB2	plexin B2	23654 Hs.3989//Hs.736016	5.089096343	5.160521387	4.527159966	5.309372241	3.999480118	3.226484612	3.931014503	2.799453711	
16687208 ZCCHC11	zinc finger, CCHC domain containing 11	23318 Hs.655407	5.887613291	5.920930612	5.766606187	5.941532003	3.787533842	3.728171075	3.583746448	3.986225166	
16670114 BCL9	B-­‐cell CLL/lymphoma 9	607 Hs.415209	5.882132282	6.168640682	5.761667694	6.101249939	3.202953895	3.45768234	4.445606796	1.531422063	
17079910 KLF10	Kruppel-­‐like factor 10	7071 Hs.435001	7.901178718	7.409678251	7.840754592	7.31992313	4.61252912	4.438701771	3.625602017	3.75093656	
16873268 ERCC2	excision repair cross-­‐complementation group 2	2068 Hs.487294	6.483876587	5.668163851	5.542834199	5.666239267	4.256752343	2.268874299	2.697990527	3.926609802	
16691668 NOTCH2	notch 2	4853 Hs.487360	5.96979003	6.161867466	5.835725925	5.944357078	2.99432869	3.337719609	3.500701387	2.871215064	
17091928 FOXD4	forkhead box D4	2298 Hs.584759	4.521343159	4.891152076	5.942241503	4.275306783	4.711308782	3.712009031	4.315576567	4.61036833	
16892178 DIS3L2	DIS3 like 3'-­‐5' exoribonuclease 2	129563 Hs.732236	5.190289968	5.169941796	4.935240654	5.550301906	4.08524418	3.208211586	3.616700504	3.973526687	
16906534 STAT1	signal transducer and activator of transcription 1, 91kDa	6772   Hs.642990//Hs.743244	6.38394892	6.430753908	5.973707691	6.350452588	3.548211357	2.295410017	3.493219508	3.290328716	
16693014 VPS72	vacuolar protein sorting 72 homolog (S. cerevisiae)	6944 Hs.2430	5.393281577	5.873419365	5.211513224	5.349398951	3.887262524	3.739589192	4.250949071	3.384666977	
17009093 VEGFA	vascular endothelial growth factor A	7422 Hs.73793	5.197720595	5.358616883	5.237897716	4.995366727	2.998173479	3.829321624	4.282471037	3.274138216	
17068582 HOOK3	hook microtubule-­‐tethering protein 3	84376 Hs.162852	5.242374801	5.759737712	5.533833228	5.96419852	3.738585003	2.705103112	2.717401991	2.128231183	
16987798 FER	fer (fps/fes related) tyrosine kinase	2241 Hs.221472	6.769803572	6.828491063	7.109057128	7.044699171	2.854913429	3.162570113	3.359894978	3.615201257	
16761583 LRP6	low density lipoprotein receptor-­‐related protein 6	4040   Hs.584775//Hs.658913	5.459634392	5.564227555	5.05384708	5.912940283	3.4351362	4.037666656	3.930726685	3.241051929	
16805230 CHD2	chromodomain helicase DNA binding protein 2	1106 Hs.220864	5.414891822	5.589349066	5.683901752	5.408937372	2.958734561	2.621544611	3.596978057	2.354015471	
16730503 YAP1	Yes-­‐associated protein 1	10413 Hs.503692	6.665422382	6.623604767	6.402924765	6.758837358	3.364681766	3.43575139	3.980363106	4.129839936	
17076694 KAT6A	K(lysine) acetyltransferase 6A	7994 Hs.491577	5.669664849	5.809081604	5.73049647	5.729221457	4.079933213	3.69016557	4.554546997	3.651819195	
16671642 EFNA1	ephrin-­‐A1	1942 Hs.516664	4.4152329	4.75042553	4.118456471	4.535477038	2.452593651	2.831752078	3.664206792	3.512047338	
16989750 CTNNA1	catenin (cadherin-­‐associated protein), alpha 1, 102kDa	1495 Hs.445981	2.11926813	1.986633038	2.181505436	2.188853255	2.948436118	2.890524667	3.709417751	3.871459832	
16808345 ELL3	elongation factor RNA polymerase II-­‐like 3	80237 Hs.706346	4.306156742	4.477550402	4.097663705	4.212582201	3.22449544	3.242885429	3.27635248	3.017847728	
16730124 MED17	mediator complex subunit 17	9440 Hs.444931	6.086004368	6.148517032	5.789794113	5.694127465	2.520241963	2.923330693	3.712562404	3.368371054	
16783215 ARHGAP5	Rho GTPase activating protein 5	394 Hs.592313	6.092709499	5.616795576	6.206631806	5.753169023	2.970555648	2.410126153	2.499037309	2.354753033	
16780896 LIG4	ligase IV, DNA, ATP-­‐dependent	3981 Hs.166091	5.492455275	5.421135468	5.569785892	5.440944885	2.236368017	2.310743756	2.761157322	2.526127783	
16792798 SAV1	salvador homolog 1 (Drosophila)	60485   Hs.642842//Hs.706933	6.194937511	6.614238653	6.444940189	6.582352299	1.813367377	2.102850336	2.143055746	2.637912144	
16886503 RIF1	RAP1 interacting factor homolog (yeast)	55183   Hs.655671//Hs.735592	6.178051906	6.404998341	6.496014719	6.522162196	3.527217104	2.555727618	3.41647136	3.200211067	
17092490 NFIB	nuclear factor I/B	4781 Hs.644095	6.547496531	6.625401671	7.049198853	6.679450407	2.710856981	3.512573	2.733937538	3.315903653	
16778715 TPT1	tumor protein, translationally-­‐controlled 1	7178   Hs.374596//Hs.717316	6.005042034	6.000398378	5.318065797	6.10146258	3.670673319	2.278634641	2.818073554	3.283704499	
16953052 SETD2	SET domain containing 2	29072 Hs.517941	6.150215352	5.912680996	6.389885198	6.145383218	3.492169296	3.0917382	3.934975037	2.495920217	
16816200 NDE1	nudE neurodevelopment protein 1	54820 Hs.655378	4.828270282	5.145752198	5.018467814	5.157363761	3.363292667	2.828159882	2.821457267	3.542374025	
16731441 ZBTB16	zinc finger and BTB domain containing 16	7704   Hs.591945//Hs.682144	1.678294971	1.704968387	1.728274542	1.257634481	2.282902161	1.597814097	1.826516584	2.181068206	
16844248 MED24	mediator complex subunit 24	9862 Hs.462983	5.2280307	5.365583836	4.842277499	5.414577183	5.031498971	3.972388563	4.49519452	4.059274772	
17072084 MED30	mediator complex subunit 30	90390 Hs.492612	6.107123163	6.51878756	6.091209803	6.397669013	4.199347592	3.877830004	4.554859541	4.232051636	
16769250 IGF1	insulin-­‐like growth factor 1 (somatomedin C)	3479 Hs.160562	1.598829839	1.250026774	1.881231622	1.261936659	4.165482141	4.2412505	3.877051997	3.253709692	
16858344 SMARCA4	SWI/SNF related, matrix associated, actin dependent regulator of chromatin, subfamily a, member 4	6597 Hs.327527	6.844019239	7.061681656	6.850064985	6.618708904	4.519558278	4.259621904	4.900016783	3.595603975	
16865214 CNOT3	CCR4-­‐NOT transcription complex, subunit 3	4849 Hs.343571	5.822532831	6.180666643	5.719668454	5.614802304	6.152950632	4.155473366	5.177973032	3.884632585	
16667183 MTF2	metal response element binding transcription factor 2	22823 Hs.31016	5.485183864	5.541862027	5.29033785	5.908110978	4.629412094	4.613161628	5.603837605	5.502009855	
17104587 MED12	mediator complex subunit 12	9968 Hs.409226	5.828908355	5.800759094	5.59776599	5.531682623	5.843386151	4.074853337	5.663740274	4.359234021	
17024285 CITED2	Cbp/p300-­‐interacting transactivator, with Glu/Asp-­‐rich carboxy-­‐terminal domain, 2	10370 Hs.82071	3.567819777	3.704015486	3.71598428	3.202544218	6.52847784	5.75662085	7.127512802	4.588821284	
16992622 MSX2	msh homeobox 2	4488 Hs.89404	3.073288296	3.123416296	3.989294886	3.661598314	4.827220579	4.937151468	4.831224233	5.72098179	
16846029 WNT3	wingless-­‐type MMTV integration site family, member 3	7473   Hs.445884//Hs.745220	2.662982111	3.890911175	3.279387303	3.406888154	7.285158746	3.882914373	6.389897419	4.625452093	
16709333 TCF7L2	transcription factor 7-­‐like 2 (T-­‐cell specific, HMG-­‐box)	6934 Hs.593995	5.378416006	5.651688011	4.68317957	5.663330645	6.685307462	4.042150367	7.229182931	6.208174193	
16799231 SPRED1	sprouty-­‐related, EVH1 domain containing 1	161742 Hs.525781	7.391580173	7.43463653	7.752629096	7.398830895	5.82467506	3.558800902	6.427042642	6.039948492	
16832920 NF1	neurofibromin 1	4763 Hs.113577	6.343225318	6.617168214	6.467352994	6.717973683	4.219060674	3.275777918	3.575857629	3.408480059	
17072669 MYC	v-­‐myc avian myelocytomatosis viral oncogene homolog	4609 Hs.202453	7.404113131	7.334735638	6.927471872	7.154037578	5.001530162	3.052799538	4.307559588	2.976060863	
16840732 TP53	tumor protein p53	7157   Hs.437460//Hs.740601	3.519014217	3.743797684	2.813844239	3.515119951	3.660208984	3.626371569	3.721478462	3.470397366	
16777965 N4BP2L2	NEDD4 binding protein 2-­‐like 2	10443 Hs.507680	5.82883616	5.931729168	6.011695398	5.649313212	2.785733247	3.572136071	3.635690076	3.391955922	
17066118 PCM1	pericentriolar material 1	5108 Hs.491148	6.916447119	7.065862002	6.988486234	7.213252766	3.050634481	3.828890265	4.099799646	3.458283729	
16703563 BAMBI	BMP and activin membrane-­‐bound inhibitor	25805 Hs.533336	5.021374707	4.774501331	5.058340385	4.487991146	4.790099432	4.986088835	3.414561327	3.341447583	
16721965 CTR9	CTR9, Paf1/RNA polymerase II complex component	9646 Hs.725151	7.055653781	7.1003263	7.206543337	7.239869763	2.370990369	2.876249905	2.913791938	3.699272536	
16688024 DOCK7	dedicator of cytokinesis 7	85440 Hs.744927	6.538797246	6.878059152	6.678866344	6.837463445	3.087633326	2.748415025	3.651540542	1.991832394	
16965338 MED28	mediator complex subunit 28	80306 Hs.731966	6.54461597	6.648864854	6.261514928	6.616790141	4.329875719	3.552104973	2.664861456	5.231251436	
16845126 STAT3	signal transducer and activator of transcription 3 (acute-­‐phase response factor)	6774 Hs.463059	1.088830626	1.258435444	1.142265786	1.884702151	2.699134378	1.694702959	4.089253832	3.168795356	
16857608 ZNF358	zinc finger protein 358	140467 Hs.133475	6.093878505	6.491218243	6.319680938	6.401607848	3.777994194	3.663185702	4.6316839	4.532166212	
17110193 MED14	mediator complex subunit 14	9282 Hs.407604	6.222088772	6.428943113	6.424827639	6.526996822	3.962963841	3.130497637	4.996284278	4.183285551	
16866337 TRIM28	tripartite motif containing 28	10155 Hs.467408	7.133711491	6.899885237	6.611813223	6.917594955	4.35396086	4.876802686	4.624198211	3.608195724	
16967091 REST	RE1-­‐silencing transcription factor	5978 Hs.307836	6.725574606	6.91914881	6.553108911	6.426734341	3.61826198	4.419427531	4.580917833	3.926896653	
16855093 SMAD2	SMAD family member 2	4087   Hs.12253//Hs.705764//Hs.741342	6.000327671	5.681081005	6.107654736	5.864755027	3.390085219	4.785544802	4.571200569	3.214481884	
16785058 HIF1A	hypoxia inducible factor 1, alpha subunit (basic helix-­‐loop-­‐helix transcription factor)	3091   Hs.597216//Hs.719495	8.476636744	8.720838754	8.813303185	8.659390881	5.687495512	3.466566946	4.83823603	5.062519171	
16844872 JUP	junction  plakoglobin	3728 Hs.514174	5.842798869	5.947057753	5.114359679	5.64448318	4.514329093	2.319417901	3.115745815	2.358833067	
16872203 PAF1	Paf1, RNA polymerase II associated factor, homolog (S. cerevisiae)	54623 Hs.466714	6.45184607	6.863657476	6.348392448	6.295716013	4.891180696	3.535053207	3.910720288	3.23802843	
16799814 RTF1	Rtf1, Paf1/RNA polymerase II complex component, homolog (S. cerevisiae)	23168 Hs.511096	6.958088399	6.977516401	7.004656283	7.236731706	4.833799954	3.854766874	3.907702279	3.750032609	
16661567 PHACTR4	phosphatase and actin regulator 4	65979 Hs.225641	6.755200787	6.843916056	7.103755122	6.647043508	4.175778288	4.017626065	3.525696698	4.157358214	
16796325 DICER1	dicer 1, ribonuclease type III	23405 Hs.87889	5.83986995	6.120342114	6.491773902	6.094032087	4.617986665	4.195664491	4.898684055	3.625880478	
17005276 SOX4	SRY (sex determining region Y)-­‐box 4	6659 Hs.643910	5.704368113	5.943142014	5.412840427	5.559533711	4.298520906	3.650149027	3.343743495	2.734608378	
16662475 AGO3	argonaute RISC catalytic component 3	192669 Hs.657659	5.541825607	5.670495469	5.51877508	5.482493824	3.751985507	3.755825443	3.287169126	3.849928095	
16836375 MSI2	musashi RNA-­‐binding protein 2	124540 Hs.658922	5.188096771	4.897401946	5.193131297	5.084823844	3.631602257	3.809356211	4.725710013	4.154487904	
16852354 SMAD4	SMAD family member 4	4089 Hs.75862	5.850726696	5.677774022	6.090051376	5.682316691	5.297895728	5.779175247	4.272768148	4.563085913	
17077004 SNAI2	snail family zinc finger 2	6591 Hs.360174	8.551822427	7.731873669	8.31767985	7.797582697	4.671560475	4.659818375	4.233967971	3.481259589	
16753943 FRS2	fibroblast growth factor receptor substrate 2	10818 Hs.593446	6.056912022	6.154436669	6.284951892	6.476109662	5.174693126	4.813659263	4.360359099	3.626479994	
16949759 HES1	hes family bHLH transcription factor 1	3280 Hs.250666	7.857827215	7.917490679	7.724706845	8.046471403	3.546115665	3.165550811	4.822607693	3.451671623	
17059119 SEMA3C	sema domain, immunoglobulin domain (Ig), short basic domain, secreted, (semaphorin) 3C	10512 Hs.269109	6.230258826	5.80245724	6.136241085	6.102849182	2.988054774	3.608823955	2.994013204	3.639464047	
16770685 TBX3	T-­‐box 3	6926 Hs.744016	6.598003864	6.078478432	6.628477788	5.928216666	2.598205157	3.42851242	3.871384373	1.715315889	
16879863 EPCAM	epithelial cell adhesion molecule	4072 Hs.542050	7.582554285	7.771363121	7.698678202	8.043772207	2.626413633	2.478326973	2.217772195	2.103249767	
17113658 NKAP	NFKB activating protein	79576 Hs.522771	6.012414284	6.193812287	6.551992956	6.482413962	2.64809044	2.07975556	3.325759934	2.9327713	
16940557 ARIH2	ariadne RBR E3 ubiquitin protein ligase 2	10425 Hs.633601	6.757021294	6.535560267	6.803999407	6.315961336	3.70505148	2.526947735	2.020392041	1.885710416	
16994161 MED10	mediator complex subunit 10	84246 Hs.13885	7.806091005	7.562227245	7.659941578	7.452763704	2.7868926	3.998456019	3.456942141	3.512017525	
16885080 EPB41L5	erythrocyte membrane protein band 4.1 like 5	57669 Hs.369232	4.768841079	5.342393223	4.974536056	5.364079239	2.973446041	3.202091781	3.353027252	2.310946399	
16792181 FOXA1	forkhead box A1	3169 Hs.163484	5.122652362	5.843831202	4.894070375	5.859512363	2.464707936	4.175192758	1.495508377	3.558880539	
16675354 CDC73	cell division cycle 73	79577 Hs.378996	6.841636107	6.957318354	7.31729337	7.094549012	4.064805041	3.126272392	4.211435466	3.021327593	
16660624 KDM1A	lysine (K)-­‐specific demethylase 1A	23028 Hs.591518	8.697236582	8.348490564	8.597542225	8.592628129	4.778445407	3.489319096	4.376668484	3.753265586	
16748751 STRAP	serine/threonine kinase receptor associated protein	11171 Hs.743971	7.781666228	7.874404999	7.784774446	7.763063029	4.856844383	3.988008653	4.544735518	5.214942519	
16957843 GSK3B	glycogen synthase kinase 3 beta	2932 Hs.445733	8.560628072	8.765483605	8.800852501	8.694790068	3.736018506	3.830076744	4.760084397	4.165708919	
16709140 SMC3	structural maintenance of chromosomes 3	9126 Hs.24485	8.056934497	8.23400266	8.310353457	8.339200048	4.721105355	3.907255127	4.653406053	3.987026974	
16890574 XRCC5	X-­‐ray repair complementing defective repair in Chinese hamster cells 5 (double-­‐strand-­‐break rejoinin	7520 Hs.388739	9.371987369	9.46070416	9.449332876	9.638647046	3.63844216	4.306059776	4.377439389	1.834324416	
16809346 LEO1	Leo1, Paf1/RNA polymerase II complex component, homolog (S. cerevisiae)	123169 Hs.567662	7.06745669	7.445063503	7.205025884	7.438940118	2.643604436	1.675542957	3.366184135	2.878160479	
17111219 SMC1A	structural maintenance of chromosomes 1A	8243 Hs.211602	6.934196915	7.286800226	6.736177945	7.051908384	5.461649823	4.769206979	5.631294062	3.730085011	
17106739 STAG2	stromal antigen 2	10735   Hs.496710//Hs.624663	6.20880291	5.956186209	6.439779439	6.456429032	5.082430697	5.489183785	5.049633885	5.460028732	
16984083 NIPBL	Nipped-­‐B homolog (Drosophila)	25836 Hs.481927	7.174127807	7.390052208	7.155536796	7.140283973	5.449222303	5.853474345	5.562536178	4.66057622	
17064105 EZH2	enhancer of zeste homolog 2 (Drosophila)	2146   Hs.444082//Hs.732308	7.058338186	6.973384122	7.233850131	7.446265899	5.709111729	6.285214213	5.81540561	5.684524587	
16823433 CREBBP	CREB binding protein	1387 Hs.459759	7.13973435	7.451311129	7.518920941	7.357351659	5.37160793	4.754376264	5.942521662	5.053908772	
16833139 PSMD11	proteasome (prosome, macropain) 26S subunit, non-­‐ATPase, 11	5717 Hs.443379	7.333115639	7.368798731	7.605126334	7.592792455	5.01944418	2.967948537	5.041976993	4.368205817	
16718950 TIAL1	TIA1 cytotoxic granule-­‐associated RNA binding protein-­‐like 1	7073 Hs.501203	6.781737108	6.780859191	6.404960104	7.024298757	4.10617395	5.211949178	6.012989673	6.452978614	
17008856 SRF	serum response factor (c-­‐fos serum response element-­‐binding transcription factor)	6722 Hs.520140	6.129355319	5.78701131	5.495651134	5.511992529	6.323056203	5.684400891	7.896093815	5.390715306	
16717869 LDB1	LIM domain binding 1	8861 Hs.454418	5.939910932	5.619235512	5.095627693	5.364145078	5.707407501	4.004142317	6.003207218	4.790781083	
16749382 MED21	mediator complex subunit 21	9412 Hs.286145	5.980103685	6.155651425	6.486248551	6.492345125	3.417222693	4.511492278	4.433594352	5.34669272	
16909049 CUL3	cullin 3	8452 Hs.372286	6.580117762	6.515798364	6.123710063	6.288511865	6.039612787	6.61504256	4.71444677	5.646053552	
16939558 CTNNB1	catenin (cadherin-­‐associated protein), beta 1, 88kDa	1499 Hs.476018	7.096658089	7.309106449	7.324941354	7.375113177	5.81066747	5.792435689	6.316684004	5.959848852	
16829667 PAFAH1B1	platelet-­‐activating factor acetylhydrolase 1b, regulatory subunit 1 (45kDa)	5048 Hs.77318	7.124283492	7.267377351	7.348295973	7.514015735	6.148715831	6.641902906	6.836707574	7.194368152	
16877836 RAB10	RAB10, member RAS oncogene family	10890 Hs.467960	7.097716603	7.164891692	7.13885091	7.097603642	6.784762173	5.03784201	6.456876441	6.089856971	
16993268 HNRNPAB	heterogeneous nuclear ribonucleoprotein A/B	3182   Hs.591731//Hs.715055	7.484660879	7.410625772	7.646389813	7.156412438	7.617825313	7.536930517	7.556369135	7.447669561	
16826909 CNOT1	CCR4-­‐NOT transcription complex, subunit 1	23019   Hs.460923//Hs.731691	7.692053357	8.04769192	7.645595592	8.176289183	6.764524608	5.406293431	6.61062771	4.322494676	
