# Supplementary material for: The marrow niche controls the cancer stem cell phenotype of disseminated prostate cancer
Source: Oncotarget. 2016 May 9;7(27):41217–32. doi: 10.18632/oncotarget.9251 (PMC5173053; doi:10.18632/oncotarget.9251)
Supplement: Supplementary file 4 [file oncotarget-07-41217-s004.doc]

Supplemental Table3. Genes differentially expressed between CSC and non-­‐CSC obtained from in vivo DTCs
GeneName	p-­‐value	
1 LINC00657	0.030382822	
2 RPL39	0.030382822	
3 TMEM14A	0.029401048	
4 TMEM159	0.030382822	
5 DSTN	0.030382822	
6 NAA20	0.030382822	
7 RFC3	0.030382822	
8  RP4-­‐779E11.3	0.030382822	
9 ARL6IP4	0.030382822	
10 SMCR9	0.030382822	
11 KLHL30	0.030382822	
12 PHYHD1	0.030382822	
13 FAM188B2	0.030382822	
14 SLC6A7	0.030382822	
15 FAM216B	0.030382822	
16 MRPL19	0.030382822	
17 NFATC2	0.030382822	
18 CRB3	0.030382822	
19 SLC22A12	0.030382822	
20 ZFP62	0.030382822	
21 AP1B1	0.030382822	
22 SLC25A35	0.030382822	
23 GAS8	0.030382822	
24 SEC24B	0.030382822	
25 ITGB7	0.030382822	
26 MPND	0.030382822	
27 ABHD17AP3	0.030382822	
28 MLKL	0.030382822	
29 LOC100506578	0.030382822	
30 KLK1	0.030382822	
31 RIMKLA	0.029401048	
32 OBSCN	0.030382822	
33 TMEM59	0.030382822	
34 CNOT6	0.030382822	
35 LOC284014	0.030382822	
36 CCDC58	0.030382822	
37 CMC2	0.030382822	
38 FBXO32	0.030382822	
39 COX7B	0.030382822	
40 TARS	0.030382822	

41 H2BFM	0.030382822	
42 MYH3	0.030382822	
43 SLC2A4	0.030382822	
44 MIR548Q	0.030382822	
45 HGF	0.030382822	
46 MYO6	0.030382822	
47 BMP8B	0.030382822	
48 FAM200A	0.030382822	
49 IKZF5	0.030382822	
50 PTGER4P2-­‐CDK2AP2P2	0.030382822	
51 TLR8-­‐AS1	0.030382822	
52 DDX58	0.030382822	
53 KRTAP23-­‐1	0.030382822	
54 CCL22	0.030382822	
55 EZR	0.030382822	
56 NAP1L1	0.030382822	
57 RASIP1	0.030382822	
58 APCDD1	0.030382822	
59 NOTCH2NL	0.030382822	
60 MEF2C	0.029401048	
61 LPIN1	0.030382822	
62 WFDC10A	0.030382822	
63 AP5S1	0.030382822	
64 ARHGAP9	0.030382822	
65 AQP2	0.030382822	
66 CCDC27	0.030382822	
67 CRNKL1	0.029401048	
68 DONSON	0.030382822	
69 LOC100507654	0.030382822	
70 TMEM255B	0.030382822	
71 WEE2	0.030382822	
72 LOC339874	0.030382822	
73 SNORA72	0.030382822	
74 DKKL1	0.042066412	
75 EOGT	0.030382822	
76 NBEA	0.030382822	
77 ATP6V1B1	0.030382822	
78 NT5DC1	0.030382822	
79 AC073128.10	0.030382822	
80 ANAPC13	0.030382822	
81 CAMK2N2	0.030382822	
82 LINGO1	0.030382822	

83 RP11-­‐178C3.2	0.030382822	
84 LINC00167	0.030382822	
85 LRRC18	0.030382822	
86  SORCS3-­‐AS1	0.030382822	
87 DUSP18	0.030382822	
88 FSTL4	0.030382822	
89 SNORD114-­‐27	0.030382822	
90 B2M	0.030382822	
91 OR52E2	0.030382822	
92 PTPN18	0.030382822	
93 RP11-­‐503C24.4	0.030382822	
94 HNRNPH3	0.030382822	
95 MIRLET7I	0.042066412	
96 TPST1	0.030382822	
97 ZBTB38	0.030382822	
98 ANXA2R	0.030382822	
99 ZNF408	0.030382822	
100 MIR218-­‐1	0.030382822	
101 ANKRD30BP3	0.030382822	
102 XAF1	0.030382822	
103 TBC1D29	0.030382822	
104 UQCRC2	0.030382822	
105 ANXA7	0.030382822	
106 P4HB	0.030382822	
107 GPNMB	0.030382822	
108 SPATA21	0.030382822	
109 SLC50A1	0.030382822	
110 ARHGAP23	0.030382822	
111 FFAR1	0.030382822	
112 RHOV	0.030382822	
113 FOLR1	0.030382822	
114 ASS1	0.030382822	
115 ULK1	0.030382822	
116 TM9SF2	0.030382822	
117 HPRT1	0.030382822	
118 MNT	0.030382822	
119 CUL3	0.030382822	
120 HLA-­‐DQB2	0.030382822	
121 SOCS3	0.030382822	
122 MEIS3	0.030382822	
123 TPP2	0.030382822	
124 CCT2	0.030382822	

125 ARHGEF10	0.030382822	
126 GLRX3	0.030382822	
127 SLC6A1	0.030382822	
128 ZBTB47	0.030382822	
129 IL15RA	0.030382822	
130 LINC00544	0.030382822	
131 LINC00645	0.030382822	
132 SYF2	0.030382822	
133 STMN1	0.030382822	
134 ELP6	0.030382822	
135 ZNF280C	0.030382822	
136 GEMIN8	0.030382822	
137 TTC12	0.030382822	
138 GUSBP5	0.030382822	
139 IL31RA	0.030382822	
140 AIM1L	0.030382822	
141 PIK3R2	0.030382822	
142 DANCR	0.030382822	
143 TMEM33	0.030382822	
144 AC019118.2	0.030382822	
145 APAF1	0.030382822	
146 MIR708	0.030382822	
147 FANCI	0.030382822	
148 SIRT4	0.030382822	
149 ZNF74	0.030382822	
150 LTN1	0.030382822	
151 SVOPL	0.030382822	
152 DCSTAMP	0.030382822	
153 MIR1288	0.030382822	
154 RP11-­‐575F12.1	0.030382822	
155 SHPRH	0.030382822	
156 RP11-­‐723O4.2	0.029401048	
157 AURKC	0.030382822	
158 LGR5	0.030382822	
159 MYB	0.042066412	
160 LINC00299	0.030382822	
161 COLGALT2	0.030382822	
162 TMEM60	0.030382822	
163 IL36G	0.030382822	
164 IL21-­‐AS1	0.030382822	
165 MMP16	0.030382822	
166 NAALAD2	0.030382822	

167 CEP68	0.030382822	
168 TMEM167A	0.030382822	
169 CLEC14A	0.030382822	
170 MIR520A	0.030382822	
171 IBTK	0.030382822	
172 MESTIT1	0.030382822	
173 SEBOX	0.030382822	
174 LOC100291105	0.030382822	
175 FLJ31183	0.030382822	
176 TP53BP2	0.042066412	
177 RHOBTB3	0.030382822	
178 FERMT2	0.030382822	
179 HOXC10	0.030382822	
180 HTR2A	0.030382822	
181 SIRPB2	0.030382822	
182 KCNC1	0.030382822	
183 KIAA2022	0.030382822	
184 ZSCAN26	0.030382822	
185 LINC00871	0.030382822	
186 EFNA5	0.030382822	
187 OSTCP1	0.030382822	
188 LOC645752	0.030382822	
189 OR6M1	0.030382822	
190 MIR635	0.030382822	
191 PRC1	0.030382822	
192 CDC37L1	0.042066412	
193 GPR149	0.030382822	
194 RPL12	0.030382822	
195 ADH7	0.030382822	
196 SMC1B	0.030382822	
197 NIT1	0.030382822	
198 USP6	0.030382822	
199 MIR4464	0.042066412	
200 EPCAM	0.030382822	
201 UQCRB	0.030382822	
202 FAP	0.030382822	
203 SAMD9	0.030382822	
204 OR4A15	0.030382822	
205  DLEU7-­‐AS1	0.030382822	
206 FOLH1B	0.030382822	
207 MDGA2	0.030382822	
208 LINC00597	0.030382822	

209 NDUFB2	0.030382822	
210 TRIM49	0.030382822	
211 FAM177B	0.030382822	
212 OR3A3	0.030382822	
213 BCMO1	0.030382822	
214 CLIC2	0.029401048	
215 LOC100506191	0.030382822	
216 FBXO47	0.030382822	
217 OR2V2	0.030382822	
218 LINC01232	0.030382822	
219 FAIM	0.030382822	
220 TEX11	0.030382822	
221 LOC100506082	0.030382822	
222 CENPK	0.030382822	
223 ZGRF1	0.030382822	
224 SNORD116-­‐11	0.030382822	
225 LINC01139	0.030382822	
226  MIR3198-­‐1	0.030382822	
227 ADH1B	0.030382822	
228 ZNF847P	0.030382822	
229 TMEM121	0.030382822	
230 CCDC63	0.030382822	
231 RP11-­‐260E18.1	0.030382822	
232 B3GNT4	0.030382822	
233 RAD51C	0.042066412	
234 LINC00889	0.030382822	
235 NUDT19	0.029401048	
236 SUCO	0.030382822	
237 MIR548AL	0.030382822	
238 THAP6	0.030382822	
239 MIR4527	0.030382822	
240 SFRP2	0.030382822	
241 TAS2R20	0.030382822	
242 MIR215	0.042066412	
243 SNORD115-­‐31	0.030382822	
244 SNORD114-­‐1	0.030382822	
245 CHEK2	0.029401048	
246 OSER1	0.030382822	
247 HSD3B2	0.030382822	
248 LANCL1	0.030382822	
249 TEKT5	0.030382822	
250 OR8K5	0.030382822	

251 PDXDC2P	0.030382822	
252 MFSD8	0.042066412	
253 RSPH1	0.030382822	
254 EGFR-­‐AS1	0.030382822	
255 METTL7A	0.030382822	
256 RNF216P1	0.030382822	
257 CNTN3	0.030382822	
258 KCTD6	0.030382822	
259 USP48	0.030382822	
260 ZNF284	0.030382822	
261 FFAR4	0.030382822	
262 IGSF10	0.030382822	
263 PDZD3	0.030382822	
264 CD209	0.030382822	
265 DEFB119	0.030382822	
266 LINC01150	0.030382822	
267 LAMA2	0.030382822	
268 LOC646719	0.030382822	
269 OVCH1	0.030382822	
270 LIPF	0.029401048	
271 CCR4	0.030382822	
272 COA1	0.030382822	
273 KLK12	0.030382822	
274 LINC01020	0.030382822	
275 ZC3H12B	0.030382822	
276  ZNF674-­‐AS1	0.030382822	
277 BTBD16	0.029401048	
278  KCTD21-­‐AS1	0.030382822	
279 ZNF345	0.030382822	
280 MIR4664	0.030382822	
281 C6orf89	0.030382822	
282 CREM	0.030382822	
283 C11orf70	0.030382822	
284 YAF2	0.030382822	
285 C17orf104	0.030382822	
286 IFT122	0.030382822	
287 HRK	0.030382822	
288 SCEL	0.030382822	
289 RAD1	0.030382822	
290 CUX2	0.030382822	
291 FTSJ2	0.030382822	
292 EXO1	0.030382822	

293 LINC00424	0.030382822	
294 CLCN1	0.030382822	
295 RYK	0.030382822	
296 BNC2	0.030382822	
297 ARHGAP18	0.030382822	
298 MRPS11	0.030382822	
299 PIP5K1B	0.030382822	
300 CDKN2AIP	0.030382822	
301 PCNX	0.030382822	
302 FLJ45743	0.030382822	
303 FLJ32255	0.030382822	
304 OR2H1	0.030382822	
305 GOLPH3L	0.042066412	
306 WFDC3	0.030382822	
307 POLR3G	0.030382822	
308 MIR581	0.030382822	
309 SDHD	0.030382822	
310 LIPK	0.030382822	
311 RP11-­‐175K6.1	0.030382822	
312 CD163L1	0.030382822	
313 USP33	0.030382822	
314 CRYAB	0.030382822	
315 KCNH1	0.030382822	
316 ANP32D	0.030382822	
317 CAMK1G	0.030382822	
318 KLHDC1	0.030382822	
319 LINC00615	0.030382822	
320 C8orf48	0.030382822	
321 HUS1B	0.030382822	
322 C9	0.030382822	
323 HPGDS	0.030382822	
324 NEK5	0.030382822	
325 FUT8	0.030382822	
326 LOC256374	0.030382822	
327 SLC25A26	0.030382822	
328 LDHC	0.030382822	
329 PLRG1	0.030382822	
330 ZNF140	0.030382822	
331 SLC26A9	0.030382822	
332 MEPE	0.030382822	
333 PNMA1	0.030382822	
334 VPS45	0.030382822	

335 ZNF589	0.030382822	
336 CAPNS2	0.030382822	
337 SLED1	0.030382822	
338 MIR3162	0.030382822	
339 PARP2	0.030382822	
340 MIR3671	0.030382822	
341 MSL3P1	0.030382822	
342 HEMGN	0.030382822	
343 SPATA42	0.030382822	
344 OR4P4	0.030382822	
345 MIR4796	0.030382822	
346 ZNF239	0.030382822	
347 MIR3146	0.042066412	
348 LRCH2	0.030382822	
349 CCDC91	0.029401048	
350 TNFAIP6	0.030382822	
351 C16orf52	0.042066412	
352 TCAM1P	0.030382822	
353  PCDH9-­‐AS3	0.030382822	
354 ING1	0.030382822	
355 BCO2	0.029401048	
356 ZNF24	0.030382822	
357 LRRD1	0.030382822	
358 ZCWPW1	0.030382822	
359 PAQR3	0.030382822	
360 RP11-­‐1080G15.1	0.042066412	
361 QTRTD1	0.042066412	
362 SLC35F1	0.030382822	
363 GCNT6	0.030382822	
364 LINC00515	0.030382822	
365 RNF148	0.030382822	
366 CYP2C8	0.030382822	
367 LINC00619	0.030382822	
368 ADAM22	0.030382822	
369 FAM206A	0.030382822	
370 TMED6	0.030382822	
371 SCGB1D1	0.030382822	
372 TAS2R9	0.030382822	
373 GCH1	0.030382822	
374 LOC100506563	0.030382822	
375 MYBPH	0.030382822	
376 DUOX2	0.030382822	

377 GNG8	0.030382822	
378 SNORD116-­‐12	0.030382822	
379 ROBO1	0.030382822	
380 LOC284837	0.030382822	
381 PTGDR	0.030382822	
382 MIR4798	0.030382822	
383 SERPINB6	0.030382822	
384 WDR1	0.030382822	
385 GCKR	0.030382822	
386 TMEM207	0.030382822	
387 TRPV2	0.030382822	
388 FEV	0.030382822	
389 WDR46	0.030382822	
390 COL24A1	0.030382822	
391 DNAJB2	0.030382822	
392 MAGEA8	0.029401048	
393 CLUL1	0.030382822	
394 LOC284648	0.030382822	
395 KIAA0368	0.030382822	
396 SH2D5	0.030382822	
397 MAGEE1	0.030382822	
398 STAG1	0.030382822	
399 CANT1	0.030382822	
400 DHCR24	0.030382822	
401 TNFSF9	0.030382822	
402 TYRO3	0.030382822	
403 BRD9	0.030382822	
404 RP11-­‐319E16.1	0.030382822	
405 TRABD2A	0.030382822	
406 LOC100288152	0.030382822	
407  ZNF582-­‐AS1	0.030382822	
408 SH3GLB1	0.030382822	
409 LOC100653515	0.030382822	
410 MAP7D2	0.030382822	
411 ATP6V0E2	0.030382822	
412 CTTN	0.029401048	
413 FOXB2	0.030382822	
414 UPP2	0.030382822	
415 C3orf70	0.030382822	
416 PEX26	0.030382822	
417 BAIAP2L2	0.030382822	
418 TMEM9	0.030382822	

419 CAV3	0.030382822	
420 FLJ20518	0.030382822	
421 ADAMTS14	0.030382822	
422 NEGR1	0.030382822	
423 DDX42	0.030382822	
424 FOXD2	0.030382822	
425 SDF4	0.030382822	
426 SHROOM4	0.030382822	
427 LOC283731	0.030382822	
428 SLC37A2	0.030382822	
429 SEPHS1	0.030382822	
430 SNX5	0.030382822	
431 UBE2A	0.030382822	
432 C11orf49	0.030382822	
433 LOC100996455	0.030382822	
434 TBL1Y	0.030382822	
435 B4GALT7	0.030382822	
436 MLLT3	0.030382822	
437 ZFYVE1	0.030382822	
438 SLC35A3	0.029401048	
439 SLC39A5	0.030382822	
440 KRT33B	0.030382822	
441 ARNT2	0.030382822	
442 LINC00696	0.042066412	
443 C1orf213	0.030382822	
444 LDB3	0.030382822	
445 ZFP64	0.030382822	
446 TIMP1	0.030382822	
447 DUSP7	0.030382822	
448 GALNT14	0.030382822	
449 GPR50	0.030382822	
450 EPHA2	0.030382822	
451 HGC6.3	0.030382822	
452 RP11-­‐305P22.9	0.030382822	
453 C4orf50	0.030382822	
454 LINGO3	0.030382822	
455 TRIM62	0.030382822	
456 RGL2	0.030382822	
457 TERT	0.030382822	
458 RP11-­‐95H3.1	0.030382822	
459 LINC00969	0.030382822	
460 TIMM17B	0.030382822	

461 OR13C4	0.030382822	
462 LOC399884	0.030382822	
463 CA10	0.030382822	
464 CLIC6	0.030382822	
465 FKBP1AP1	0.030382822	
466 NDNF	0.030382822	
467 CAMKMT	0.030382822	
468 LZTS1-­‐AS1	0.030382822	
469 ACYP2	0.030382822	
470 ANAPC1P1	0.030382822	
471 SRSF12	0.030382822	
472 LOC100129617	0.030382822	
473 ZNF491	0.030382822	
474 BPIFC	0.030382822	
475 WDR27	0.029401048	
476 CDH13	0.030382822	
477 GSN	0.030382822	
478 YAP1	0.042066412	
479 CECR5	0.030382822	
480 RP11-­‐445H22.3	0.030382822	
481 PAAF1	0.030382822	
482 ZNF511	0.030382822	
483 TMEM45A	0.030382822	
484 DPP8	0.030382822	
485 MRPL15	0.030382822	
486 C7orf31	0.030382822	
487 MRC1	0.030382822	
488 PREP	0.030382822	
489 IFI27L1	0.030382822	
490 PDXK	0.030382822	
491 DAO	0.030382822	
492 CECR7	0.030382822	
493 PHF11	0.030382822	
494 RMI2	0.030382822	
495  RNF157-­‐AS1	0.030382822	
496 CTTNBP2	0.042066412	
497 SCML2	0.030382822	
498 PAGE2	0.030382822	
499 PTPN1	0.030382822	
500 ADAMTS9	0.030382822	
501 PROX2	0.030382822	
502 SAA1	0.030382822	

503 TDGF1P3	0.030382822	
504 MC2R	0.030382822	
505 GLB1L	0.030382822	
506 PSMD7	0.030382822	
507 LOC100130579	0.030382822	
508 ABHD3	0.030382822	
509 MIRLET7C	0.030382822	
510 EDEM3	0.030382822	
511 ASB11	0.030382822	
512 LOC100505588	0.030382822	
513 DEFB123	0.030382822	
514 RPUSD2	0.030382822	
515 C16orf93	0.030382822	
516 SPATA31B1	0.030382822	
517 PDE4DIP	0.030382822	
518 CXorf38	0.030382822	
519 DSCR4	0.030382822	
520 REXO2	0.030382822	
521 STARD13	0.030382822	
522 ACPT	0.030382822	
523 LIG3	0.030382822	
524 OR51E1	0.030382822	
525 ADRA2B	0.030382822	
526 EDNRB	0.030382822	
527 CPSF4L	0.030382822	
528 PPP1R1B	0.030382822	
529 C7orf50	0.030382822	
530 FLJ36000	0.030382822	
531 ZCCHC17	0.030382822	
532 C11orf57	0.030382822	
533 DNAH17	0.030382822	
534 GJC3	0.030382822	
535 NDUFA9	0.030382822	
536 ZNF826P	0.030382822	
537 THOC5	0.030382822	
538 OR10AD1	0.030382822	
539 TKTL1	0.030382822	
540 MFAP2	0.030382822	
541 RLBP1	0.030382822	
542 HOMER3	0.030382822	
543 OR7G2	0.030382822	
544 POLE2	0.030382822	

545 KRT74	0.030382822	
546 OR10G7	0.030382822	
547 EMBP1	0.030382822	
548 CES3	0.029401048	
549 FOXG1	0.030382822	
550 ZNF792	0.042066412	
551 LOC390937	0.030382822	
552 ZCCHC8	0.030382822	
553 FAM131A	0.029401048	
554 LYPD6B	0.030382822	
555 T	0.030382822	
556 ADCY3	0.030382822	
557 MAD1L1	0.030382822	
558 PSME2	0.030382822	
559 FAM50A	0.030382822	
560 TNNT3	0.029401048	
561 GPR157	0.030382822	
562 SLC25A18	0.042066412	
563 GAP43	0.030382822	
564 POLD1	0.030382822	
565 SEPT6	0.030382822	
566 SOX21	0.042066412	
567 H2AFY	0.030382822	
568 NRBP2	0.030382822	
569 DNAJC11	0.030382822	
570 FLNB	0.030382822	
571 SNAI2	0.030382822	
572 CD37	0.030382822	
573 CCL19	0.030382822	
574 ZNF300P1	0.029401048	
575 MGC45922	0.030382822	
576 WBSCR27	0.030382822	
577 AVP	0.030382822	
578 VARS2	0.030382822	
579 RPL36AL	0.030382822	
580 C1orf95	0.030382822	
581 GSTTP1	0.030382822	
582 MIR4725	0.030382822	
583 CRBN	0.030382822	
584 CD74	0.030382822	
585 ERH	0.030382822	
586 STARD7-­‐AS1	0.030382822	

587 RIMBP3C	0.030382822	
588 MIR557	0.030382822	
589  KRTAP10-­‐6	0.030382822	
590 NCCRP1	0.042066412	
591 NICN1	0.030382822	
592 MIR3972	0.030382822	
593 ATP2A1	0.030382822	
594 KCNC3	0.030382822	
595 HIST1H2BO	0.030382822	
596 CATIP	0.030382822	
597 RUNX2	0.030382822	
598 SNIP1	0.030382822	
599 IGHG2	0.030382822	
600 RXFP4	0.030382822	
601 CIB1	0.030382822	
602 RP11-­‐402G3.3	0.030382822	
603 C2CD4D	0.030382822	
604 HTT-­‐AS	0.030382822	
605 HBM	0.030382822	
606 LOC644841	0.030382822	
607 C6orf1	0.030382822	
608 GNA13	0.030382822	
609 MINK1	0.030382822	
610 MYO1C	0.030382822	
611 TAS1R3	0.030382822	
612 COX4I1	0.030382822	
613 RPL26	0.030382822	
614 RPL10A	0.030382822	
615 SNRPD2	0.030382822	
616 MIR548T	0.030382822	
617 NMT1	0.030382822	
618 ND6	0.030382822	
619 TMSB10	0.030382822	
